# Feasibility, accuracy, and effect of a rapid point-of-care serological test (SeroSelectTB) to identify presumptive pulmonary TB patients for confirmatory testing in Ethiopia, South Africa, and Tanzania: a multicenter, open-label, parallel-group, randomized, controlled trial

**DOI:** 10.1016/j.eclinm.2026.103914

**Published:** 2026-04-25

**Authors:** Miloje Savic, Grant Theron, Kidist Bobosha, Balthazar Melchior Nyombi, Ida Laake, Aleksandar Josifoski, Jordancho Arsov, Tamirat Assefa, Jemrath Bikombo, Nick Borain, Jovan Davchev, Stephan Grunwald, Yonas Abebe Habtesilase, Debora Charles Kajeguka, Kristin Kremer, Flavia Mayo, Andrew Medina-Marino, Sasho Najdov, Anna Olutoyin Okunola, Arnold Japhet Ndaro, Welile Vumile Nwamba, Gaudensia Alois Olomi, Julianne du Plessis, Hadija Hamisi Semvua, Tim Welsink, Mekdelawit Wondiyfraw, Melese Yeshambaw, Samuel Asmamaw Yesuf, Solomon Abebe Yimer, Carol Church Holm-Hansen

**Affiliations:** a Aether Dynamics Consulting & Trading GmbH, Vienna, Austria; b Division of Molecular Biology and Human Genetics, Faculty of Medicine and Health Sciences, DSI-NRF Centre of Excellence for Biomedical Tuberculosis Research, Centre for Tuberculosis Research, Stellenbosch University, Cape Town, South Africa; c Research Division of Mycobacterial and Other Bacterial Diseases, Armauer Hansen Research Institute, Addis Ababa, Ethiopia; d School of Medicine, KCMC University, Moshi, Tanzania; e Division of Infection Control, Norwegian Institute of Public Health, Oslo, Norway; f Lateral Flow Laboratories (Pty) Ltd, Muizenberg, South Africa; g InVivo BioTech Services GmbH, Hennigsdorf, Germany; h KNCV Tuberculosis Foundation, The Hague, the Netherlands; i Health Department, Kilimanjaro Regional Administrative Secretary’s Office, Moshi, Tanzania; j Research and Development Division, Coalition for Epidemic Preparedness Innovations (CEPI), Oslo, Norway

**Keywords:** Tuberculosis, Point-of-care testing, Serology, Diagnostic delay, Randomized trial

## Abstract

**Background:**

The feasibility, accuracy and effect of a rapid point-of-care serological test, SeroSelectTB, was assessed to identify presumptive pulmonary tuberculosis (TB) patients at primary healthcare facilities in Ethiopia, South Africa, and Tanzania.

**Methods:**

We conducted a multicenter, open-label, parallel-group, randomized controlled trial from 2020 through 2025. Adults were recruited at selected healthcare facilities with symptoms suggestive of active pulmonary TB. Eligible participants were randomized to standard-of-care (SOC, routine clinical investigation) or intervention (screening with SeroSelectTB) study arms. Our primary outcome was time-to-treatment initiation, calculated as the time from enrollment to treatment initiation. Data were analyzed using intention-to-treat. This trial was registered with Clinicaltrials.gov (NCT04752592) on 2 April 2021.

**Findings:**

Between September 21, 2021, and June 19, 2025, 9097 presumptive pulmonary TB patients were randomly assigned to the SOC (n = 4545) and intervention (n = 4552) study arms. Pulmonary TB was confirmed in 1087 (11.5%) participants (SOC = 527; Intervention = 560). SeroSelectTB was associated with a shorter time-to-treatment initiation compared to SOC (crude hazard ratio (HR): 1.20; 95% CI: 1.06–1.35). The median time-to-treatment initiation among patients with confirmed TB in South Africa, Ethiopia, and Tanzania was 1, 3 and 3 days in the intervention arm compared to 2, 5, and 6 days in SOC arm, respectively. The SeroSelectTB sensitivity was 75.9% and specificity 49.5% in a high HIV setting, whereas sensitivity and specificity were 68.8% and 76.0% among HIV negative participants, respectively.

**Interpretation:**

SeroSelectTB reduces the time-to-treatment initiation for pulmonary TB patients and could be considered for integration into TB testing algorithms at primary healthcare facilities. Rapid tests with a lower than optimal specificity can serve an important role as triage tools when adequate sensitivity is demonstrated.

**Funding:**

This project (RIA2018D-2493, SeroSelectTB) is part of the EDCTP2 Programme supported by the European Union with funding from UK National Institute for Health and Care Research (NIHR). NIHR is funded by the Department of Health and Social Care. The NIHR Global Health Research portfolio supports high-quality applied health research for the direct and primary benefit of people in low- and middle-income countries, using international development funding from the UK Government. The views expressed in this publication are those of the authors and not necessarily those of the EDCTP, NIHR or the UK government.

**Research in context**

**Evidence before this study**

We searched PubMed for English-language publications from January 2000 to August 2025 using the terms “tuberculosis”, “triage test”, “point-of-care”, “serological test”, “lateral flow”, “non-sputum”, and “diagnostic delay”. We found no randomized controlled trials evaluating a point-of-care serological triage test for tuberculosis (TB). Existing triage practices rely on symptoms, imaging, and blood C-reactive protein testing, most of which have suboptimal sensitivity and specificity or are poorly suited to primary healthcare facilities where most patients with TB first seek care. WHO defined the target product profile (TPP) for non-sputum-based TB point-of-care tests to have a minimum sensitivity of 65% and specificity of 98%, rapid turnaround time, and usability in facilities without electricity or laboratory capacity. Our search did not identify any publication with a product that fulfills the WHO TPP. No prior studies have used SeroSelectTB, a test that identifies people at risk of TB and is suitable for use at the primary healthcare level. SeroSelectTB identifies presumptive TB patients who need crucial yet expensive and limited confirmatory molecular tests.

**Added value of this study**

This multicenter, open-label, parallel-group, randomized controlled trial is, to our knowledge, the first to evaluate the feasibility, diagnostic performance, and clinical impact of a non-sputum-based, point-of-care serological triage test for TB. Conducted in Ethiopia, South Africa, and Tanzania, this large study found integration of the SeroSelectTB test at the primary healthcare level reduced time from first presentation to TB treatment initiation by approximately 20% overall (hazard ratio 1·20, 95% CI 1·06–1·35) compared with standard-of-care, though country-specific differences were observed. Median time-to-treatment among confirmed TB cases was shortened by 1–3 days in the intervention arm across all countries. The test required only finger-prick blood, produced results within 15 min, and was implemented by primary healthcare staff with minimal training and in facilities without cold-chain or laboratory equipment. These findings demonstrate that SeroSelectTB meets WHO criteria for a triage test deployable in resource-limited settings, and that it can be operationally integrated into existing TB diagnostic workflows to accelerate treatment initiation.

**Implications of all the available evidence**

Our findings indicate that the addition of SeroSelectTB to diagnostic algorithms can reduce diagnostic and treatment delays among symptomatic individuals presenting to primary healthcare facilities. By identifying those most likely to have TB and prioritizing them for confirmatory testing, such an approach could reduce pre-treatment loss to follow-up, improve linkage to care, and optimize use of molecular diagnostics. When combined with same-day confirmatory testing, widespread adoption of a test like SeroSelectTB could reduce health systems’ delays and contribute meaningfully to the fight against TB.

## Introduction

Tuberculosis (TB) remains a leading public health burden. Globally, an estimated 10.7 million people fell ill with TB (incident cases) and 1.23 million died of TB in 2024.^1^ Achieving the WHO End-TB Strategy targets of 80% reduction in incidence and 90% reduction in mortality by 2030 requires closing key diagnostic gaps.^2^

Many presumptive TB patients who self-present to healthcare facilities do not receive a rapid molecular diagnostic test. Conversely, many symptomatic people, constituting the majority of those with presumptive TB, do not have TB, and are often tested at considerable expense using limited national health budgets. Consequently, it is imperative to implement screening tests that can identify those who stand to benefit the most from expensive confirmatory testing.

WHO has encouraged the development of non-sputum-based TB screening tests to triage symptomatic patients for confirmatory testing, and rule-out TB in people unlikely to benefit from further investigation^3^ According to WHO’s Target Product Profile (TPP), such a test should be affordable to low-resources health budgets, have at least a 65% sensitivity, require little technical expertise, be rapid, minimally invasive, and available to people at the point-of-care (POC)—ultimately satisfying the ASSURED criteria (i.e., Affordable, Sensitive, Specific, User-friendly, Rapid/robust, Equipment-free, and Delivered to those who need it).^4^^,^^5^ However, no such POC TB test to triage symptomatic patients for confirmatory testing currently exists.

In response to these needs, SeroSelectTB, a novel rapid lateral-flow serological triage test for active TB, was developed. SeroSelectTB detects antibodies to a selected combination of *Mycobacterium tuberculosis* (Mtb) antigens that are specific for active TB disease whereas previous antibody-detection TB tests have been based on a single Mtb antigen. The test requires finger-prick blood, is rapid, equipment-free, and suitable for use in low-resource clinics.^6^ In laboratory-based studies that screened more than 300 well-characterized serum samples, SeroSelectTB achieved sensitivities of 84–94% among those living with TB and specificities of 97–100% among healthy endemic controls.^7^ However, while these data suggest that SeroSelectTB performance satisfies the WHO target product profile (TPP) benchmarks for a screening POC test, there are no data on patient outcomes and performance in real-world settings, which are required for potential adoption and scale-up of SeroSelectTB.

To address this data and knowledge gap, we conducted a study to assess the implementation and the diagnostic accuracy of SeroSelectTB, as well as the impact of SeroSelectTB on healthcare system delay. By comparing SeroSelectTB to standard diagnostic pathways across diverse settings and multiple countries, we aimed to determine whether SeroSelectTB can reduce diagnostic delay and pre-treatment loss-to-follow-up by expediting referral, confirmatory testing, and timely initiation of treatment.

## Methods

### Study design and participants

The SeroSelectTB trial was an investigator-initiated, multicenter, open-label, parallel-group, randomized controlled trial conducted in Ethiopia, South Africa, and Tanzania. The trial was implemented at government healthcare facilities in the Northern Health Sub-district of Cape Town, Western Cape Province and Buffalo City Metro (BCM) Health District, Eastern Cape Province in South Africa, Moshi Municipal, Moshi and Hai Districts in Tanzania, and Dale and Aleta Chuko Districts, Sidama Region in Ethiopia (for details see Supplements). Eligibility criteria included 1) aged 18 years and older, 2) clinical symptoms suggestive of TB, and 3) written informed consent. Individuals who had received TB treatment within the previous 60 days were excluded.

All participants with confirmed TB by Xpert MTB/RIF Ultra or Xpert MTB/RIF (Cepheid, Sunnyvale CA, USA), referred to as Xpert unless specified,^8^ and/or acid-fast bacilli (AFB microscopy) received country-specific programmatic treatment.^9^, ^10^, ^11^, ^12^ Study staff had no role in patient management.

### Ethics

Ethical approvals were obtained from the Regional Committee for Medical Research Ethics South East Norway (REK South East, ref # 60638), and national and institutional ethics committees in each participating country (see Supplements for ethical approval documentation). All participants provided signed informed consent prior to enrollment. The SeroSelectTB trial is registered with ClinicalTrials.gov (NCT04752592). Community engagement materials were designed and made available at all study sites and to community healthcare workers in the catchment areas.

### Randomization

Consenting participants were randomized 1:1 to receive routine clinical investigation (standard-of-care; SOC) or screening with SeroSelectTB (intervention). Randomization occurred in block sizes of 4 using a secure, centralized web-based mobile application that ensured allocation concealment. Each study site was assigned a separate account within the mobile app to ensure data security and integrity (details in the Supplements). Participants were blinded but healthcare workers were not, hence the term “open-label”. Subsequent clinical pathways (i.e., confirmatory diagnostics and treatment) were the same for all participants in both study arms.

### Procedures

Individuals presenting at a study site with symptoms suggestive of pulmonary TB were informed about the study and invited to participate. Eligible participants providing written informed consent were enrolled and administered a structured study questionnaire^13^ using Research Electronic Data Capture (REDCap).^14^ Randomization was conducted upon completion of the questionnaire. All participants provided sputum samples for routine TB testing according to country-specific programmatic testing guidelines and venous blood samples for SeroSelectTB assay quality assurance and future development; a schedule of study events is provided in Supplementary Table S2.

Participants randomized to the intervention arm provided 20 ul of finger-prick whole blood, collected with the provided sample transfer device, for immediate on-site SeroSelectTB testing. All intervention participants received their SeroSelectTB test results and were referred into standard health services provided by the clinic.

Health system factors varied substantially between and within countries, and thus the study represents a “real-world” setting. All participants received routine services for TB diagnosis and treatment when indicated. After completing all study-specific activities, participants returned to the clinic for standard services per site-specific protocols. In Ethiopia, participants were referred from the health post to the health center for sputum collection and confirmatory testing. In Tanzania, sputum was collected by the primary healthcare workers and thereafter transported for Xpert testing at a referral lab. In South Africa, sputum was collected by a clinic nurse and submitted to the National Health Laboratory Service (NHLS) for testing. Participants provided at least two sputum samples in South Africa (≥1 ml each); one sputum for Xpert MTB/RIF Ultra (Ultra; version 4, Cepheid, Sunnyvale, USA),^15^ and one for decontamination with 1% NaOH-NALC prior to Mycobacteria Growth Indicator Tube (MGIT) 960 culture (MGIT960; Becton Dickinson Diagnostic Systems, Sparks, USA).^16^ A third sputum was collected for MGIT960 culture (if HIV positive or repeat testing if culture was contaminated). All MGIT960 culture testing was conducted at NHLS irrespective of study arm. Xpert Ultra testing in the SOC arm was done at NHLS. In the intervention arm, confirmatory Xpert Ultra testing was performed at the study site for participants who tested SeroSelectTB positive (either T1- and/or T2-bands), I-band positive, or negative SeroSelectTB results with severe symptoms (including HIV positive). If SeroSelectTB was negative, the participant was referred to the clinic for programmatic testing. Sputum induction (with 5% hypertonic saline solution) was available in South Africa for participants who could not expectorate.

Sputum samples were subjected to AFB microscopy (Ethiopia, South Africa, and Tanzania), MGIT960 culture (South Africa) and/or Xpert testing (Ethiopia, South Africa, and Tanzania). Treatment was initiated for participants with positive test results (AFB smear microscopy and/or Xpert) following established national guidelines.

### Outcomes

The primary endpoint was defined as time-to-treatment initiation among participants with bacteriologically confirmed pulmonary TB disease (confirmed by AFB smear microscopy or Xpert) measured in days from enrollment at the primary healthcare facility until the start of TB treatment. Prespecified secondary endpoints included: 1) time to routine TB testing measured as time from enrollment (reporting to the primary healthcare facility) until the tests were performed (AFB microscopy and/or Xpert results), and 2) time to confirmatory Xpert test performed (measured as time from enrollment at the healthcare facility until confirmatory diagnostic Xpert test results were reported). In Tanzania, the date when Xpert was performed was missing for nearly 30% of participants. For these participants, we used the date when a test result was received at the site to impute the number of days from recruitment to testing (See Supplementary materials for details.) An exploratory analysis was conducted to estimate the diagnostic accuracy of the SeroSelectTB test, including sensitivity and specificity, among a subset of participants enrolled at the Cape Town study sites. Diagnostic accuracy was calculated using bacteriological confirmation by Xpert and/or culture as the reference standard (details provided in the Supplementary Appendix).

### Statistical analysis

All analyses were performed on an intention-to-treat (ITT) basis. For the primary endpoint Cox regression models were used to compare two study arms. Participants were followed from time of enrollment at the first level of healthcare to initiation of TB treatment or for 365 days, whichever occurred first. In the overall analysis of all three countries, a shared frailty model,^17^^,^^18^ which included a country-level random effect (frailty), was used. Since participants from the same country are assumed to have the same frailty, the model accounts for between-country heterogeneity and within-country correlations. In addition, the analyses were performed separately for each country with standard Cox proportional hazards models. To evaluate whether the within-group correlation differed from 0, the shared frailty model was compared to the standard Cox model with a likelihood ratio test. All analyses were adjusted for sex and age. Time to the primary and secondary endpoints in the two groups were represented graphically with Kaplan–Meier curves and compared with a log-rank test. All analyses were performed using STATA 18 (©StataCorp LLC).^19^

### Role of the funding source

The funder of the study had no role in the study design, data collection, data analysis, data interpretation, or writing of the manuscript. The corresponding and last authors had full access to the study data and had the responsibility to submit for publication on behalf of the coauthors.

## Results

### Study population

**Fig. 1:**
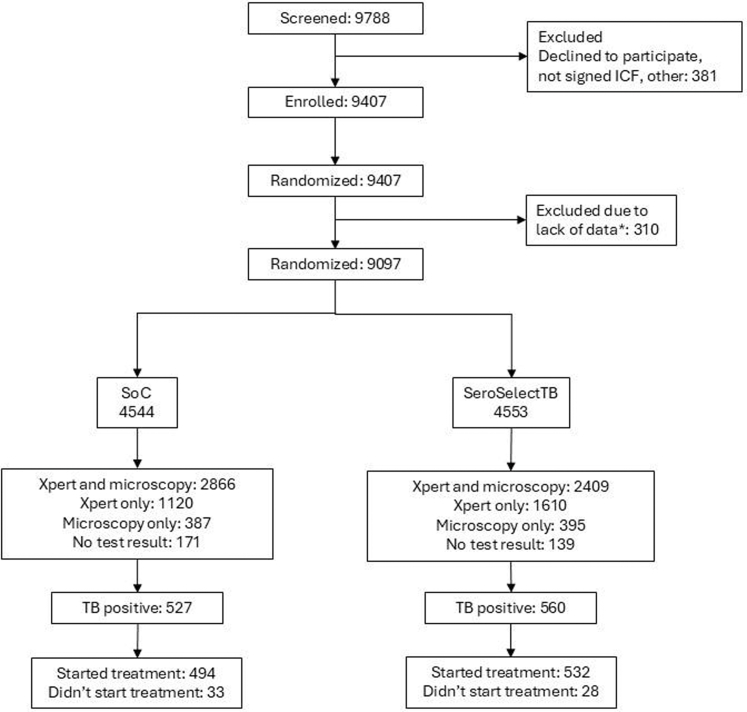
Trial profile. ICF = informed consent form, SOC = standard-of-care arm, TB = tuberculosis. ∗Participants enrolled from the northern part of Ethiopia were excluded from the analysis (n = 310) due to civil unrest in the country and resulting inability to retrieve the data.

Between September 21, 2021, and June 19, 2025, 9097 presumptive pulmonary TB patients were randomly assigned to the SOC (n = 4545) and intervention (n = 4552) study arms (Fig. 1 and Supplementary Fig. S3). Pulmonary TB was confirmed in 1087 (11.5%) participants (SOC = 527; intervention = 560). Participant randomization provided balanced study arms across all sites in terms of age, sex, education, HIV status, number of confirmed TB cases, and duration of TB symptoms (Table 1). The participants’ HIV status was known in South Africa (HIV positive: 43.8% SOC vs. 41.6% intervention) and Tanzania (HIV positive: 14.4% SOC vs. 15.6% intervention). In Ethiopia, no participants living with HIV were enrolled (HIV status was verified from the national registry or self-reported by participants). More than 80% of all participants in both study arms experienced cough for 2 weeks or more before seeking medical care (Table 1).

**Table 1:** Baseline participant characteristics.

	All participants		Ethiopia		South Africa		Tanzania	
	SOC	SeroSelectTB	SOC	SeroSelectTB	SOC	SeroSelectTB	SOC	SeroSelectTB
Socio-demographic								
Age (years), median (IQR)	41.4 (30.6–54.2)	41.2 (30.5–53.9)	35.0 (27.0–45.0)	35.0 (27.0–45.0)	39.0 (30.4–48.6)	38.6 (29.6–48.2)	47.7 (34.2–60.6)	47.8 (34.4–61)
BMI (kg/m2), median (IQR)	21.0 (18.6–24.9)	21.1 (18.6–24.9)	18.2 (16.9–19.6)	18.3 (16.9–19.8)	21.8 (19.1–26.2)	21.7 (19.0–26.5)	22.1 (19.1–25.7)	22.3 (19.8–25.7)
Sex/Male % (N)	48.4 (2201)	48.6 (2213)	35.7 (322)	37.0 (332)	48.2 (709)	48.5 (707)	53.9 (1170)	53.4 (1174)
Sex/Female % (N)	51.5 (2342)	51.4 (2339)	64.3 (580)	63.0 (565)	51.8 (761)	51.5 (751)	46.1 (1001)	46.6 (1023)
Tobacco smoker (current) % (N)	3.3 (149)	3.2 (147)	0.0 (0)	0.0 (0)	9.7 (142)	9.4 (137)	0.3 (7)	0.5 (10)
Clinical								
HIV positive % (N)	21.0 (956)	20.9 (950)	0.0 (0)	0.0 (0)	43.8 (644)	41.6 (607)	14.4 (312)	15.6 (343)
HIV negative % (N)	74.0 (3362)	73.9 (3363)	98.3 (887)	97.2 (872)	54.6 (802)	56.1 (819)	77.0 (1673)	76.1 (1672)
CD4 count (cells/μl), median (IQR)	220 (96–415)	272 (129–442)	N/A	N/A	206 (93–385)	221 (123–395)	375 (201–598)	469 (312–575)
ART use—Yes % (N)	15.1 (687)	15.4 (703)	0.0 (0)	0.0 (0)	28.2 (414)	27.7 (404)	12.6 (273)	13.6 (299)
ART use—No % (N)	5.9 (268)	5.4 (245)	0.0 (0)	0.2 (2)	15.6 (229)	13.9 (202)	1.8 (39)	1.9 (41)
Previous TB completed % (N)	6.2 (281)	6.1 (276)	0.3 (3)	0.5 (4)	15.8 (232)	15.8 (231)	2.1 (46)	1.9 (41)
Symptoms								
Current cough % (N)	96.9 (4404)	97.5 (4437)	95.6 (862)	97.4 (874)	96.8 (1423)	96.8 (1412)	97.6 (2119)	97.9 (2151)
≥2 weeks % (N)	84.0 (3815)	83.6 (3808)	90.7 (818)	92.3 (828)	83.3 (1225)	82.3 (1200)	81.6 (1772)	81.0 (1780)
Fever—Yes % (N)	32.8 (1491)	33.5 (1526)	60.9 (549)	61.8 (554)	27.3 (401)	27.8 (405)	24.9 (541)	25.8 (567)
Fever—No % (N)	67.0 (3044)	66.2 (3012)	38.9 (351)	37.8 (339)	72.7 (1068)	72.2 (1053)	74.8 (1625)	73.7 (1620)
Weight loss—Yes % (N)	65.8 (2988)	66.8 (3039)	78.2 (705)	77.0 (691)	63.7 (936)	65.1 (950)	62.0 (1347)	63.6 (1398)
Weight loss—No % (N)	34.0 (1543)	32.9 (1498)	21.4 (193)	22.5 (202)	36.3 (533)	34.8 (507)	37.6 (817)	35.9 (789)
Night sweats—Yes % (N)	78.4 (3564)	78.3 (3566)	86.3 (778)	85.8 (770)	76.7 (1128)	74.5 (1087)	76.3 (1658)	77.8 (1709)
Night sweats—No % (N)	21.1 (960)	21.4 (976)	13.5 (122)	13.6 (122)	23.1 (340)	25.4 (370)	22.9 (498)	22.0 (484)
Chest pain—Yes % (N)	66.7 (3029)	67.5 (3075)	26.8 (242)	26.2 (235)	69.0 (1014)	68.1 (994)	81.6 (1773)	84.0 (1846)
Chest pain—No % (N)	32.9 (1496)	32.0 (1455)	72.5 (654)	73.4 (658)	30.9 (454)	31.6 (461)	17.9 (388)	15.3 (336)

SOC = standard-of-care arm, IQR = interquartile range, BMI = body mass index, TB = tuberculosis, ART = antiretroviral therapy.

The proportion of Xpert TB positive patients (SOC vs. intervention) who did not start treatment was 8.1% vs. 7.3% in South Africa and 6.4% vs. 5.2% in Tanzania (Supplementary Fig. S3). In Ethiopia, 100% of participants with confirmed TB started treatment irrespective of study arm.

### Estimating overall healthcare system delay

**Fig. 2:**
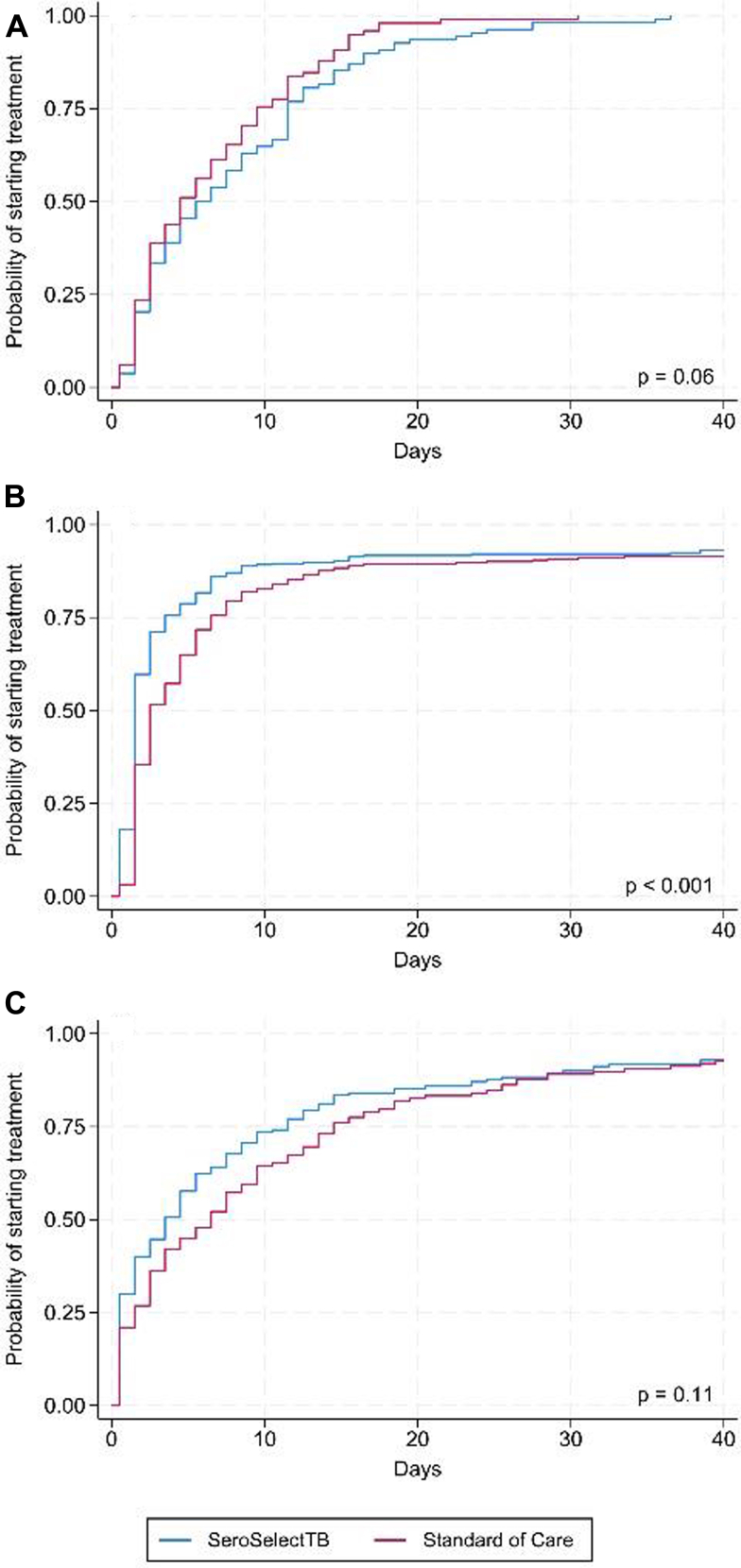
Healthcare system delay: Kaplan–Meier failure curves of time from enrollment until initiation of treatment among confirmed TB patients in A) Ethiopia, B) South Africa, and C) Tanzania.

Fig. 2 shows the Kaplan–Meier failure curves of the time from enrollment until treatment was initiated among confirmed TB patients (plots of −ln(−ln(survival)) against ln(time) for each study arm did not reveal violations of the proportional hazard assumption). Intervention arm patients were more likely to start treatment earlier compared to SOC in South Africa (p < 0.001) and Tanzania (p = 0.11), while the opposite was observed in Ethiopia (p = 0.06). The proportion of patients that started treatment in the intervention arm by day 10 was 70%, 86%, and 69% in Ethiopia, South Africa and Tanzania, respectively. By day 20, more than 90% of patients in Ethiopia and South Africa and 84% of patients in Tanzania had started treatment.

Among all individuals initiating treatment, the median time-to-treatment was 3 days in the SOC arm and 2 days in the intervention arm. In Ethiopia, median time-to-treatment was shorter in the SOC arm compared to the intervention arm (4 days vs. 5 days). However, the opposite was observed in South Africa (2 days vs. 1 day) and Tanzania (5 days vs. 3 days).

The SeroSelectTB test was associated with a more rapid time-to-treatment initiation (measured from enrollment) compared to SOC patients (crude hazard ratio (HR): 1.20; 95% CI: 1.06–1.35) (Table 2). Country-specific results indicated healthcare systems differences (with a significant frailty effect (p = 0.006.), i.e., the within-group correlation differed significantly from 0). In Ethiopia, being in the intervention arm was associated with a higher probability of treatment delay compared to SOC, however the estimated HR was not statistically significant (crude HR: 0.78; 95% CI: 0.59–1.03). The opposite was observed in South Africa and Tanzania, where the crude hazard ratios were 1.36 (95% CI: 1.15–1.62), and 1.19 (95% CI: 0.95–1.50), respectively. Adjusting the regression analysis for sex and age did not change any of the effect estimates. When defining the start of follow-up as the date of testing (Xpert or AFB microscopy) instead of date of enrollment, similar results were observed (Supplementary Table S3).

**Table 2:** Healthcare system delay—from enrollment to treatment initiation.

	Overall		Ethiopia		South Africa		Tanzania	
	SOC n = 519	SeroSelectTB n = 556	SOC n = 98	SeroSelectTB n = 108	SOC n = 283	SeroSelectTB n = 278	SOC n = 138	SeroSelectTB n = 170
TB positive participants initiating treatment, n (%)	486 (93.6)	528 (94.9)	98 (100.0)	108 (100.0)	259 (91.5)	259 (93.2)	129 (93.5)	161 (94.7)
Crude HR (95% CI)	1 (Ref)	1.20 (1.06, 1.35)	1 (Ref)	0.78 (0.59, 1.03)	1 (Ref)	1.36 (1.15, 1.62)	1 (Ref)	1.19 (0.95, 1.50)
Adjusted HR (95% CI)	1 (Ref)	1.20 (1.06, 1.36)	1 (Ref)	0.79 (0.60, 1.04)	1 (Ref)	1.36 (1.14, 1.62)	1 (Ref)	1.19 (0.94, 1.50)

SOC = standard-of-care arm, HR = hazard ratio, 95% CI = 95% confidence interval. Participants were considered positive for TB if confirmed by routine TB testing or Xpert confirmatory testing depending on the setting (for more details refer to Statistical Analysis Plan). Participants were excluded with missing information on sex (n = 0) or age (n = 8); missing date of Xpert Ultra test and sputum smear test (n = 1); date of treatment start prior to date of recruitment (n = 1); time-to-treatment was 365 days or more after date of testing (n = 3).

### Estimating diagnostic delay from enrollment until routine testing and confirmatory TB diagnosis

Time from enrollment to routine TB testing (defined as performance of AFB smear microscopy and/or Xpert according to national programmatic algorithms) was short overall but varied by country (Table 3). In Ethiopia, where AFB smear microscopy was performed for all participants in both study arms, the median time from enrollment to smear testing was 0 days (IQR 0–1) in both arms. In South Africa, AFB smear microscopy was performed in 96·7% of participants in the SOC arm and 65·3% in the intervention arm, with a median time to testing of 1 day (IQR 0–2) in both arms. In Tanzania, AFB smear microscopy was performed in 42·8% of SOC participants and 43·5% of intervention participants, with a median time to testing of 1 day (IQR 0–2) in both arms. Across countries, there were no meaningful differences between study arms in the median time from enrollment to initial routine TB testing.

**Table 3:** Outcomes—Testing coverage and results.

	Overall		Ethiopia		South Africa		Tanzania	
	SOC N = 4544	SeroSelectTB N = 4553	SOC N = 902	SeroSelectTB N = 897	SOC N = 1470	SeroSelectTB N = 1459	SOC N = 2172	SeroSelectTB N = 2197
AFB smear microscopy performed, n (%)								
Yes	3253 (71.6)	2804 (61.6)	902 (100.0)	897 (100.0)	1421 (96.7)	952 (65.3)	930 (42.8)	955 (43.5)
No	1291 (28.4)	1749 (38.4)	0 (0.0)	(0.0)	49 (3.3)	507 (34.7)	1242 (57.2)	1242 (56.5)
Days from recruitment to test, median (IQR)	1 (0–2)	1 (0–2)	0 (0–1)	0 (0–1)	1 (0–2)	1 (0–2)	1 (0–2)	1 (0–2)
Xpert/Ultra Test performed, n (%)								
Yes	3986 (87.7)	4019 (88.3)	606 (67.2)	584 (65.1)	1464 (99.6)	1452 (99.5)	1916 (88.2)	1983 (90.3)
No	558 (12.3)	534 (11.7)	296 (32.8)	313 (34.9)	6 (0.4)	7 (0.5)	256 (11.8)	214 (9.7)
Days from recruitment to test, median (IQR)	2 (0–6)	2 (0–6)	8 (4–13)	8 (4–11)	0 (0–0)	0 (0–0)	4 (2–7)	4 (2–7)
SeroSelectTB Test performed, n (%)								
Yes	0 (0.0)	4552 (99.98)	0 (0.0)	896 (99.9)	0 (0.0)	1459 (100.0)	0 (0.0)	2197 (100.0)
No	4544 (100)	1 (0.02)	902 (100)	1 (0.1)	1470 (100)	0 (0.0)	2172 (100)	0 (0.0)
Days from recruitment to test, median (IQR)	N.A.	0 (0–0)	N.A.	0 (0–0)	N.A.	0 (0–0)	N.A.	0 (0–0)

SOC = standard-of-care arm, N.A. = not applicable, AFB = acid fast bacilli. In Ethiopia and South Africa, Xpert test performed was considered performed if date when Xpert testing was performed was non-missing. In Tanzania, test performed was considered performed if date when Xpert testing was performed was non-missing or date when result of Xpert test was received at site was non-missing.

Time from enrollment to confirmatory Xpert testing varied substantially by country and reflected differences in referral and laboratory organization (Table 3). In Ethiopia, where Xpert testing was conducted at centralized laboratories, the median time to Xpert testing was 8 days in both the SOC arm (IQR 4–13) and intervention arm (IQR 4–11). In Tanzania, the median time to Xpert testing at centralized laboratories was 4 days (IQR 2–7) in both study arms. In South Africa, where on-site testing was available at study clinics, the recorded median time to Xpert testing was 0 days (IQR 0–0) in both arms. However, for programmatic samples processed at referral laboratories in South Africa, the date of result receipt at the clinic was not consistently recorded; therefore, the interval between laboratory processing and clinical receipt of results could not be quantified. Overall, no consistent differences between study arms were observed in time to confirmatory Xpert testing within countries.

### SeroSelectTB test diagnostic accuracy

Diagnostic test accuracy was estimated using a randomly selected subset of samples obtained from participants at the study sites in Cape Town. The SeroSelectTB test sensitivity was 75.9%, the specificity was 49.5% in a high HIV setting, whereas sensitivity and specificity were 68.8% and 76.0% among HIV negative participants, respectively (details in the Supplements).

### Feasibility

In 2021, health extension workers in Ethiopia (n = 21), healthcare workers in Tanzania (n = 21), and research nurses in South Africa (n = 6) initially received up to 2 h of practical, hands-on training on performing the SeroSelectTB test. This training was followed by close supervision at their respective sites and refresher courses to ensure correct implementation. To support routine use, quick reference guides and visual band intensity indicators were provided at each site. As a result, all healthcare workers reported confidence in performing the test according to the instructions (details in the Supplements).

## Discussion

In this multicenter, open-label, parallel-group, randomized controlled trial conducted in Ethiopia, South Africa, and Tanzania, integration of the SeroSelectTB rapid point-of-care triage test into routine diagnostic pathways was associated with a shorter time-to-treatment initiation among bacteriologically confirmed pulmonary TB cases compared with SOC (adjusted hazard ratio 1.20, 95% CI 1.06–1.36). For prespecified secondary outcomes, no consistent differences between study arms were observed in time from enrollment to routine TB testing or in time to confirmatory Xpert testing within countries, likely reflecting similarities in downstream diagnostic processes once patients entered programmatic care. In an exploratory analysis conducted in Cape Town, the SeroSelectTB test demonstrated moderate sensitivity that exceeded the WHO minimum sensitivity target for a non-sputum triage test; however, specificity was lower and varied by HIV status. Together, these findings suggest that use of a rapid non-sputum triage test at the point of first presentation can modestly accelerate treatment initiation without disrupting existing diagnostic workflows, supporting further evaluation of triage-based diagnostic strategies within a diagnostic escalation framework in high-burden settings.

Although the absolute reduction in median time-to-treatment initiation was modest (1–3 days across settings), even short delays in initiating TB treatment can have clinical and public health consequences.^20^ Earlier treatment reduces the period of infectiousness and may limit household and community transmission, as emphasized in the WHO End TB Strategy.^2^ The 20% relative increase in the rate of treatment initiation reflects acceleration within existing care pathways rather than changes to downstream laboratory processes, which were similar between study arms. Heterogeneity across countries was observed: earlier treatment initiation was seen in South Africa and Tanzania, whereas no statistically significant benefit was detected in Ethiopia. Variation in national diagnostic algorithms, centralization of molecular testing, and programmatic changes during trial implementation most likely attenuated between-arm differences in some settings, suggesting that the impact of triage-based strategies depends on local health system organization and baseline diagnostic delays.

In an exploratory analysis conducted with samples obtained from the Cape Town study sites, SeroSelectTB demonstrated a sensitivity of 75.9% in a high HIV-prevalence setting, exceeding the WHO minimum sensitivity target (≥65%) for a non-sputum-based triage test.^3^ Specificity was lower and varied by HIV status. These findings support the intended role of SeroSelectTB as a triage tool within a diagnostic escalation strategy, whereby individuals at increased risk of tuberculosis are prioritized for confirmatory molecular testing rather than diagnosed on the basis of serology alone. In this context, moderate sensitivity may be sufficient to accelerate downstream testing and treatment initiation when integrated into existing care pathways.

Implementation of the SeroSelectTB test was feasible across diverse primary healthcare settings. Health extension workers, nurses, and clinic staff were trained through brief, hands-on sessions and reported confidence in performing and interpreting the assay. The test required only a finger-prick blood sample, produced results within minutes, and did not require laboratory infrastructure, electricity, or cold-chain storage. Integration of the test into routine workflows did not disrupt standard diagnostic pathways, and confirmatory molecular testing procedures remained unchanged. These findings indicate that a rapid serological triage test can be operationalized at the point of first contact and incorporated within a structured diagnostic escalation strategy, whereby individuals at increased risk are prioritized for confirmatory testing within decentralized TB diagnostic programs.

Our study has several limitations. Changes in national TB control program guidelines affected our study in several ways. Xpert was introduced as the standard confirmatory test in all participating countries, and patient management routines changed. Instead of referring presumptive TB patients to next level of healthcare and/or performing AFB microscopy on site, sputum samples were collected and sent to a centralized laboratory for Xpert testing in Tanzania. As a result, AFB microscopy was not performed for 50% of participants. According to national guidelines in Ethiopia and Tanzania, AFB microcopy should be performed and treatment initiated for patients with positive smear results. Sputum is thereafter subjected to Xpert confirmatory testing and treatment regimens revised when warranted. These guidelines were followed in Ethiopia, and sporadically in Tanzania. Our sites in South Africa were healthcare clinics with established research facilities equipped with sophisticated laboratories in which all TB testing methods were available, representing the ideal situation. Worldwide, only 52% of individuals presenting with presumptive TB were tested with WHO-approved molecular diagnostics (Xpert MTB/RIF or Truenat) for their initial diagnosis in 2024. However, in many low-income countries, particularly in Africa and parts of Asia, this percentage is below 40%.^2^

In conclusion, integration of a rapid, non-sputum-based triage test at the point of first presentation was associated with earlier treatment initiation among individuals with bacteriologically confirmed pulmonary tuberculosis in this multi-country randomized trial. Although the absolute reduction in time-to-treatment was modest and varied across settings, the findings demonstrate that triage-based diagnostic strategies can be embedded within routine primary healthcare systems without disrupting established confirmatory pathways. Further optimization of serological platforms, including improvements in specificity and performance among people living with HIV, will be important to maximize programmatic value. Integration of TB triage testing with rapid HIV diagnostics at the point-of-care may offer additional efficiencies in high-burden settings where co-testing is clinically indicated. Beyond facility-based implementation, rapid non-sputum triage tools may have particular utility in decentralized, community-based, and in-home testing models, where they can serve as an initial screening step to identify individuals and households requiring diagnostic escalation. Consistent with emerging evidence supporting household-level screening and structured escalation strategies^21^ such approaches could strengthen completion of diagnostic cascades among high-risk populations, including household contacts. Further implementation studies are warranted to define optimal integration models and to assess effects on transmission, patient outcomes, and health system efficiency.

## Contributors

CHH, MS, and SAY conceptualized the study. CHH, MS, SAY, IL, KB, BMN, and GT were responsible for methodology and study design. AJ, SN, JD, and JA were responsible for software and development of the randomization app and automatic QC reporting system. AJ was responsible for project website (www.seroselecttb.org) development and maintenance. NB, JDP, TW, and SG were responsible for the SeroSelectTB assay manufacturing. KB, TA, MY, MW, YAH, SAYE, BMN, DCK, HHS, JB, FM, GAO, AJN, GT, AOO, WVN, and AMM conducted the field investigations. AJ, MS, SN, JD, and JA were responsible for data curation. KB, TA, BMN, JB, FM, AOO, WVN, and AMM were responsible for data validation and/or interpretation. IL, MS, AJ, SD, JD, and JA conducted the formal data analysis and visualization. KB, GT, BMN, MS, and CHH supervised the study. KB, BMN, GT, NB, JDP, TW, SG, and CHH were responsible for local financial resources, staff resources, and laboratory equipment. CHH was responsible for funding acquisition and project administration. MS, AJ, and CHH wrote the original draft manuscript. All authors reviewed, edited, and approved the final version of the manuscript. AJ, MS, and CHH accessed and verified all data in the study and had final responsibility for the decision to submit for publication.

## Data sharing statement

The study protocol and statistical analysis plan can be downloaded from the Norwegian Research Information Repository (https://nva.sikt.no) at the Norwegian Institute of Public Health. Country-specific anonymized participant data will be made available upon request to principal investigators (GT: gtheron@sun.ac.za; KB: kidist.bobosha@ahri.gov.et; BMN: balthazar.nyombi@kcmcu.ac.tz) at the respective partner institutions (Stellenbosch University, Armauer Hansen Research Institute, KCMC University). Access will be granted following approval from the IRBs at partner institutions and signed data use agreement form.

## Declaration of interests

MS is employed at GSK and holds financial equities in GSK. NB and JDP are employed at Lateral Flow Laboratories (Pty) Ltd, and TW is employed at InVivo BioTech Services GmbH, and have future commercial interests in the SeroSelectTB rapid assay. SG was employed at InVivo BioTech Services GmbH until June 2025. Project funds covered travel to consortium meetings and field work for all coauthors except ID and AMM, and travel only to consortium meetings for SG and TW. The authors declare no other competing interests.

## Appendix A Supplementary data

Multicenter supplement 140326 FINAL CLEAN

Protocol_EDCTP_DM-#116831_SeroSelectTB

Equitable_Partnership_Declaration_Form_CHH

CE-Booklet_SeroSelectTB_English (electronic)

CE-MythsFacts_SeroSelectTB_English (electronic)

mSeroSelectTB SAP v05_280225

LD3366 SEROSELECTTB PACK INSERT FINAL rev 008_6 June 2024

ICF SeroSelectTB_REK_template for translation_140920
