# Supplementary material for: Feasibility, accuracy, and effect of a rapid point-of-care serological test (SeroSelectTB) to identify presumptive pulmonary TB patients for confirmatory testing in Ethiopia, South Africa, and Tanzania: a multicenter, open-label, parallel-group, randomized, controlled trial
Source: eClinicalMedicine. 2026 Apr 25;95:103914. doi: 10.1016/j.eclinm.2026.103914 (PMC13129460; doi:10.1016/j.eclinm.2026.103914)
Supplement: Multicenter supplement 140326 FINAL CLEAN [file mmc1.docx]

# Supplemental Information

Table of Contents

[Supplemental Information 1](#_Toc214443693)

[Study site descriptions 1](#_Toc214443694)

[Ethical approvals 5](#_Toc214443695)

[Community Engagement 5](#_Toc214443696)

[Methods 5](#_Toc214443697)

[Randomization 6](#_Toc214443698)

[Sample size 7](#_Toc214443699)

[Primary objective and endpoints 7](#_Toc214443700)

[Feasibility assessment 8](#_Toc214443701)

[SeroSelectTB test development 9](#_Toc214443702)

[SeroSelectTB test accuracy 10](#_Toc214443703)

[Results 10](#_Toc214443704)

[Estimating healthcare system delay from positive TB test until initiation of treatment 11](#_Toc214443705)

[Feasibility 12](#_Toc214443706)

[SeroSelectTB test accuracy 12](#_Toc214443707)

[References 14](#_Toc214443708)

## Study site descriptions

Dale and Aleta Chuko Districts (Sidama Region, Ethiopia)

Northern Health District of Cape Town (South Africa)

Eastern Health District of Cape Town (South Africa)

Moshi Municipal, Moshi, and Hai Districts (Tanzania)

#### Table S1. Study sites details.

| **Site / Region** | **Location & Socioeconomics** | **HIV & TB Burden** | **Health Services / TB Program** |
| --- | --- | --- | --- |
| 21 sites (health posts) in Dale and Aleta Chuko Districts, Sidama Region, Ethiopia | Sidama Region is in the south-central part of Ethiopia, and is a home to approximately five million people. The region’s economy is heavily agricultural. Crop production is common for household consumption. The region is also known for coffee production that plays a major role in income generation. Dale and Aleta Chuko districts are 300 to 380 km from the capital city, Addis Ababa.  The initial study sites were five districts in the West Gojam Zone, from which a total of 318 participants were enrolled between October 2022 and July 2023. However, intermittent civil unrest in the zone disrupted the timely implementation of the project, data collection and patient follow-up, which resulted in study termination at these sites. Data collected from the 310 participants could not be fully completed nor adequately verified, therefore were not included in the main analysis. | The Sidama Region is identified as a severely TB affected area by the Ministry of Health (MOH). The estimated prevalence of TB in the region is 229/100,000, significantly above the national prevalence of 146/100,000.  HIV prevalence is low in the region particularly in the districts, approximately 0.4%, while prevalence in Hawassa city, the capital and center for tourism ranges from 1.6 to 2.4%. | In Ethiopia, the healthcare system is organized into three tiers. The primary healthcare system consists of three levels. Health posts are the first level, providing basic services through female health extension workers to about 5,000 people. These facilities are not equipped with laboratories. The second level is the health center, which serves 25,000–40,000 people. Health centers are relatively better equipped, staffed with trained nurses, health officers, and laboratory personnel, and provide a range of decentralized services. The third level comprises primary hospitals, which serve 1–1.5 million people and commonly function as Xpert sites for TB confirmatory testing.  The secondary and tertiary levels of the healthcare system are general hospitals and specialized hospitals, respectively.  The SeroSelectTB project was implemented within the primary healthcare system. Participant enrollment and SeroSelectTB testing were conducted at the health posts, while sample collection and smear microscopy were performed at the health centers. Sputum samples were then sent to primary hospitals for Xpert testing. All regional health bureaus abide by and implement national guidelines.  SOC procedures are conducted in accordance with national TB control program guidelines. Presumptive TB patients are evaluated based on clinical symptoms. AFB smear microscopy is performed on site if indicated and available, and TB treatment is initiated when smear positive results are obtained. Sputum samples are subjected to Xpert confirmatory testing, and treatment regimens revised if warranted. For sites without AFB microscopy facilities, sputum samples are sent to facilities equipped for Xpert confirmatory testing. |
| Bloekombos, Wallacedene, Scottsdene, Kraaifontein Community Health Centre, Cape Town, South Africa | Located in the northern suburbs of Cape Town, within the Kraaifontein area, serve underserved peri-urban communities with socioeconomic vulnerabilities. | High TB rates (historically >300/100,000) and significant TB/HIV coinfection, especially among youth. | Primary health care (PHC) facilities delivering TB services under South Africa’s national TB program managed by the National Department of Health. District Health Services structure ensures clinics, and community health centers provide comprehensive primary care, outreach, and supervision.  National TB program: TB screening, Xpert testing for diagnosis, specific management protocol for TB/HIV co-infection, and DOTs.  SOC procedures are conducted in accordance with national TB control program guidelines. Presumptive TB patients are evaluated based on clinical symptoms. AFB smear microscopy is performed on site if indicated and available, and TB treatment is initiated when smear positive results are obtained. Sputum samples are subjected to Xpert confirmatory testing, and treatment regimens revised if warranted. For sites without AFB microscopy facilities, sputum samples are sent to facilities equipped for Xpert confirmatory testing. |
| Delft Clinic, Cape Town, South Africa | Tygerberg Eastern Health District of Cape Town |  | PHC clinic offers community healthcare services including child, women and men’s healthcare and general TB, HIV and STI care.  SOC procedures are conducted in accordance with national TB control program guidelines. Presumptive TB patients are evaluated based on clinical symptoms. AFB smear microscopy is performed on site if indicated and available, and TB treatment is initiated when smear positive results are obtained. Sputum samples are subjected to Xpert confirmatory testing, and treatment regimens revised if warranted. For sites without AFB microscopy facilities, sputum samples are sent to facilities equipped for Xpert confirmatory testing. |
| Nontyatyambo CHC, East London, South Africa | Buffalo City Metro, Eastern Cape, serve under-resourced, rural–peri-urban populations. | High regional TB incidence (TB notification rate between 900-999 cases per 100,000 population), and HIV prevalence. Eastern Cape had an estimated HIV prevalence of 12.7% (95% CI: 12.2%-13.3%), and an estimated 18.3% (95% CI: 17.5%-19.2%) HIV prevalence among people aged 15-49 years). | PHC TB & HIV services: referral networks to specialized TB hospitals (Fort Grey, Nkqubela) for advanced diagnostics, ARVs, chest X-ray, inpatient care.  SOC procedures are conducted in accordance with national TB control program guidelines. Presumptive TB patients are evaluated based on clinical symptoms. AFB smear microscopy is performed on site if indicated and available, and TB treatment is initiated when smear positive results are obtained. Sputum samples are subjected to Xpert confirmatory testing, and treatment regimens revised if warranted. For sites without AFB microscopy facilities, sputum samples are sent to facilities equipped for Xpert confirmatory testing. |
| 21 sites providing primary health care (dispensaries, health centers, hospitals) in Moshi District Council (rural), Moshi Municipal Council (urban), and Hai Districts, Kilimanjaro Region, northern Tanzania | Kilimanjaro Region comprises seven districts, of which three selected districts with an estimated population of 1.8 million were included in the study. The region’s economy depends on agriculture in rural areas (small-scale farming of coffee, bananas and beans), small- to medium-scale businesses in urban settings, and tourism. | TB case notifications are high in Kilimanjaro Region are high, with 3,352 new and relapse cases reported in 2023.  HIV prevalence in Kilimanjaro region is approximately 4%. Collaborative TB/HIV services are implemented that include HIV screening and counselling for TB patients, and TB screening for people living with HIV. In the 2023, the average rate of new and relapse TB patients with HIV in Kilimanjaro Region was 13.8% as compared to the national rate of 17%. | In 2023 the national TB control program in Tanzania adopted the WHO recommendation for TB diagnosis by sputum smear microscopy and Xpert.  The healthcare system in Tanzania provides services from the community level to national level. Primary care includes basic preventive and curative services provided by community healthcare workers at dispensaries and health centers.  Secondary level services are offered by district and regional referral hospitals with specialized care, where TB confirmatory testing (Xpert Ultra and Truenat) is performed. Specialized zonal and national hospitals offer advanced medical services.  While Kilimanjaro Region provides advanced health and social services as compared to other regions, rural areas face challenges including lack of skilled healthcare workers and inadequate reagent supplies and equipment.  SeroSelectTB study sites included primary healthcare facilities (dispensaries) where sputum samples were collected and sent to secondary level facilities for confirmatory Xpert testing. Some primary healthcare facilities (health centers) screened presumptive TB patients by direct sputum microscopy and thereafter sent AFB-positive sputum samples to secondary level facilities for confirmation by Xpert Ultra.  SOC procedures are conducted in accordance with national TB control program guidelines. Presumptive TB patients are evaluated based on clinical symptoms. AFB smear microscopy is performed on site if indicated and available, and TB treatment is initiated when smear positive results are obtained. Sputum samples are subjected to Xpert confirmatory testing, and treatment regimens revised if warranted. For sites without AFB microscopy facilities, sputum samples are sent to facilities equipped for Xpert confirmatory testing. |

## Ethical approvals

All procedures were conducted in compliance with national and institutional guidelines. The following approvals were secured:

Norwegian Institute of Public Health (NIPH), Oslo Norway:

- REK South East: Regional Committee for Medical Research Ethics South East Norway (national ethical approval), ref # 60638 SeroSelectTB: granted 19 May 2020 through 31 December 2023, and 6 November 2023, extension approval 6 November 2023, through 31 December 2025.
- DPIA (Data Protection Impact Assessment), approval granted 15 December 2020 for duration of project, registered in the NIPH archival system (P360, ref # 17/10773; project data base # 2818).

Stellenbosch University, Cape Town, South Africa:

- HREC: Health Research Ethics Committee (institutional ethical approval), project #15189/ethics ref #M20/06/017, granted 19 October 2020, renewed annually.
- Cape Town City Health (city ethical approval), ref #27975, granted 15 December 2020, renewed annually.

KCMC University, Moshi, Tanzania:

- KURERC: KCMC University Research Ethics Review Committee (institutional ethical approval), certificate #2480, granted 16 September 2021, renewed annually.
- NIMR: National Institute for Medical Research (national ethical approval), certificate #NIMR/HQ/R.8a/Vol.IX/3713, granted 1 July 2021, renewed annually.
- COSTECH: Tanzania Commission for Science and Technology (a parastatal organization responsible for coordinating, promoting, and approving research), ref # 2022-114-NA-2021-293, granted 11 February 2022, renewed annually.
- TMDA: Tanzania Medicines and Medical Devices Authority (clinical trial authorization), certificate #TZ22CT0008, granted 9 June 2022, renewed biannually.

Armauer Hansen Research Institute, Addia Ababa, Ethiopia:

- MoSHE/MoE: Ministry of Science and Technology and Ministry of Education (national ethical approval), ref # 04/246/61/21, granted 29 April 2021, renewed annually.
- AAERC-AHRI/ALERT: AAERC: AHRI/ALERT Ethics Review Committee; AHRI: Armauer Hansen Research Institute; ALERT: All Africa Leprosy, Tuberculosis and Rehabilitation Training Centre Hospital: (institutional ethical approval), ref #PO29/20, granted 3 September 2020, renewed annually.

## Community Engagement

Community Engagement materials were designed using a template kindly provided by the International AIDS Vaccine Initiative (formerly Aeras). Texts for the Myths & Facts flyer and Clinical Trial booklet were professionally translated from English to Afrikaans, isiXhosa, Amharic, and Kiswahili. See attached flyer and booklet in English. Materials are available in all languages at <https://www.seroselecttb.org/resources>.

## Methods

#### Figure S1. Participants’ enrollment diagram.


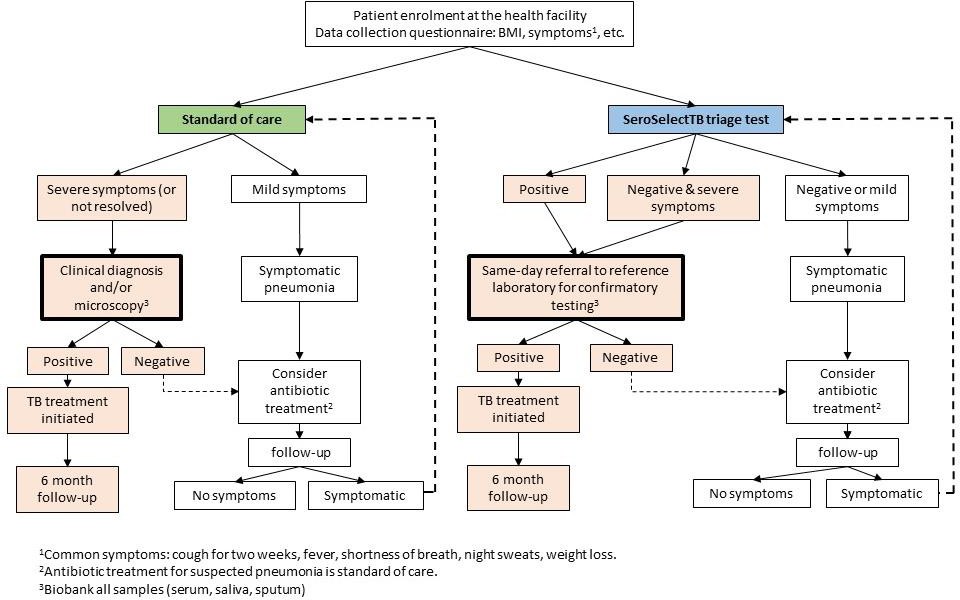


TB = tuberculosis, BMI = body mass index.

### Randomization

The randomization process utilized a block-randomization algorithm implemented through a mobile application, which was specifically designed to operate in resource-constrained environments with limited or no internet access. Each study site was assigned a separate account within the mobile app to ensure data security and integrity. The mobile application featured automatic data synchronization capabilities, allowing healthcare workers or assigned site coordinators to randomize participants offline with local data storage, then automatically synchronize all changes with the central server once internet connectivity was restored, ensuring accurate and secure recording of participant allocation data while maintaining consistency across all study sites ^1^.

A detailed schedule of study events in each country is provided in Table S2.

#### Table S2. Schedule of events.

|  | Ethiopia | South Africa | Tanzania |
| --- | --- | --- | --- |
| **Baseline Evaluation** | | | |
| Informed consent | X | X | X |
| Inclusion/ exclusion criteria verified | X | X | X |
| Locator information | X | X | X |
| Brief medical history | X | X | X |
| Questionnaire | X | X | X |
| HIV status reported | X | X | X |
| **TB and Other Lab Investigations** | | | |
| SeroSelectTB test | X | X | X |
| Sputum Xpert (1) | X | X | X |
| AFB microscopy | X | X | X |
| Blood CD4 count / viral load |  | X | X |
| MGIT960 culture |  | X |  |
| **Further Contact** | | | |
| Verification of TB diagnosis | X | X | X |
| Verification of TB treatment initiation | X | X | X |
| Verification of vital status | X | X | X |

(1) Sputum Xpert MTB/RIF Ultra or Xpert-MTB/RIF whenever the participant has cough, fever, weight loss or night sweats. HIV=human immune deficiency virus. MGIT = Mycobacterial Growth Indicator Tube. TB=tuberculosis.

### Sample size

Assuming an effect size (hazard ratio) of 1.1, the sample size needed to obtain a power of 90% for the log-rank test was 2314 presumptive pulmonary TB patients in each arm of the study in each country. Using a mean diagnostic delay duration of 22 days, a standard deviation of 12 days, and clinically significant 5-day difference in duration of health systems’ delay between the two groups, the calculated sample size was 123 TB cases in each arm of the study in each country (significance level 5% (two-sided), power 90%). Based on prevalence data available prior to study start, it was estimated that 3321, 2829 and 2314 presumptive TB patients should be enrolled in each study arm in Ethiopia, Tanzania and South Africa, respectively.

### Primary objective and endpoints

To evaluate health systems’ diagnostic delay, measured as the time from reporting to the primary healthcare facility to TB treatment initiation among presumptive TB patients in both arms of the trial. Statistical Analysis Plan attached.

To evaluate primary objective, three endpoints were defined as follows:

1. Time to initiation of TB treatment among participants tested positive for TB (either via routine TB testing or Xpert confirmatory testing depending on the setting and scenario), is an endpoint defined as time (in days) from the patient’s enrollment at the first level of healthcare until the start of TB treatment.
2. Time to routine TB test performed is an endpoint measurement, i.e. AFB smear microscopy (scenario A on Figure S2), defined as time (in days) from the patient’s enrollment at the first level of healthcare until routine diagnostic test results were available.
3. Time to confirmatory Xpert test performed is an endpoint measurement defined as time (in days) from the patient’s enrollment at the first level of healthcare until confirmatory diagnostic Xpert results were available. In scenario A, a participant may have both routine and confirmatory tests, and the treatment will start depending on the results; in scenario B only confirmatory Xpert test is available since in certain settings routine microscopy testing is not performed (Figure S2).


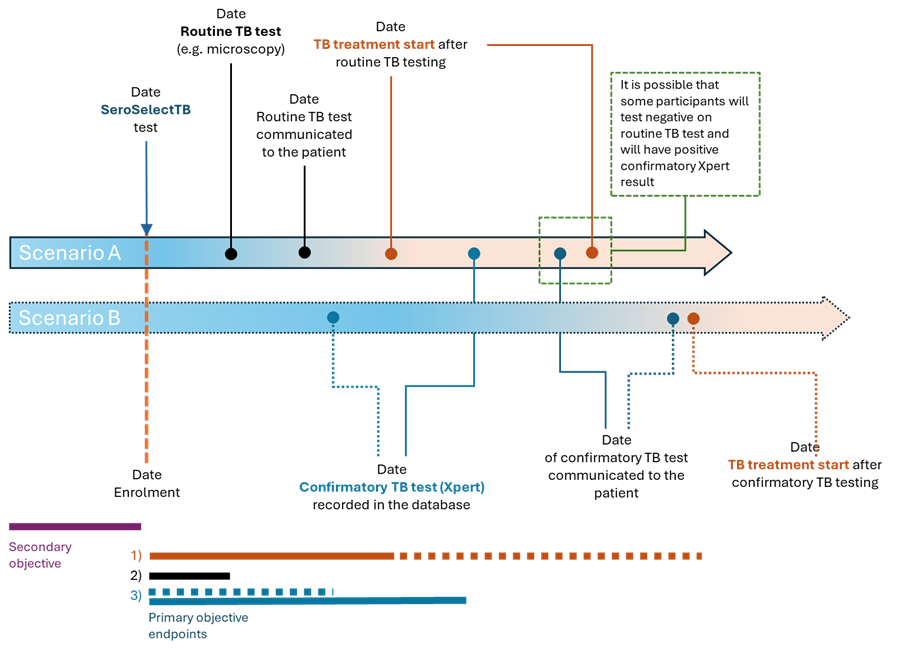


#### Figure S2. Graphical representation of objectives and endpoints with two possible scenarios.

Scenario A (full lines) represents settings where routine AFB microscopy is used (e.g., Ethiopia), scenario B (dashed lines) represents settings where microscopy is most likely not used and is replaced by confirmatory Xpert testing (e.g., South Africa and Tanzania), timeline is not to scale. The date of routine and/or confirmatory Xpert testing results are communicated to the patient should be the same date of TB treatment start. However, the time gaps in the scenario timelines illustrated above appear to be longer. This is for easier readability since the timelines are not to scale in the figure.

### Feasibility assessment

Feasibility assessment of the SeroSelectTB test was conducted to evaluate practicality in field implementation. The assessment focused on the simplicity of performing the test in primary healthcare facilities, the level of training required for primary level health workers, the turnaround time for results, and the extent to which the test could be integrated into the existing health system.

In 2021, health extension workers in Ethiopia (n=21), healthcare workers in Tanzania (n=21), and research nurses in South Africa (n=6) initially received up to two hours of practical, hands-on training on performing the SeroSelectTB test. This training was followed by close supervision at their respective sites and refresher courses to ensure correct implementation. To support routine use, quick reference guides and visual band intensity indicators were provided at each site. As a result, all healthcare workers reported confidence in performing the test according to the instructions.

A mock exercise designed as a refresher course using SeroSelectTB cassettes prepared with 16 different digitally designed test results was conducted to assess healthcare workers’ and nurses’ skills and confidence in reading test bands and interpreting results. Exercises were held during 2023 in Tanzania, Ethiopia, and South Africa in which all healthcare workers (n=57) involved in the trial from all sites participated. The exercise was successful with all participants demonstrating accurate reading, interpretation and documentation of results. In addition, the mock cassettes were used throughout the study in refresher courses and for training new healthcare workers at the sites.

In summary, the 15-minute turnaround time, the simplicity of the procedure, and the non-cold chain requirement of the test make SeroSelectTB particularly well suited for primary level healthcare settings, where staff often have limited laboratory training and the sites have minimal infrastructure. These characteristics strongly support the ease of integrating the test into community-based and resource limited health systems.

### SeroSelectTB test development

Background:

A rapid non-sputum-based triage test for active TB, which identifies symptomatic individuals for confirmatory diagnostic investigation, is a diagnostic priority advocated by the WHO strategy to end TB ^2^. Use of a rapid test at the primary healthcare level can drastically reduce diagnostic delay by expediting referral, confirmatory testing and treatment, thus improving patients’ outcomes and decreasing economic burden on health systems and individuals.

We have developed a rapid serological triage test, SeroSelectTB as an add-on to, and *not* a replacement for, downstream tests including smear microscopy, Xpert Ultra and/or culture in accord with the WHO TB triage test target product profile (TPP) published in 2014 ^3^. SeroSelectTB was developed to conform to the WHO “ASSURED” criteria: **A**ffordable by those at risk, **S**ensitive, **S**pecific, **U**ser-friendly, **R**apid and **R**obust, **E**quipment-free, and **D**elivered to those in need ^4^. SeroSelectTB is a ready-to-use unit and does not require laboratory facilities or highly trained personnel ^5^.

Assay development:

More than 100 *Mycobacterium tuberculosis* (Mtb) genes encoding proteins with diagnostic potential were investigated at the Norwegian Institute of Public Health (NIPH). Selected proteins were cloned and expressed in a eukaryotic system at InVivo BioTech Services GmbH, Hennigsdorf, Germany. DNA constructs encoding the selected Mtb antigens for efficient expression as IgG-fusion proteins via transient production were designed and optimized. This approach facilitates the purification of correctly folded antigens, an essential process for efficient antibody capture.

At NIPH we identified combinations of secreted and membrane-associated antigens involved in cell wall/cell processes and lipid metabolism that differentiate between active disease and latent infection. Our network partners in Africa and Asia collected over 3,500 sera (active TB, symptomatic non-TB, TB/HIV, and healthy endemic community controls) between 2009 and 2011. We selected combinations of Mtb antigens with diagnostic potential for further testing. Compared to previous attempts to develop serological diagnostic tools based on a single Mtb antigen, SeroSelectTB test is innovative in its unique combination of antigens immobilized on one membrane.

By screening these well-characterized samples against combinations of antigens on multiplex and ELISA platforms, sensitivities of 84-95% among culture-confirmed TB cases, and specificities of 90-95% and 97-100% among symptomatic non-TB and healthy endemic controls, respectively, were obtained. Samples from symptomatic people without TB yielded negative results as did samples from the healthy endemic controls. Importantly, spoligotyping and MIRU-VNTR analyses performed at NIPH indicated that antibody reactivity to the selected antigens is not Mtb strain-specific.

SeroSelectTB detects TB in the context of HIV co-infection. In preliminary investigations the selected antigen combination detected TB among people with HIV co-infection with sensitivities of 74-85% when compared to people with TB alone. TB diagnosis is often based on Ziehl Neelsen sputum smear microscopy in resource poor settings. The sensitivity of smear microscopy is approximately 50% among presumptive TB patients and falls to 35% among persons co-infected with HIV.

In 2012 the SeroSelectTB prototype was transferred to Lateral Flow Laboratories (LFL) in South Africa for additional development, modification, and assay production. The selected antigen combination was incorporated into an established immunochromatographic lateral flow platform at LFL.

Description of the device:

The SeroSelectTB assay is designed as a simple, rapid, qualitative, and affordable for detecting the presence of Mycobacterium tuberculosis antibodies *in vitro*.


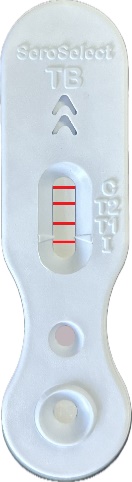
The test strip assembly is housed in a high-quality cassette with the identifier ‘SeroSelect TB’ embossed on the top. Two sample wells, one for whole blood and one for serum, and a window slot to view test results are visible on the front of the device. C, T2, T1, and I are embossed on the right-hand side of the result window to identify resulting bands shown in the window after the test run.

The SeroSelectTB rapid devices are provided in sealed individual foil pouches containing desiccant. A complete test kit carton, identified as SeroSelectTB, contains sufficient materials for 25 tests. This includes 25 sealed and printed foil pouches, 25 disposable lancets, 25 disposable specimen transfer devices, 25 alcohol swabs, one reaction buffer bottle and one Instruction for Use package insert (attached).

The SeroSelectTB test is currently available for research purposes only and is manufactured by Lateral Flow Laboratories (Pty) Ltd, Muizenberg, South Africa.

### SeroSelectTB test accuracy

We retested 454 serum samples randomly selected from study participants in South Africa.

We calculated the proportion of SeroSelectTB and Xpert results that were positive for TB, and the sensitivity and specificity of SeroSelectTB test. A subgroup analysis was performed by HIV status of participants.

## Results

Between September 21, 2021 and June 19, 2025, 9097 presumptive pulmonary TB patients were randomly assigned to the standard-of-care (SOC, n=4545) and intervention (n=4552) study arms, of which 2109 in Ethiopia, 2929 in South Africa, and 4369 in Tanzania (Figure S3).

#### Figure S3. Consort diagram with country specific data.


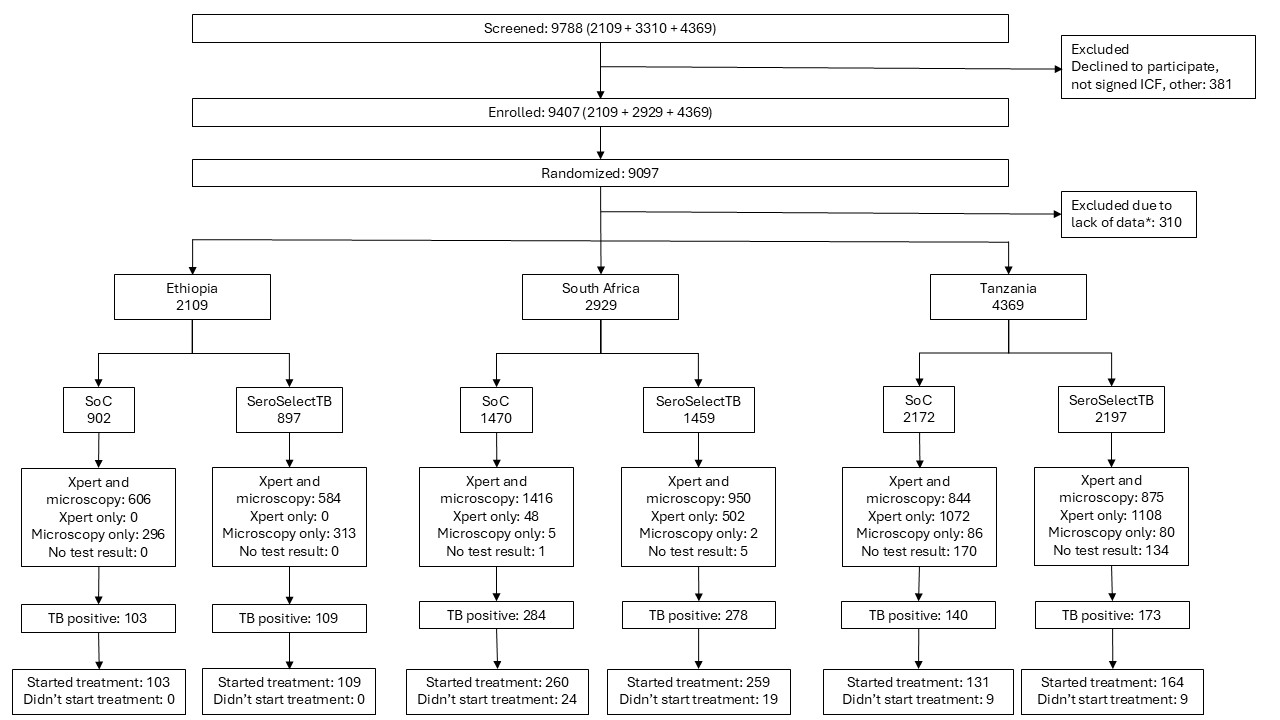


ICF = Informed Consent Form, SOC = standard-of-care, TB = tuberculosis. * Participants enrolled form the northern part of Ethiopia were excluded from the analysis (n=310) due to civil unrest in the country and inability to retrieve the data from the conflict zone.

### Estimating healthcare system delay from positive TB test until initiation of treatment

When defining the start of follow-up as the date of testing (Xpert or AFB sputum smear microscopy) instead of date of enrollment, SeroSelectTB test was associated with a more rapid time-to-treatment initiation compared to SOC patients (crude hazard ratio (HR): 1.14; 95%CI: 1.01-1.30) (Table S3). Country-specific results indicated healthcare systems differences. In South Africa and Tanzania, crude HRs were 1.30 (95%CI: 1.09 – 1.55), and 1.13 (95%CI: 0.88 – 1.46), respectively. In Ethiopia, crude HR was 0.75 (95%CI: 0.57 – 0.99). Adjusting the regression analysis for sex and age did not change any of the effect estimates.

#### Table S3. Cox regression analysis of time period from positive TB test to initiation of treatment.

|  | Overall | | Ethiopia | | South Africa | | Tanzania | |
| --- | --- | --- | --- | --- | --- | --- | --- | --- |
|  | SOC  n = 502 | SeroSelectTB  n = 528 | SOC  n = 98 | SeroSelectTB  n = 108 | SOC  n = 281 | SeroSelectTB  n = 278 | SOC  n = 123 | SeroSelectTB  n = 142 |
| TB positive participants starting treatment, n (%) | 479 (93.6) | 500 (94.7) | 98 (100.0) | 108 (100.0) | 258 (91.8) | 259 (93.2) | 114 (92.7) | 133 (93.7) |
| Crude HR (95% CI) | 1 (Ref) | 1.14 (1.01, 1.30) | 1 (Ref) | 0.75 (0.57, 0.99) | 1 (Ref) | 1.30 (1.09, 1.55) | 1 (Ref) | 1.13 (0.88, 1.46) |
| Adjusted HR (95% CI) | 1 (Ref) | 1.15 (1.01, 1.30) | 1 (Ref) | 0.76 (0.57, 1.00) | 1 (Ref) | 1.30 (1.09, 1.54) | 1 (Ref) | 1.14 (0.89, 1.46) |

SOC = standard-of-care, TB = tuberculosis, HR = Hazard ratio, CI = confidence interval. Participants have been excluded if they have missing information on sex (n=0) or age (n=8); missing date of Xpert test and sputum smear test (n=1); date of treatment start is prior to date of testing (n=45); time to treatment was 365 days or more after date of testing (n=3).

Of note, information entered from Tanzania on date when Xpert test was performed (test date) was available for 2769 participants, whereas date when test result was received at the site (result date) was available for 3822 participants. Information on both dates was available for 2692 participants. Number of days from enrollment until testing and number of days until result was received, respectively, was calculated for participants with available test date and result date. However, the time interval was set to “missing” if the recorded date was before enrollment or more than 90 days after enrollment. Therefore, for each participant with missing number of days to testing and non-missing number of days to receipt of result (n=1082), number of days to testing was imputed by replacing the missing value with the observed value from a randomly drawn participant with information on both time intervals and with the same number of days until result was received as the participant having the missing value replaced.

To explore whether the missing values of time to testing depended on the reference laboratory testing the sample in addition to time to receipt of test result, we performed a sensitivity analysis where the imputed values were randomly drawn from participants with the same number of days until result was received and were tested at the same reference laboratory. In this analysis, number of days to testing with Xpert (median and IQR) in Tanzania did not change when the imputation of missing values was based on the reference laboratory testing the sample in addition to time to receipt of test result (median time to testing was 4 days (IQR 2-7 days) in both study arms).

### Feasibility

In resource limited, high TB-burden settings, the feasibility of implementing TB diagnostics is constrained by several factors including inadequate laboratory infrastructure, high costs of equipment and consumables, shortages of trained personnel, and weak sample transportation and referral systems that limit access to advanced diagnostic services ^6,7^.

To address these challenges and bring TB diagnostics closer to the community, strategies such as deploying portable molecular testing platforms, organizing mobile clinics equipped with digital X-rays or rapid tests, expanding the use of rapid diagnostic tools, and introducing point-of-care (POC) assays are recommended. These approaches help reduce diagnostic delays, lower patient costs, and ultimately enhance case detection and treatment initiation ^7,8^.

The 15-minute turnaround time, the simplicity of the procedure, and the non-cold chain requirement of the test make SeroSelectTB particularly well suited for primary level healthcare settings, where staff often have limited laboratory training and the sites have minimal infrastructure. These characteristics strongly support the ease of integrating the SeroSelectTB test into community-based and resource limited health systems.

A mock exercise designed as a refresher course using SeroSelectTB cassettes prepared with 16 different digitally designed test results was conducted to assess healthcare workers’ and nurses’ skills and confidence in reading test bands and interpreting results. Exercises were held during 2023 in Tanzania, Ethiopia, and South Africa in which all healthcare workers (n=57) involved in the trial from all sites participated. The exercise was successful with all participants demonstrating accurate reading, interpretation and documentation of results. In addition, the mock cassettes were used throughout the study in refresher courses and for training new healthcare workers at the sites. All healthcare workers were experienced with finger-prick blood collection, as this procedure is routinely conducted at primary healthcare facilities for malaria management. After the training sessions, the healthcare workers’ self-reported confidence in performing the test according to the instructions was high; the reading and interpretation of mock-test results were 100% correct.

### SeroSelectTB test accuracy

The SeroSelectTB assay accuracy was calculated using randomly selected subset of samples obtained from participants, all of whom were presumptive TB patients, recruited at our sites in Cape Town from September 2023 through December 2024. A total of 454 serum samples were subjected to double-blinded retesting at Lateral Flow Laboratories (Table S4). The sensitivity (calculated as True Positives/(True Positives + False Negatives) estimated among TB cases confirmed by Xpert or culture that had positive SeroSelectTB T1/T2 results was 53.2% (75/141). When considering confirmed TB cases who had positive T1/T2 and I-band results on SeroSelectTB test, the estimated sensitivity increased to 75.9% (107/141). The specificity in either case (calculated as True Negatives/(True Negatives + False Positives) was 49.5% (155/313). This means that an additional 22.7% (32 out of 141) of TB positive participants were detected due to the presence of reactive I-band on SeroSelectTB test, which would otherwise be missed. Of note, all 141 participants with HIV had positive I-band result on SeroSelectTB test.

#### Table S4. SeroSelectTB accuracy measure among all participants. SeroSelectTB test positive results are stratified by T1/T2 and I-band positivity.

| SeroSelectTB test results | | TB positive* | | TB negative* | |
| --- | --- | --- | --- | --- | --- |
| SeroSelectTB positive | SeroSelectTB T1/T2 positive | 75 | 107 | 48 | 158 |
|  | SeroSelectTB I-band only positive | 32 |  | 110 |  |
| SeroSelectTB negative | SeroSelectTB T1/T2 negative | 34 | | 155 | |
| Total | | 141 | | 313 | |

*Xpert OR culture results used as reference

When we restricted the accuracy estimates among HIV negative participants (n=313, Table S5), the estimated sensitivity was 68.8% (75/109), and specificity was 76.0% (155/204).

#### Table S5. SeroSelectTB accuracy measure among HIV negative participants.

| SeroSelectTB test results | TB positive* | TB negative* |
| --- | --- | --- |
| SeroSelectTB positive | 75 | 49** |
| SeroSelectTB negative | 34 | 155 |
| Total | 109 | 204 |

*Xpert OR culture results used as reference

** 1 participant was included in this group who had only reactive I-band on SeroSelectTB test

The sensitivity/specificity values obtained from the retesting investigations are lower than the accuracy data obtained in preliminary laboratory-based investigations using the prototype test platforms as presented above (see Assay development section). The SeroSelectTB prototype was developed using well-characterized sample sets from Africa and Asia, which were subjected to comprehensive analysis to ensure quality and consistency, and used as a "gold standard" for validating the assay. In contrast, the samples collected in our study were primarily relevant to the main objective of our study, to determine the extent to which SeroSelectTB can reduce health system diagnostic delay and expedite treatment. A sensitivity of 75.3% is above the current WHO TPP stipulated sensitivity of 65% ^9^ for a non-sputum-based rapid diagnostic assay. Previous iterations of the WHO TPP for TB Dx stated that the sensitivity 84% (range 66–87%) was required for a community-based triage or referral test for identifying people suspected of having TB as presented in the WHO TPP from 2014 ^3^. It was, however, argued in the 2014 TPPs that sensitivity targets might be too ambitious and that investigating the effect of screening tests with lower sensitivity levels may be useful. In addition, it was mentioned that a lower sensitivity may be acceptable if the test simultaneously detected other infections/diseases such as HIV or malaria.

I-band (Indicator-band) reactivity on the SeroSelectTB test indicates the need for additional TB testing. In the context of HIV coinfection, a person with TB may not be immunocompetent and thus not able to produce antibodies to the Mtb antigens in the SeroSelectTB test. In the current version of the assay, the I-band contains a single HIV-1 antigen that is indicative but not intended for diagnostics. I-band reactive samples included in the retesting subset were subjected to HIV testing using the current LFL HIV1-2 assay (rebranded at Atomo Diagnostics Ltd). The SeroSelectTB assay, when used for HIV screening alone in our study, yielded a sensitivity and specificity of 99.6% as per LFL HIV test conversion and WHO panel equivalence.

The value difference the SeroSelectTB test provides is reflected in the fact that only one out of nine presumptive TB patients (11.1%) included in the SOC arm at our study sites in South Africa who were referred for confirmatory testing based on clinical investigations alone (i.e., the routine screening method) had TB. In comparison, 75.9% of the true positives (i.e., patients confirmed by Xpert or culture to have TB) would have been referred for confirmatory testing had SeroSelectTB been used as a screening test. The lack of a rapid screening test at the primary healthcare level results in a significant use of expensive confirmatory Xpert tests with limited return in investment per positive TB case detected.

SeroSelectTB is suitable for use in primary care facilities as a POC test, and adds significant value in improving patient empowerment, compliance, and longitudinal management.

## References

1 Najdov S, Arsov J, Davcev J, Assefa T, Bikombo J, Kajeguka D, Okunola A, Nwamba W, Josifoski A, Holm-Hansen C. Enhancing data quality and remote accessibility in clinical trials: A SeroSelectTB case study in Ethiopia, Tanzania and South Africa. Applied Clinical Informatics 2025 – under review.

2 End TB strategy: WHO-HTM-TB-2015.19-eng.pdf, accessed September 2025. .

3 Target product profiles for tuberculosis screening tests. Geneva: World Health Organization; 2025. [https://iris.who.int/server/api/core/bitstreams/848c63db-fa29-459b-aaaf-1b758370711f/content].

4 Point-of-care tests for sexually transmitted infections: target product profiles. Geneva: World Health Organization; 2023.

5 Borain N, Petersen L, Plessis JD, Theron G, Holm-Hansen C. A rapid serological triage test for detecting active tuberculosis. BMJ Global Health. 2017;2(Suppl 2): A35.

6 World Health Organization. Global tuberculosis report 2024. Geneva: World Health Organization.

7 Ansu-Mensah M, Kuupiel D, Asiamah EA, Ginindza TG. Facilitators and barriers to in vitro diagnostics implementation in resource-limited settings: A scoping review. Afr J Prim Health Care Fam Med. 2023 Feb.

8 Byrne, R.L., Wingfield, T., Adams, E.R. et al. Finding the missed millions: innovations to bring tuberculosis diagnosis closer to key populations. BMC Global Public Health 2, 33 (2024).

9 World Health Organization. Target product profiles for tuberculosis diagnosis and detection of drug resistance. Geneva: World Health Organization. 2024.
