# Supplementary material for: Feasibility, accuracy, and effect of a rapid point-of-care serological test (SeroSelectTB) to identify presumptive pulmonary TB patients for confirmatory testing in Ethiopia, South Africa, and Tanzania: a multicenter, open-label, parallel-group, randomized, controlled trial
Source: eClinicalMedicine. 2026 Apr 25;95:103914. doi: 10.1016/j.eclinm.2026.103914 (PMC13129460; doi:10.1016/j.eclinm.2026.103914)
Supplement: Protocol_EDCTP_DM-#116831_SeroSelectTB [file mmc2.pdf]

EDCTP

European & Developing Countries Clinical Trials Partnership

**Template for essential information to be provided for proposals that include clinical trials and public health intervention studies, as well as other types of clinical studies and investigations, including cohort studies.**

## EDCTP grant number

**RIA2018D-2493 SeroSelectTB (application number 10196)**

## Full title of study and acronym

Evaluation of the feasibility, accuracy, and effect of a rapid point-of-care serological triage test for active TB (SeroSelectTB) in high burden, HIV-endemic African settings: a multi-centre, parallel-group, randomised, controlled trial

Acronym: SeroSelectTB

## Purpose and objective(s)

Worldwide over 15 million persons live with active TB. While the prevalence of latent TB is very high, only disease, and not latent infection, is treated in most high burdened countries (HBC).

A rapid triage test for detection of active TB, which can identify symptomatic individuals who require confirmatory diagnostic investigation, is a global priority for TB control. A rapid triage test can drastically reduce the burden on health systems and patients, and reduce diagnostic delay by expediting referral, confirmatory testing and timely commencement of adequate treatment. Triage tests, which may utilise point-of-care rapid lateral flow (LF) platforms, offer a promising alternative to conventional methods for TB diagnosis (i.e. smear microscopy and culture). However, the lack of accurate TB biomarkers has stalled the development of serological assays that meet the WHO-defined criteria for a triage test.

As an alternative, a rapid molecular diagnostic tool, Xpert and Xpert Ultra MTB/RIF, which accurately detects active TB, has been introduced in many countries as a replacement for smear microscopy and culture. However, Xpert/Ultra is not suited for use at health-posts without electricity or laboratory facilities, where the majority of patients present. In addition, the affordability of scaling-up Xpert is of concern in high TB-burden countries, where it could consume 20-80% of national TB budgets (1).

Therefore, to control the emergence and spread of antibiotic resistant TB in a cost-effective manner, there is an urgent need for a rapid, inexpensive triage test that can detect active TB at health-posts level and expedite referral to facilities supporting molecular diagnostics. An ideal cost-effective triage test should be as sensitive as Xpert and cost less than US $5 to reduce total diagnostic costs by 30-40%, potentially saving national TB programmes US $36 million/year (2).

The expected outcome of this project, the implementation of a triage test that will detect active TB at a low diagnostic cost, and expedite persons with TB-indicative symptoms to centralized healthcare facilities, will be beneficial irrespective of age and gender of patients. In addition, SeroSelectTB is sensitive among adults co-infected with TB/HIV.

**Primary objective** of field trial is to evaluate the diagnostic delay, measured as the time from TB diagnostic to TB treatment initiation between two groups of patients: those who were screened with SeroSelectTB triage test compared to patients who received the standard of care.

Persons with reactive SeroSelectTB test results will be referred for same-day confirmatory Xpert testing and treatment.

**Secondary objectives** are:

- To describe SeroSelectTB performance in field conditions, including sensitivity and specificity, positive and negative predictive values in the study populations.
- Collect blood and saliva samples from all consenting participants and create national biobanks, which will facilitate biological samples exchange and sharing with third parties for future research.
- Assess the added-value of using SeroSelectTB in healthcare systems in South Africa, Tanzania and Ethiopia through cost-effectiveness evaluation and inform international and national TB prevention programmes stakeholders.

**Study design**

This is an interventional, a multi-centre, two parallel-group, randomised, controlled field trial conducted in three countries: The South African Republic, United Republic of Tanzania and Republic of Ethiopia.

In the interventional arm participants will be screened using SeroSelectTB rapid triage test, and in the case of positive SeroSelectTB test results, will be referred to TB confirmation diagnostics at the referral centre where TB diagnosis will be confirmed by Xpert/Ultra molecular diagnostics (Figure 1).
In the standard of care arm patients will receive usual TB diagnostics: microbiology (culture-confirmed diagnostics) and/or microscopy (sputum smear-based diagnostics).

Figure 1. Flowchart of patients' enrolment in SeroSelectTB field trial.

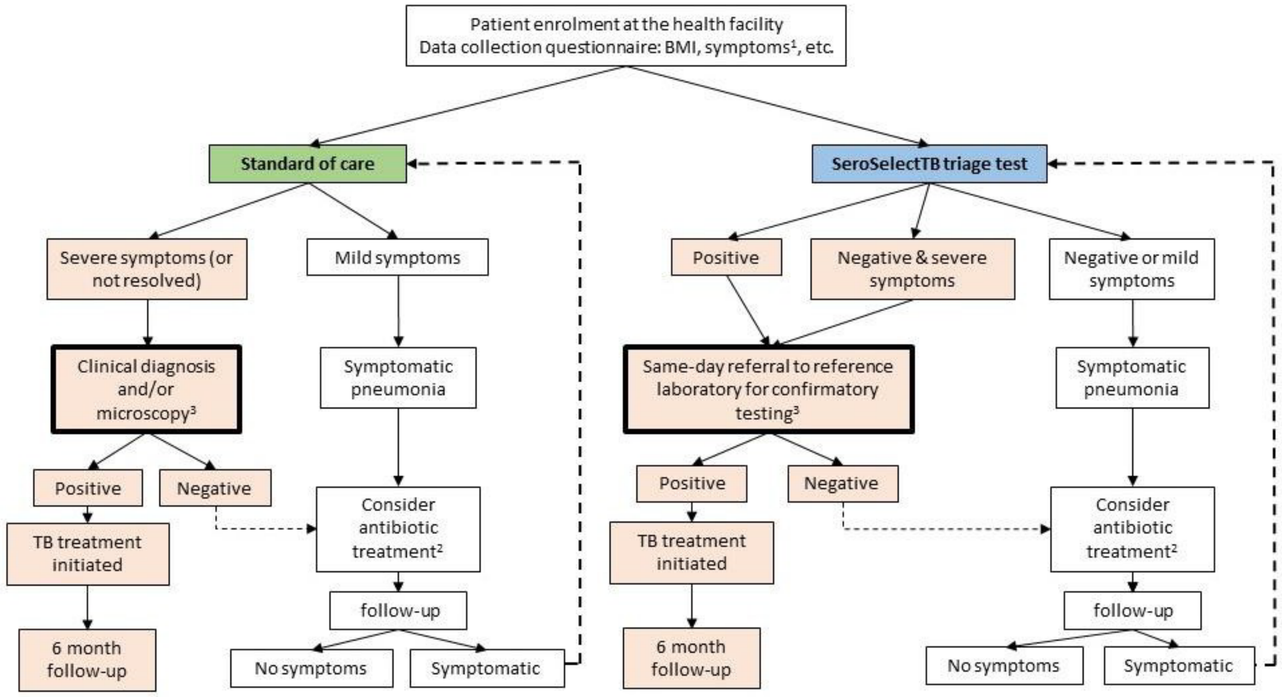


[1]Common symptoms: cough for two weeks, fever, shortness of breath, night sweats, weight loss.
[2]Antibiotic treatment for suspected pneumonia is standard of care.
[3]Biobank all samples (serum, saliva, sputum)

At inclusion, patients will be blinded to which diagnostic arm they are randomized: SeroSelectTB or standard of care arm. Healthcare workers performing the diagnostic test will not be blinded since it is unattainable to conceal if they are performing a microscopy/culture-based diagnostics or hand-held SeroSelectTB test.
Lab technicians at Lateral Flow Laboratories and RIVM will be blinded to patients' allocation when performing quality control analyses.

**Primary and secondary outcome measures**

Primary measure is time to diagnosis, measured in days, from initial presentation at a healthcare facility to confirmed TB diagnosis.

Primary measure will provide the answer to primary objective of this study, which will include all randomised patients enrolled in the study. Randomisation of patients will reduce the possibility of introducing bias in results, however, adjustment for gender and age as potential confounders is included in the statistical analysis plan.

Secondary measure is time to treatment, measured in days, from TB diagnosis to beginning of TB treatment restricted to subgroup of patients that have initiated TB treatment.

The secondary measure analysis will be adjusted for trial site, gender, age, severity of symptoms, and patients' education.

**Schedule for study conduct including timelines for key study milestones**

The total project duration and the duration of the proposed clinical study are provided in the Gannt chart. Here we list key field evaluation trial milestones defined relative to the staring date of the project (i.e. month 1, month 6 etc.):

- First Patient (or study subject), First Visit (FPFV): month 21
- Last Patient (or study subject), First Visit: month 55
- Last Patient (or study subject), Last Visit: month 62
- Planned Interim analyses (if any): no interim analyses were planned
- Final database lock: month 62
- Study analysis: initiated month 62
- Availability of final statistical study report: month 72
- Study closure (i.e. last patient off study and study documents are archive-ready): month 62
- Regulatory product dossier prepared: month 72

**Product(s) to be tested and supply (where applicable)**

| **Product name** | **Manufacturer** | **Details of product (approved for use/under development), GMP guarantee, supply and availability – see details above)** |
|---|---|---|
| SeroSelectTB | Lateral Flow Laboratory, Cape Town, South African Republic | Prototype completed, not registered.<br>GMP, ISO9001 and ISO13485 compliant. |
| Xpert | Cepheid, California, USA | Registered.<br>Supplied by national TB programmes at no cost to consortium. |

**Study population**

The study population will be symptomatic adults of both genders, 18 to 65 years old, who present at the selected healthcare facilities seeking medical attention due to persistent respiratory symptoms.
Inclusion criteria:

1) Signed written informed consent or witnessed oral consent in case of illiteracy, before undertaking any study-related activities.
2) Are unwell and are suspected to have TB or pneumonia.

Exclusion criteria:

1) Currently receiving TB treatment.
2) Have received equal or more than 30 days' doses of TB drugs treatment in the past three months, with the last dose received less than one month ago.

The subgroup analysis was planned for the time to treatment analysis of patients that have initiated TB treatment.

**Statistical analysis plan(ning) and power calculation (sample size)**

Assuming an effect size (hazard ratio) of 1.1, the sample size needed to obtain a power of 90% for the log-rank test is 2314 pulmonary tuberculosis (PTB) suspects in each arm of the study in each country. Using mean diagnostic delay duration of 22 days and clinically significant 5-day difference in duration of health systems' delay (3) between the two groups: SeroSelectTB and standard of care, the calculated sample size is 123 TB cases in each arm of the study in each country (at the significance level of 5%, and power 90%). Assuming that 1/26 (3), 1/23 (4) and 1/10 (5) of pulmonary tuberculosis (PTB) suspects have culture-positive TB, 3321, 2829 and 1230 PTB suspects in each study arm must be screened in Ethiopia, Tanzania and South Africa, respectively, in order to identify 123 TB cases. However, in order to obtain the necessary power for the log-rank test, in South-Africa 2314 PTB suspects will be included. We will aim to enroll between 10% and 20% more participants than needed by power calculation to ensure losses to follow-up won't have impact on statistical analysis.

In 2016, 7301, 28,920 and 9600 TB suspects were reported from selected study sites in Ethiopia, Tanzania and South Africa, respectively (3, 4, 5). Among suspected TB cases 268, 1257 and 960 were bacteriologically confirmed TB cases in Ethiopia, Tanzania and South Africa, respectively. This calculation provides reasonable confidence that enough TB confirmed cases will be recruited over the 2 years filed evaluation period.

Statistical analysis: The participants will contribute person-time from inclusion in the study until they initiate TB treatment (primary outcome) or for a maximum of six months. The time to treatment initiation in the two groups will be represented graphically with Kaplan-Meier curves, and compared with a log-rank test. Furthermore, the two groups will be compared using Cox regression. We will use a stratified Cox model with site (South Africa, Tanzania, Ethiopia) as the strata. Additional adjustment will be made for gender and age.

In order to further explore the distribution of time to diagnosis, we will perform secondary analyses limited to the participants that have initiated TB treatment. We will use quantile regression to evaluate whether the median time to treatment differs between the two groups. Since those with the longest time to treatment initiation are of particular concern, we will also assess the 75th percentile. These analyses will be adjusted for site, gender, age, severity of symptoms, and education.

**Recruitment strategy and retention**

An information leaflet considering 'Myths and Facts' on TB diagnostic testing has been produced, will be submitted to ethics committees for approval, and will be used for public dissemination as a community engagement tool. In addition, each participant will receive an information booklet that is produced and will be submitted to ethics committees for approval, about the SeroSelectTB clinical trial. The Myths and Facts leaflet and the SeroSelectTB trial booklet are attached to this application (EDCTP grant application number 10196) under "Budget justification and support document".

Healthcare facilities and clinical centres involved in this study have access to more than 7,000 suspected TB cases per year, which ensures a large enough population to identify patients with active pulmonary TB disease.

Loss to follow-up will be minimized by involving the community upfront through our planned community engagement plan, performing follow-up interviews with patients on TB treatment (DOT patients will be follow-up by data collection regarding their treatment), and/or conducting home visits.

**Recruitment site selection**

Healthcare facilities and centres were selected based on reported high TB burden and previous experience with clinical and field TB trials. Annually, more than the double of confirmed TB cases are reported in the selected sites, which makes recruitment of needed number of cases (N=123) over two years' time feasible.

## Patient and/or community involvement

Community engagement plan consists of preparation and ethical approval of 'Myths and Facts' leaflet and study booklet explaining the purpose of SeroSelectTB test evaluation trial that will be performed in the community.

Community involvement programmes of trial sites will be engaged in study preparation, conduct and dissemination of results to study participants after the study end. For example, each participant will receive a 'Welcome letter' at enrolment with the study booklet, and at the end of follow up will receive a 'Thank you letter'.

## Clinical Study Sponsor

Norwegian Institute of Public Health (NIPH), Oslo, Norway.

Recent trials with sponsorship:

Effectiveness Trial to Evaluate Protection of Pregnant Women by Hepatitis E Vaccine in Bangladesh; ClinicalTrials.gov Identifier: NCT02759991

Comparing 3 Antibiotic Regimes for Erythema Migrans in General Practice; ClinicalTrials.gov Identifier: NCT01368341

Fractional Dose Tetravalent A, C, Y, W135 Meningococcal Polysaccharide Vaccine; ClinicalTrials.gov Identifier: NCT00271479

NIPH was sponsor, or co-sponsor, in over 30 clinical trials registered at the ClinicalTrials.gov portal: https://clinicaltrials.gov/ct2/results?cond=&term=Norwegian+Institute+of+Public+Health&cntry=NO&state=&city=&dist

## Ethical and regulatory approval

Ethics review will be conducted by the local and national ethics committees. We anticipate protocol, healthcare utilization questionnaire (more details in the Excellence section (6, 7)), and community engagement materials submission prior to the commencement of the project, with a total planned time for ethical committees review and approval of six months.

Usual ethics timelines are:
Norwegian Institute of Public Health/Regional Committees for Medical Research Ethics - South East, Norway: 2-3 months

Stellenbosch University/Faculty of Health Sciences (HREC), and City of Cape Town Department of Health, South Africa: 1-3 months

Kilimanjaro Christian Medical University College/ Kilimanjaro Christian Medical University College Research Ethics and Review Committee (KCMUCo CRERC), National Medical Institute for Research (NIMR), and Tanzania Food and Drugs Authority (TFDA), Tanzania: 1-3 months

Armauer Hansen Research Institute/AHRI/ALERT Ethics Review Committee, Amhara Region Public Health Institute, and the National Ethics Review Committee, Ethiopia: up to 6 months

**Clinical Study Registration**

The study is registered in ClinicalTrials.gov (https://clinicaltrials.gov/) in accord with standard procedure at the Norwegian Institute of Public Health. ClinicalTrials.gov Identifier: NCT04752592

**Study safety**

This trial does not contain any experimental intervention that will influence patients care. Collection of biological samples will be in accordance with WHO guidelines on blood sampling for research purposes.

**Study management**

Study management is described in WPs 1 and 4, and in communication between the study sponsor (NIPH) and trial centers at SU, KCMUCo and AHRI.

Study protocol, communication plan (including a RACI chart below), and the generation of a database, will be completed in a joint consensus process including the trial sponsor and trial centers.

The RACI chart that stipulates roles and responsibilities of each partner in the consortium regarding the field trial will form the backbone of consortium's communication plan to ensure trial integrity.

| *Work package* | *Activity* | *NIPH* | *SU* | *KCMUC* | *AHRI* | *KNCV* | *Lateral Flow Labs* | *InVivo BioTech* | *Aether* |
|---|---|---|---|---|---|---|---|---|---|
| *WP1 Project management* | *Project organization and management* | *R* | *A* | *A* | *A* | *I* | *I* | *I* | *I* |
| | *Trial oversight and Risk management* | *R* | *R* | *R* | *R* | *C* | *R* | *R* | *R* |
| *WP2 Capacity building* | *Training for healthcare workers and lab technologists* | *C* | *R* | *R* | *R* | *I* | *I* | *I* | *I* |
| | *Community engagement (participant's letters, trial brochure, etc.)* | *C* | *R* | *R* | *R* | *I* | *I* | *I* | *I* |
| *WP3 SeroSelectTB production* | *SeroSelectTB test production* | *I* | *I* | *I* | *I* | *I* | *R/A* | *R/A* | *I* |
| | *SeroSelectTB test production quality control* | *C* | *I* | *I* | *I* | *R* | *R/A* | *R/A* | *I* |

| | | | | | | | | | |
|---|---|---|---|---|---|---|---|---|---|
| | *Laboratory-based quality control* | *I* | *I* | *I* | *I* | *R* | *I* | *I* | *I* |
| *WP4 SeroSelectTB evaluation* | *Patient enrollment and randomisation* | *C* | *R* | *R* | *R* | *I* | *I* | *I* | *R* |
| | *Field evaluation trials:* | | | | | | | | |
| | *1) Field trial* | *A* | *R* | *R* | *R* | *C* | *I* | *I* | *R* |
| | *Data collection and analysis:* | | | | | | | | |
| | *1) Patients' individual data capture* | *A* | *R* | *R* | *R* | *I* | *I* | *I* | *R* |
| | *2) Biobank samples collection and maintenance* | *A* | *R* | *R* | *R* | *C* | *I* | *I* | *I* |
| | *Results dissemination (publications, conferences, etc)* | *R/A* | *R/A* | *R/A* | *R/A* | *R* | *R* | *R* | *R* |
| *WP5 Stakeholder engagement and Commercial-isation* | *Stakeholder engagement: WHO, FIND, IUATLD, national level* | *R/A* | *R* | *R* | *R* | *C* | *C* | *C* | *C* |
| | *Communicatio n with regulatory authorities (e.g. SAHPRA, NB)* | *R/A* | *I* | *I* | *I* | *I* | *C* | *C* | *C* |

R - Responsible - partner(s) who carries out a process or assigned tasks.
A - Accountable - partner(s) who is ultimately accountable for a process or a task being completed appropriately.
C - Consulted - partner(s) not directly involved in a process or a task, but who is consulted (e.g. a subject-matter expert).
I - Informed - partner(s) who will be informed about the output of a process or a task.

All decisions and changes to communication plan or management decisions in the study will be discussed and agreed during a face to face meetings or via teleconference calls.

## Study monitoring and quality control

A written contractual agreement ('Consortium Agreement') will be signed by the consortium partners, and specific Clinical Trial Agreements will be signed by NIPH and clinical trial centers: Stellenbosch University, South African Republic, Kilimanjaro Christian Medical University College, United republic of Tanzania, and Armauer Hansen Research Institute, Republic of Ethiopia, before the study start. A primary investigator (PI) at the trial center will be responsible for reaching milestones, informing WPs and consortium Steering Committee (SC) leaders on progress, and keeping accounts as defined in project guidelines.

On-site and in-house monitoring of trial data and protocol compliance will be performed according to study analysis protocol, written study plans and standard operating procedures (SOPs). An independent Data Monitoring Committee (DMC) composed of study site PIs, individuals with relevant expertise (e.g. data management and/or clinical background), and a statistician from NIPH, will be formed as part of WP4, and will report directly to the SC. The role of DMC is to monitor data collection, data integrity and project timelines related to data collection. In addition, Field Evaluation Trial Committee (FETC), formed by local healthcare workers and clinicians, will be responsible for trial sites patients' safety and integrity. The FETC will closely work with DMC, and report to the SC.

A risk-based approach to monitoring will be used. A secured cloud system will be used to register screened participants, randomization and assign to a trial arm. It is anticipated that most of data management, including cloud system design and maintenance, data cleaning, creation of listings for in-house monitoring, developing randomisation schedule, progress reports, Statistical Analysis Plan development, tables, figures, and presentations, and evaluation of primary and secondary end points, will be overseen by DMC and FETC (WP4).

The study sponsor will host the database and maintain the oversight over recruitment, protocol compliance, study conduct, data correctness and integrity. Monitoring (on-site and remotely) and data management will be in the remit of DMC and FETC (WP4).

Quality control will be performed at Lateral Flow Laboratory as part of their SOPs which are ISO9001:2015 and ISO13485 compliant. Independent quality monitoring will be performed by KNCV (WP3).

## References


1. Pantoja A, Fitzpatrick C, Vassall A, Weyer K, Floyd K. Xpert MTB/RIF for diagnosis of tuberculosis and drug-resistant tuberculosis: a cost and affordability analysis. Eur Respir J. 2013;42(3):708-20.
2. Pantoja A, Kik SV, Denkinger CM. Costs of novel tuberculosis diagnostics--will countries be able to afford it? J Infect Dis. 2015;211 Suppl 2: S67-77.
3. Yimer S, Holm-Hansen C, Yimaldu T, Bjune G. Evaluating an active case-finding strategy to identify smear-positive tuberculosis in rural Ethiopia. Int J Tuberc Lung Dis. 2009;13(11):1399-404.
4. Nyombi B. Personal communication. 2017.
5. Theron G. Personal communication. 2017.
6. Mauch V, Woods N, Kirubi B, Kipruto H, Sitienei J, Klinkenberg E. Assessing access barriers to tuberculosis care with the tool to Estimate Patients' Costs: pilot results from two districts in Kenya. BMC Public Health. 2011; 11:43.
7. TBCTA U. The Tool to Estimate Patients' Costs 2008 [Available from: https://static-content.springer.com/esm/art%3A10.1186%2F1471-2458-11-

**Annex 1: Mandatory deliverables for clinical studies**

For each clinical study, the following mandatory deliverables (with the indicated title and scope as defined) have to be included in the proposal:

1. 'First study subject approvals package'
(prior to enrolment of first study subject):

a. Final version of study protocol as approved by first regulator / ethics committee(s).
b. There is no need to change deliverables unless there are major amendments that change the study design, patient population, risk- benefit profile, sample size, study medications/ devices, original treatment plan or allocation; or study interruption due to unforeseen circumstances.

b. Registration number of clinical study in a WHO- or ICMJE- approved registry that also allows later posting of study results.
c. Approvals required for invitation / enrolment of first subject in at least one clinical centre (if applicable): ethics committees, national competent authorities and copies of opinion or confirmation by the competent Institutional Data Protection Officer and/or authorization or notification by the National Data Protection Authority. If the position of a Data Protection Officer is established, its opinion/confirmation that all data collection and processing will be carried out according to EU and national legislation

*Applicants should also include an* **All approvals package** *as a deliverable where this is applicable (eg. multi-country studies).*

2. 'Midterm recruitment report'
Deliverable to be scheduled for the time point when 50% of the study population is expected to have been recruited. The report shall include an overview of recruited subjects by study site, potential recruiting problems and, if applicable, a detailed description of implemented and planned measures to compensate delays in the study subject recruitment.

3. 'Report on status of posting results'
Report on the status of posting results in the study registry/ies (including timelines when final posting of results is scheduled after end of funding period). To be scheduled for the time of expected results posting or for the last months of the project, whichever comes earlier.
