# Supplementary material for: Feasibility, accuracy, and effect of a rapid point-of-care serological test (SeroSelectTB) to identify presumptive pulmonary TB patients for confirmatory testing in Ethiopia, South Africa, and Tanzania: a multicenter, open-label, parallel-group, randomized, controlled trial
Source: eClinicalMedicine. 2026 Apr 25;95:103914. doi: 10.1016/j.eclinm.2026.103914 (PMC13129460; doi:10.1016/j.eclinm.2026.103914)
Supplement: Equitable_Partnership_Declaration_Form_CHH [file mmc3.pdf]

Equitable Partnership Declaration

If any questions do not apply to your study, please indicate "N/A" for "not applicable.
For more information on how to complete this form see the Information for Authors document.

Researcher considerations

1. Please detail the involvement that researchers who are based in the country or countries of study had during a) study design; b) clinical study processes, such as processing blood samples, prescribing medication, or patient recruitment; c) data interpretation; and d) manuscript preparation, commenting on all aspects. If they were not involved in any of these aspects, please explain why.

   This should include a thorough description of their leadership roles in the study. Are local researchers named in the author list or the acknowledgements, or are they not mentioned at all (and, if not, why)? Please also describe the involvement of early career researchers based in the location of the study. Some of this information might be repeated from the Contributors section in the manuscript. Note: we adhere to ICMJE authorship criteria for naming authors on a paper.

Author initials for Post 1

Norway:
Carol Church Holm-Hansen: CHH
Ida Laake: IL
Solomon Abebe Yimer: SAY

Belgium:
Miloje Savic: MS

South Africa:
Grant Theron: GT
Anna Olutoyin Okunola: AOO
Welile Vumile Nwamba: WVN
Andrew Medina-Marino: AMM
Nick Borain: NB
Julianne Du Plessis: JDP

Ethiopia:
Kidist Bobosha: KB
Tamirat Assefa: TA
Melese Yeshambaw: MY
Mekdelawit Wondiyfraw: MW
Yonas Abebe Habtesilase: YAH
Samuel Asmamaw Yesuf: SAYE

Tanzania:
Balthazar Melchior Nyombi: BMN
Debora Charles Kajeguka: DCK
Hadija Hamisi Semvua: HHS
Jemrath Bikombo: JB

Flavia Mayo: FM
Gaudensia Alois Olomi: GAO
Arnold Japhet Ndaro: AJN

Austria:
Aleksander Josifoski: AJ

Macedonia:
Sasho Najdov: SN
Jovan Davchev: JD
Jordanco Arsov; JA

Netherlands:
Kristin Kremer: KK

Germany:
Tim Welsink: TW
Stephan Grunwald: SG

a) Study design:

CHH, MS, and SAY conceptualized the study. The study was conceptualized before the consortium was established. The principal investigators for every partner institution contributed to the study proposal that was funded by EDCTP.

CHH, MS, SAY, IL, KB, BMN, and GT were responsible for methodology and study design. In addition, the coauthors responsible for conducting the field investigations (KB, TA, MY, MW, YAH, SAYE, SAY, BMN, DCK, HHS, JB, FM, GAO, AJN, GT, AOO, WVN, and AMM) contributed to modifying/optimizing the methodology and study design in response to challenges met at the healthcare facilities and logistics.

KB, BMN, GT, NB, JDP, TW, SG, and CHH were responsible for financial resources, local staff resources and laboratory equipment

CHH was responsible for external funding acquisition, overall project administration, and reporting to EDCTP.

b) Clinical study processes:

KB, TA, MY, MW, YAH, SAYE, SAY, BMN, DCK, HHS, JB, FM, GAO, AJN, GT, AOO, WVN, and AMM conducted the field investigations.

KB, GT, BMN, MS, and CHH supervised the study.

NB, JDP, TW, and SG were responsible for the SeroSelectTB assay manufacturing.

AJ, SN, JD, and JA were responsible for software and development of the randomization app and automatic QC reporting system.

AJ was responsible for project website (www.seroselecttb.org) development and maintenance.

c) Data interpretation:

AJ, MS, SN, JD, and JA were responsible for data curation.

KB, TA, BMN, JB, FM, GT, AOO, and WVN were responsible for data validation and interpretation.

IL, MS, AJ, SD, JD, and JA conducted the formal data analysis and visualization.

d) Manuscript preparation:

MS, AJ, and CHH wrote the original draft manuscript.

All authors reviewed, edited, and approved the final version of the manuscript.

2. How was funding used to remunerate and enhance the skills of researchers in the countries of study? And how was funding used to improve research infrastructure at the study sites?

   Potentially effective investments into long-term skills and opportunities within local institutions could include training or mentorship in analytical techniques and manuscript writing, opportunities to lead all or specific aspects of the study, financial remuneration rather than requiring volunteers, and other professional development and educational opportunities.

   Improvements to research infrastructure could include funding extended trial designs (eg, platform trials), establishment of long-term contracts for research staff, building research facilities, and setting up local control of funding allocation.

**Skills:** Extra workload allowances for all healthcare workers, and a small portion of team members' salaries, and travel costs to scientific conferences was provided. We held training courses and refresher courses at partner institutions and at field sites. EDCTP did not provide funds for post graduate education; several PhD candidates were financed through their institutions and used day from the SeroSelectTB project in the research. Responibility for specific tasks was delegated to team members throughout the project under the guidance of the principal investigators at each partner institution and the project lead. Laboratory technologists from the Armauer Hansen Research Institute (AHRI) in Ethiopia (a consortium partner) received training in rapid lateral flow technology and manufacturing processes at Lateral Flow Laboratories in Cape Town with regard to future technology transfer to the Vaccine, Diagnostic & Medical Device Research and Development Directorate (VDMdRDD) at AHRI. This technology transfer will increase accessibility.
**Research infrastructure:**<br>EDCTP did not provide funding for building research facilities in this project. Funds allocated to the partners were controlled in accord with local institutional guidelines, and reported annually to the Norwegian Institute of Public Health (project "owner") and EDCTP (project sponsor). All annual technical and financial reports were approved by the finance department at NIPH and EDCTP.<br>The SeroSelectTB project emphasized community participation. Community engagement materials addressing tuberculosis myths/facts and the clinical trial were designed, provided in English, Afrikaans, isiXosha, Amharic and Kiswahili, and distributed to the respective catchment areas (see attachment to Supplemental file). These materials were used throughout the project and continue to be used by community healthcare workers. These materials have increased awareness about primary healthcare facilities, and visits to the facilities for symptoms indicating tuberculosis as well as other infections have increased significantly.

3. How did you safeguard the researchers who implemented the study?

   Please describe how you guaranteed safe working conditions for study staff, including provision of appropriate personal protective equipment, protection from violence, and prevention of overworking.

Working conditions were determined by the national and regional health authorities responsible for our sites. Protective latex gloves to protect healthcare workers during sample

collection and testing were provided. Protection from violence and prevention of overworking were in accord with local policies instituted at all sites included in the project. All partners secured insurance that covered researchers and participants.

Benefits to the communities and regions of study

4. How does the study address the research and policy priorities of its location?

How were the local priorities determined and then used to inform the research question? Who decided which priorities to take forward? Which elements of the study address those priorities?

Local (i.e. national) priorities were in accord with the national TB control program guidelines in the partner countries where the study was performed. Additional advice was provided and reviews were conducted regularly by local community advisory boards.

5. How will research products be shared in the community of study?

For instance, will you be providing written or oral layperson summaries for non-academic information sharing? Will study data be made available to institutions in the region(s) of study? The Lancet Global Health *encourages authors to translate the summary (abstract) into relevant languages after paper editing; do you intend to translate your summary?*

The progress and results of the SeroSelectTB project have been shared via newsletters, the website (www.seroselecttb.org), and participation at consortium meetings (with participation from local staff, health authorities, etc.) throughout the study. The project website will be active after the conclusion of the project via our partner Lateral Flow Laboratories. We will translate the summary of our multicenter publication and post the summaries on our website. All community engagement materials were prepared in English, Kiswahili, Amharic, Afrikaans and isiXhosa.
All project activities and results have been shared locally, and information presented on the project website is suitable for laypersons/non-academic audiences.

6. How were individuals, communities, and environments protected from harm?

a) How did you ensure that sensitive patient data were handled safely and respectfully? Was there any potential for stigma or discrimination against participants arising from any of the procedures or outcomes of the study?

We adhered to EU DPIA (data protection impact assessment) guidelines in accordance with regulations at the Norwegian institute of Public Health (SeroSelectTB project “owner”). All data shared between partners was anonymised and scrambled. There was no potential for stigma or discrimination arising from the SeroSelectTB study.

b) *Might any of the tests be experienced as invasive or culturally insensitive?*

No

c) *How did you determine that work was sensitive to traditions, restrictions, and considerations of all cultural and religious groups in the study population?*

We worked closely with community advisory boards and community healthcare workers with who have extensive experience addressing cultural and religious traditions in our populations.

d) *Were biowaste and radioactive waste disposed of in accordance with local laws?*

Yes. All biowaste (used test cassettes and disposable blood collection equipment were discarded in accordance with national and institutional regulations.

e) *Were any structures built that would have impacted members of the community or the environment (such as handwashing facilities in a public space)? If so, how did you ensure that you had appropriate community buy-in?*

No

f) *How might the study have impacted existing health-care resources (such as staff workloads, use of equipment that is typically employed elsewhere, or reallocation of public funds)?*

No public funds were used in the SeroSeelctTB project. Healthcare workers received allowances from project funds. The SeroSelectTB test requires no laboratory equipment. All other procedures (microscopy and confirmatory testing) were performed in accord with the National TB Control Program policies in partner countries. The SeroSeelctTB project did not interfere with or influence these procedures/policies.

7. Confirm that local ethics review was sought, and please provide the approval number. If not sought, please explain why.

International, national and institutional ethical approvals were obtained from the following boards:

Norwegian Institute of Public Health (NIPH), Oslo Norway:

- REK South East: Regional Committee for Medical Research Ethics South East Norway (national ethical approval), ref # 60638 SeroSelectTB: granted 19 May 2020 through 31 December 2023, and 6 November 2023, extension approval 6 November 2023, through 31 December 2025.
- DPIA (Data Protection Impact Assessment), approval granted 15 December 2020 for duration of project, registered in the NIPH archival system (P360, ref # 17/10773; project data base # 2818).

Stellenbosch University, Cape Town, South Africa:

- HREC (institutional ethical approval), project #15189/ethics ref # M20/06/017, granted 19 October 2020, renewed annually.

- Cape Town City Health (city ethical approval, ref #27975, granted 15 December 2020, renewed annually.

Kilimanjaro Christian Medical University College, Mossi, Tanzania:

- CRERC (institutional ethical approval), certificate # 2480, granted 16 September 2021, renewed annually.
- NIMR (national ethical approval), certificate # NIMR/HQ/R.8a/Vol.IX/3713, granted 1 July 2021, renewed annually.
- COSTECH (Tanzania Commission for Science and Technology approval), ref # 2022-114-NA-2021-293, granted 11 February 2022, renewed annually.
- TMDA (Tanzania Medicines and Medical Devices Authority, clinical trial authorization), certificate # TZ22CT0008, granted 9 June 2022, renewed biannually.

Armauer Hansen Research Institute, Addia Ababa, Ethiopia:

- MoSHE/MoE (National ethical approval, Ministry of Science and Technology and Ministry of Education), ref # 04/246/61/21, granted 29 April 2021, renewed annually.
- AAERC-AHRI/ALERT (institutional ethical approval), ref # PO29/20, granted 3 September 2020, renewed annually.

Secondary analyses

8. Have the data analysed in your study been extracted from another source, such as a national survey, rather than being directly collected by the authors of this paper?

No

If the authors of this paper were not involved in data collection, how were the findings interpreted with sufficient contextual knowledge?
The Lancet Global Health *believe contextual understanding is crucial for informed data analysis and interpretation*.

Data collection was conducted solely by the authors of this paper (see post 1).

---

9. Please provide the title (eg, Dr/Prof, Mr/Mrs/Ms/Mx), name, and email address of an author who can be contacted about this statement.

Name: Professor/Dr/Senior Scientist, Carol Church Holm-Hansen
Email: car-h-h@online.no (carol.holm-hansen@fhi.no)

10. Finally, please provide the title and name of an author from one country of study who has seen and approved this form.

Name: Dr Miloje Savic. Email: miloje.savic@yahoo.com
