# Supplementary material for: Feasibility, accuracy, and effect of a rapid point-of-care serological test (SeroSelectTB) to identify presumptive pulmonary TB patients for confirmatory testing in Ethiopia, South Africa, and Tanzania: a multicenter, open-label, parallel-group, randomized, controlled trial
Source: eClinicalMedicine. 2026 Apr 25;95:103914. doi: 10.1016/j.eclinm.2026.103914 (PMC13129460; doi:10.1016/j.eclinm.2026.103914)

TUBERCULOSIS

DIAGNOSTIC TESTS AND CLINICAL TRIALS

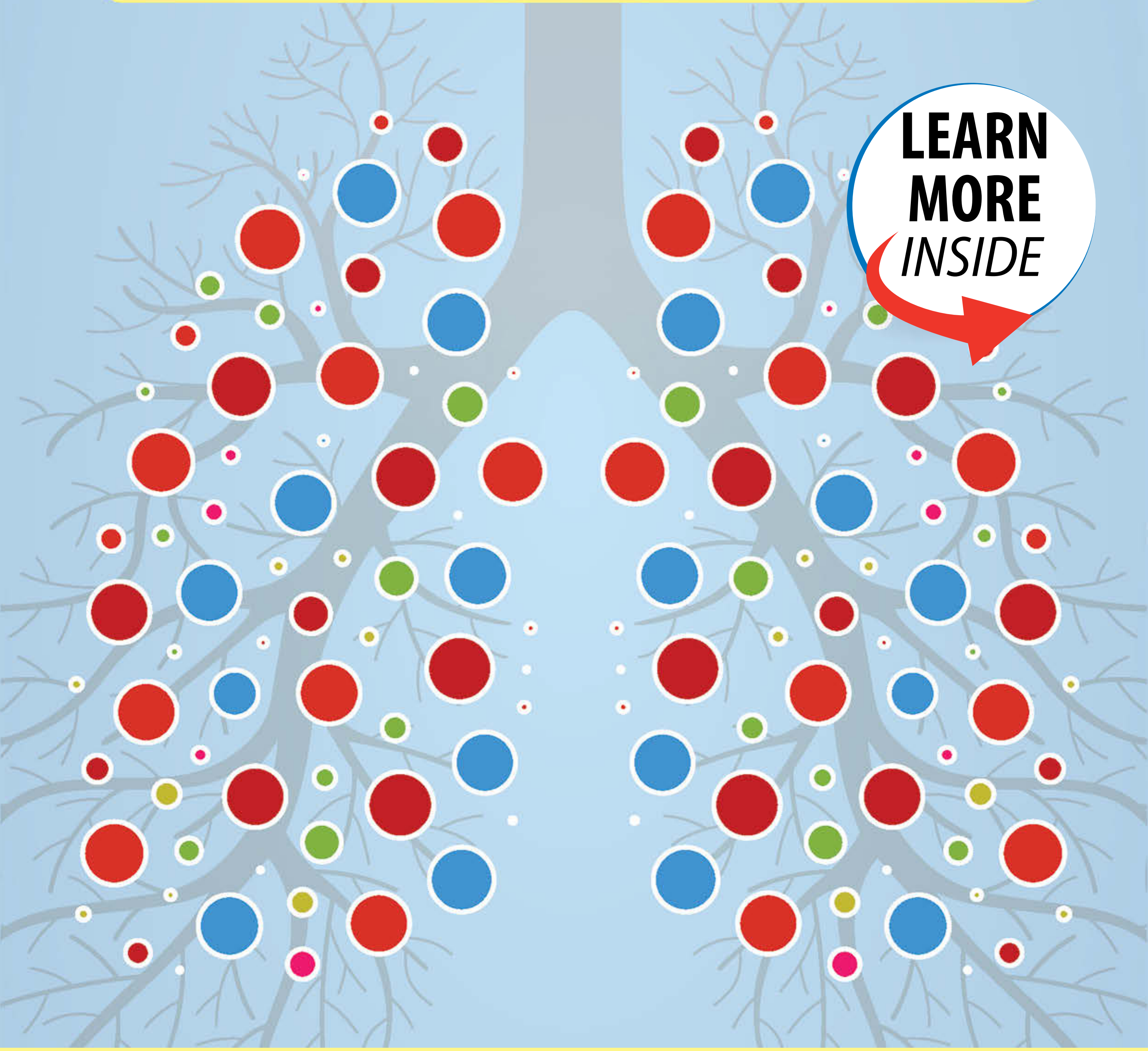

LEARN MORE *INSIDE*

PREVENTING THE SPREAD OF TB THROUGH NEW DIAGNOSTICS

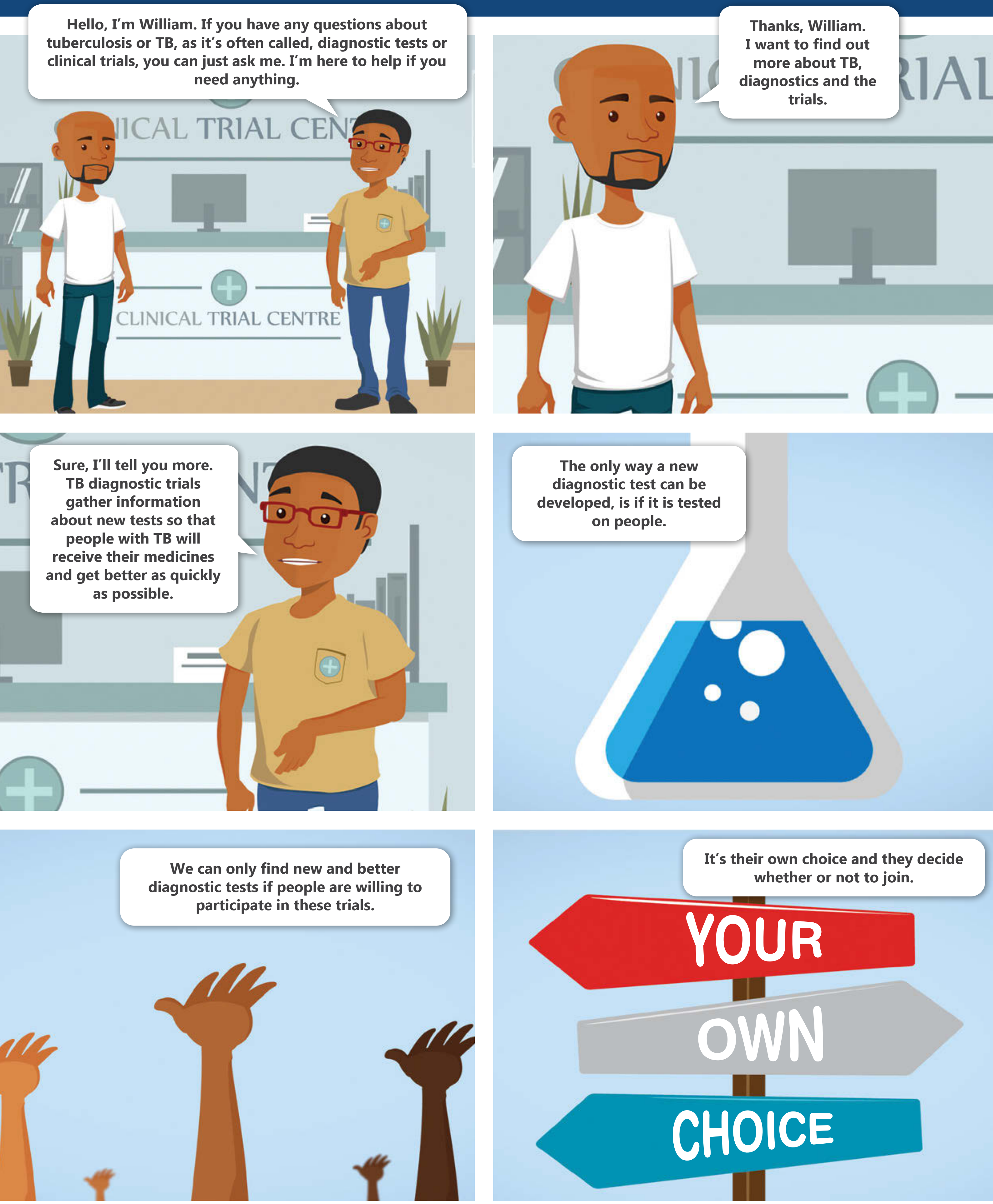
Hello, I'm William. If you have any questions about tuberculosis or TB, as it's often called, diagnostic tests or clinical trials, you can just ask me. I'm here to help if you need anything.
CLINICAL TRIAL CENTRE
Thanks, William. I want to find out more about TB, diagnostics and the trials.
Sure, I'll tell you more. TB diagnostic trials gather information about new tests so that people with TB will receive their medicines and get better as quickly as possible.
The only way a new diagnostic test can be developed, is if it is tested on people.
We can only find new and better diagnostic tests if people are willing to participate in these trials.
It's their own choice and they decide whether or not to join.
YOUR
OWN
CHOICE

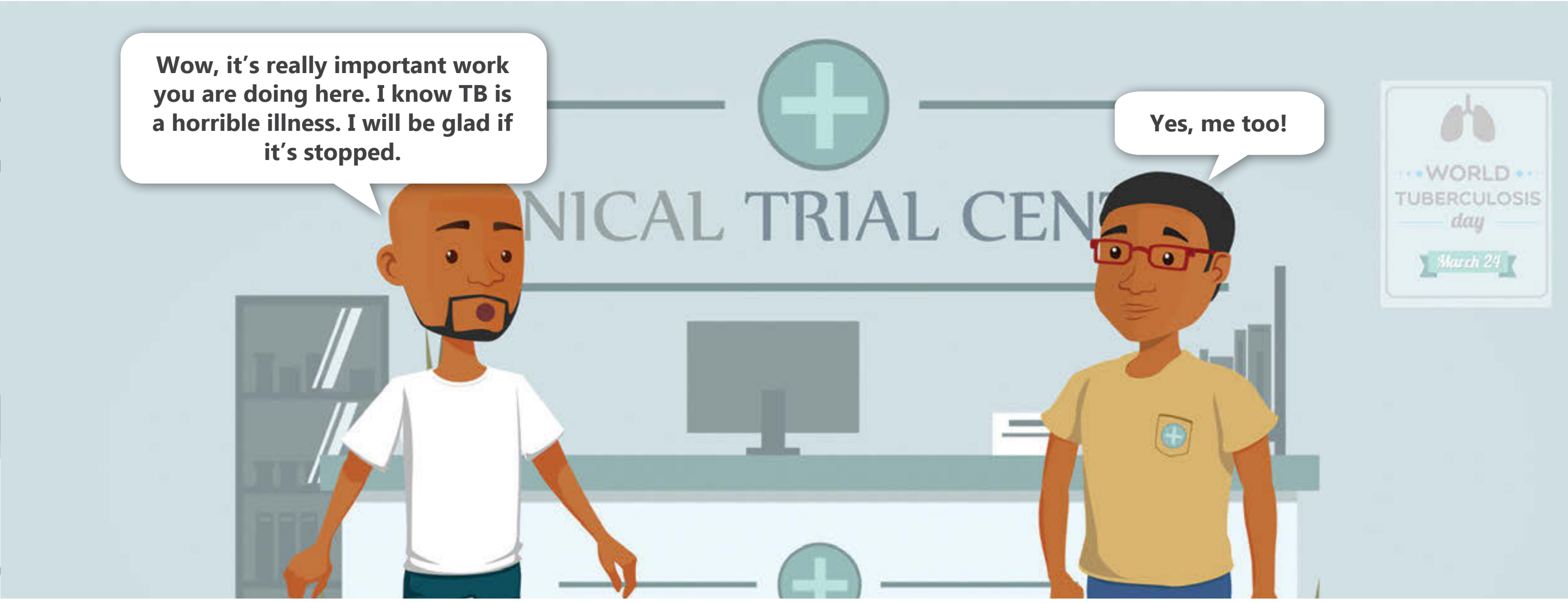
Wow, it's really important work you are doing here. I know TB is a horrible illness. I will be glad if it's stopped.
Yes, me too!
NICAL TRIAL CEN
WORLD TUBERCULOSIS day
March 24

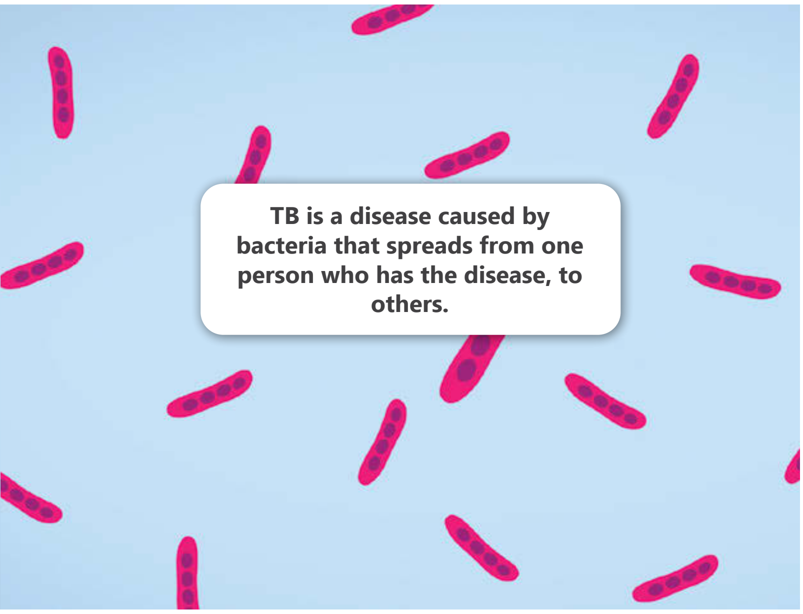
TB is a disease caused by bacteria that spreads from one person who has the disease, to others.

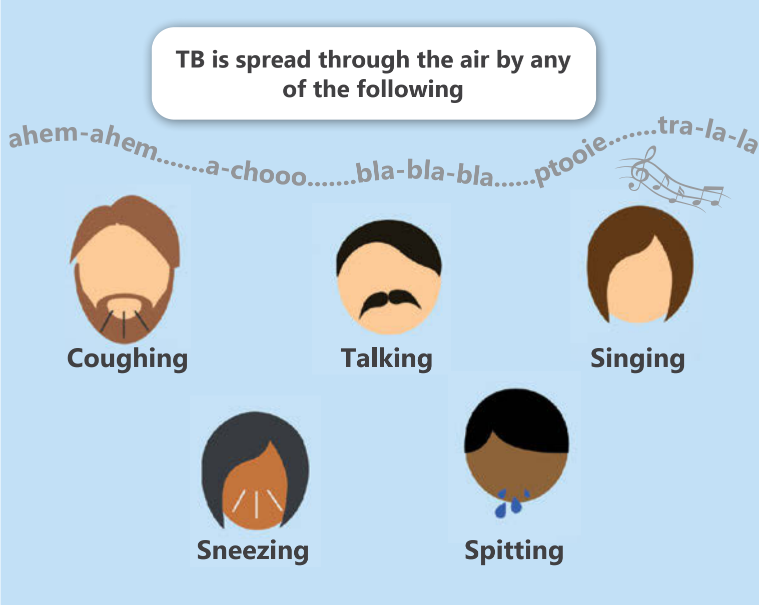
TB is spread through the air by any of the following
ahem-ahem.......a-chooo.......bla-bla-bla......ptooie.......tra-la-la
Coughing
Talking
Singing
Sneezing
Spitting

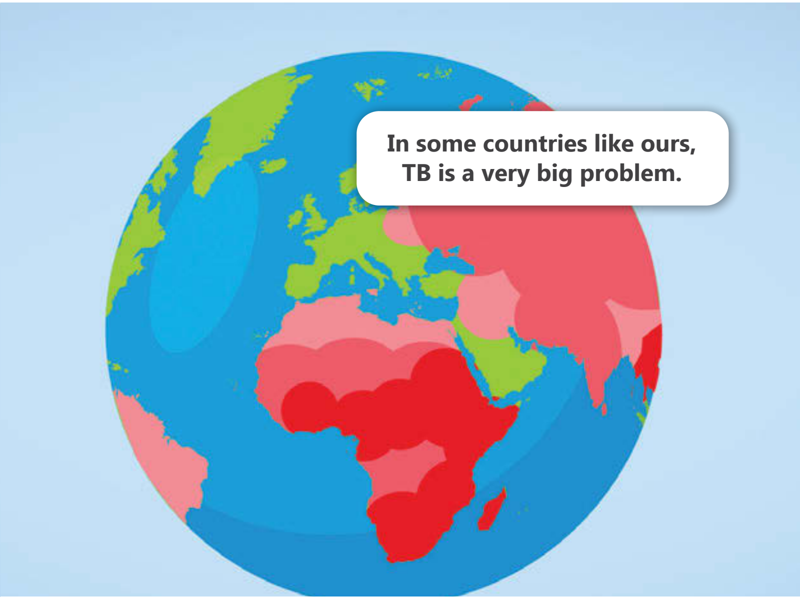
In some countries like ours, TB is a very big problem.

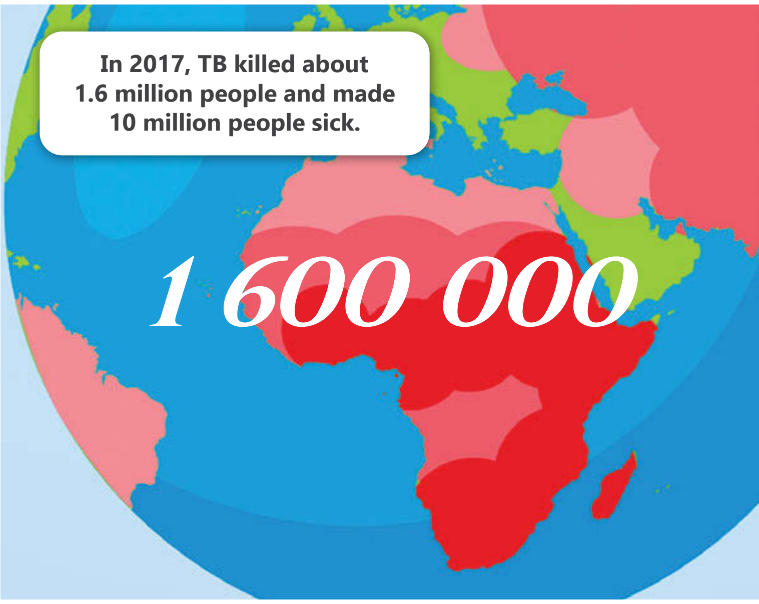
In 2017, TB killed about 1.6 million people and made 10 million people sick.
1 600 000

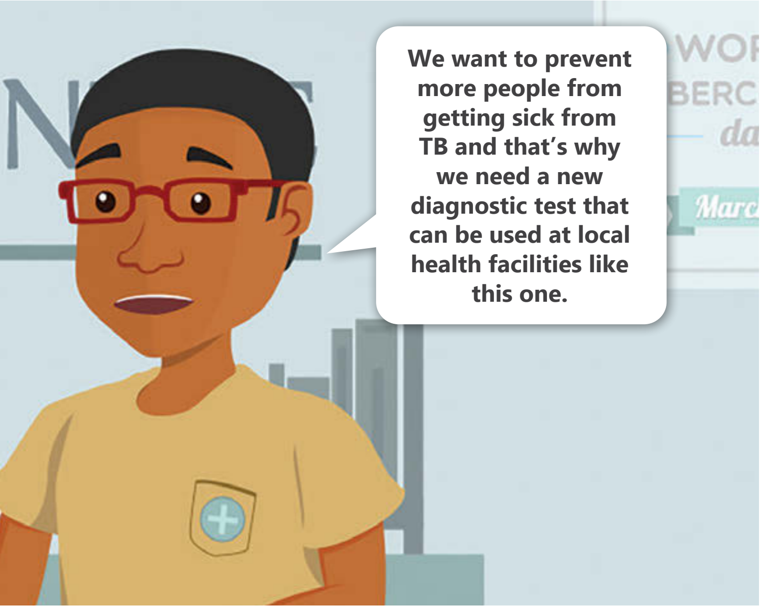
We want to prevent more people from getting sick from TB and that's why we need a new diagnostic test that can be used at local health facilities like this one.

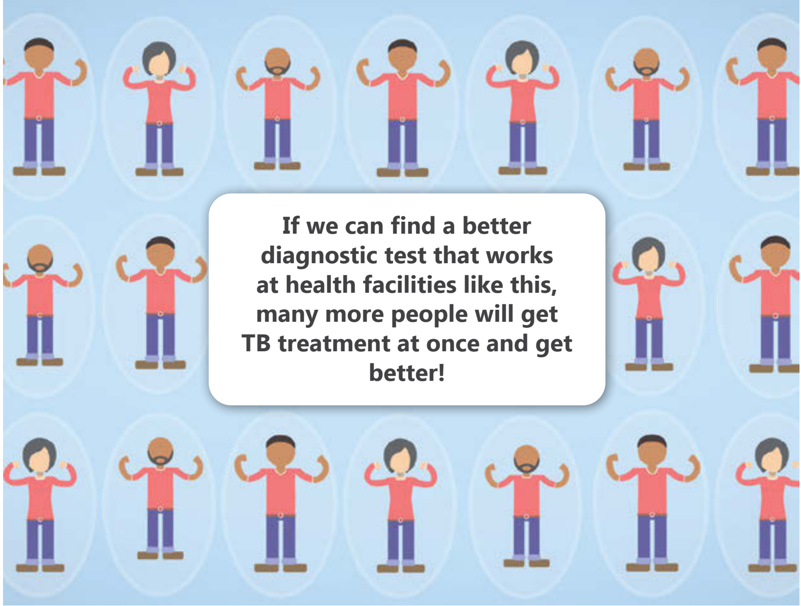
If we can find a better diagnostic test that works at health facilities like this, many more people will get TB treatment at once and get better!

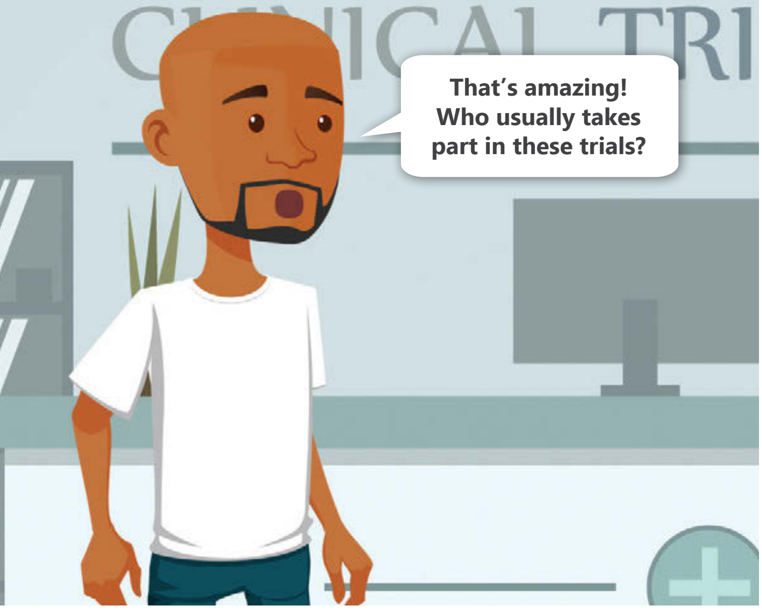
That's amazing! Who usually takes part in these trials?

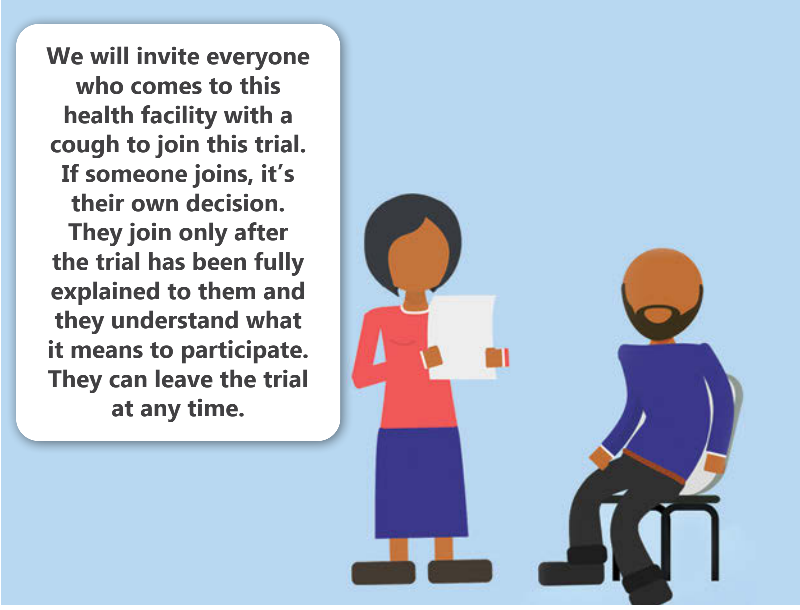
We will invite everyone who comes to this health facility with a cough to join this trial. If someone joins, it's their own decision. They join only after the trial has been fully explained to them and they understand what it means to participate. They can leave the trial at any time.

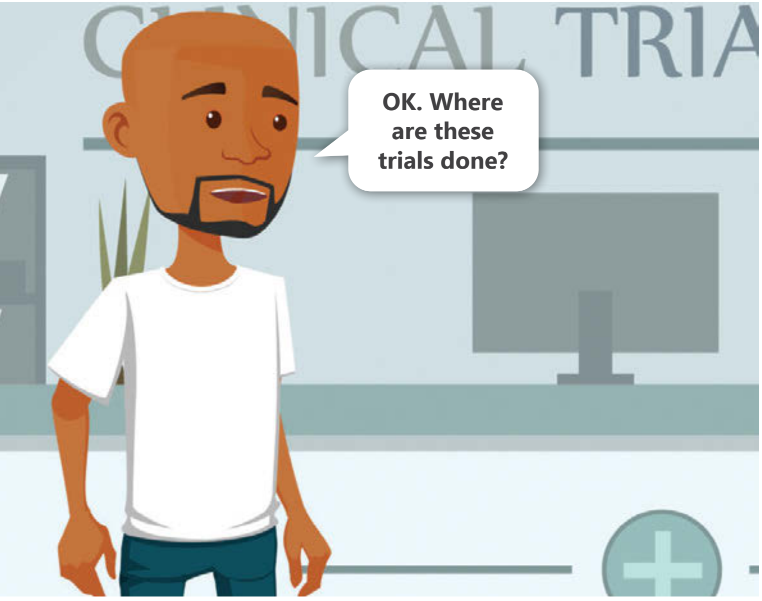
OK. Where are these trials done?

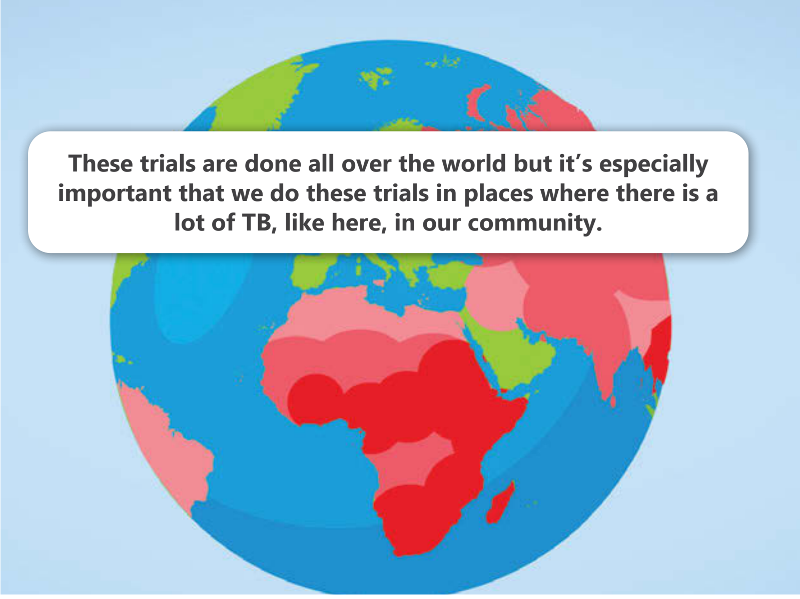
These trials are done all over the world but it's especially important that we do these trials in places where there is a lot of TB, like here, in our community.

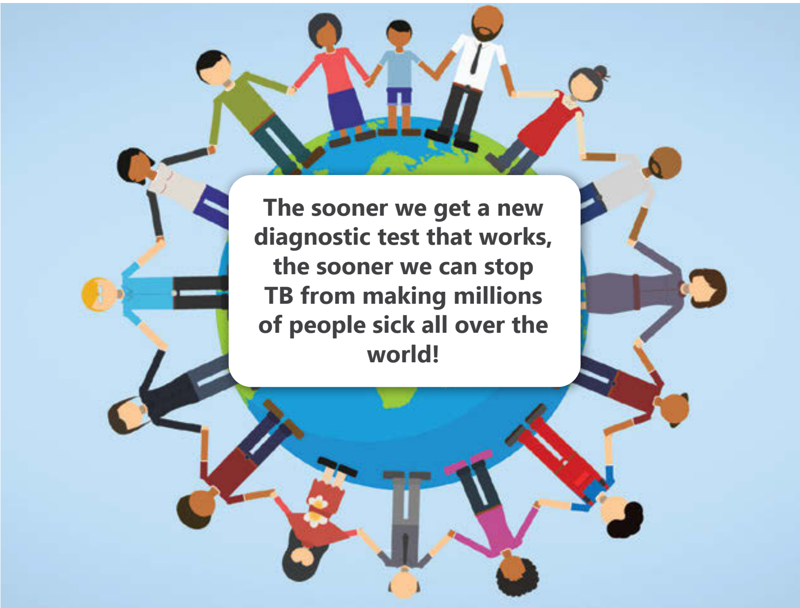
The sooner we get a new diagnostic test that works, the sooner we can stop TB from making millions of people sick all over the world!

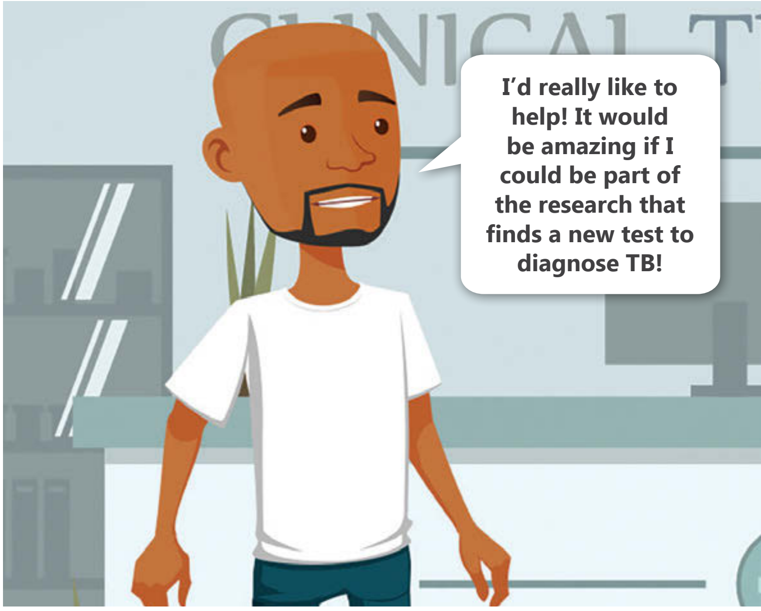
I'd really like to help! It would be amazing if I could be part of the research that finds a new test to diagnose TB!

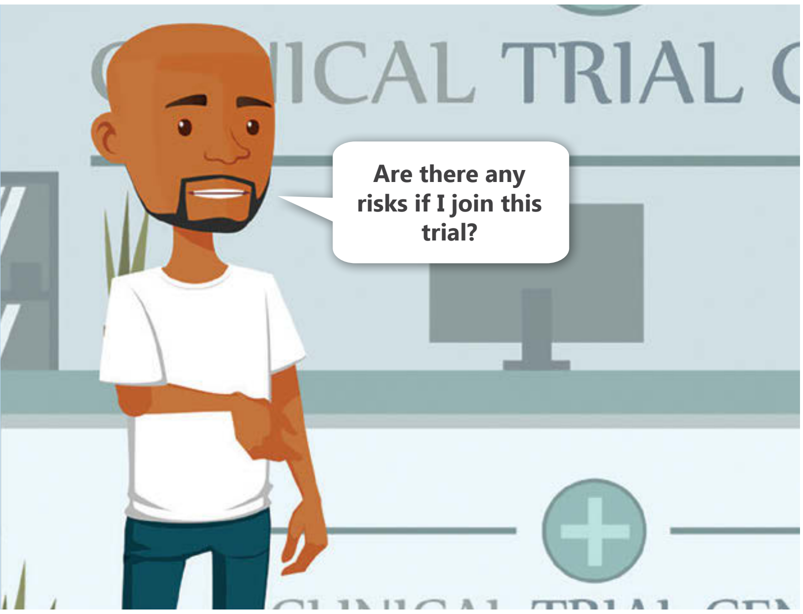
Are there any risks if I join this trial?

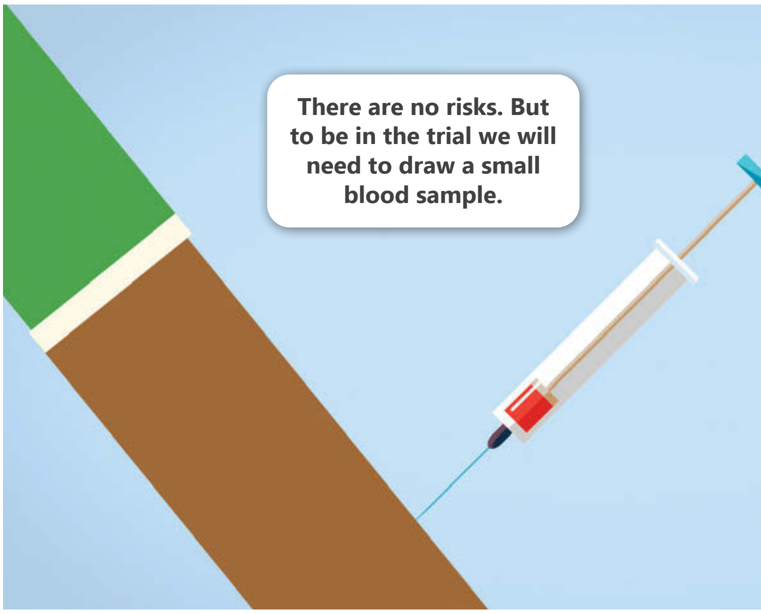
There are no risks. But to be in the trial we will need to draw a small blood sample.

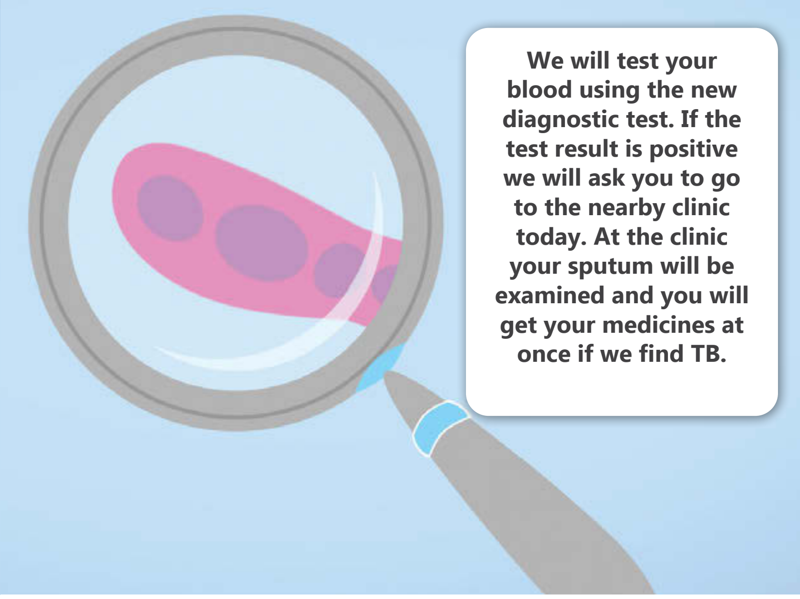
We will test your blood using the new diagnostic test. If the test result is positive we will ask you to go to the nearby clinic today. At the clinic your sputum will be examined and you will get your medicines at once if we find TB.

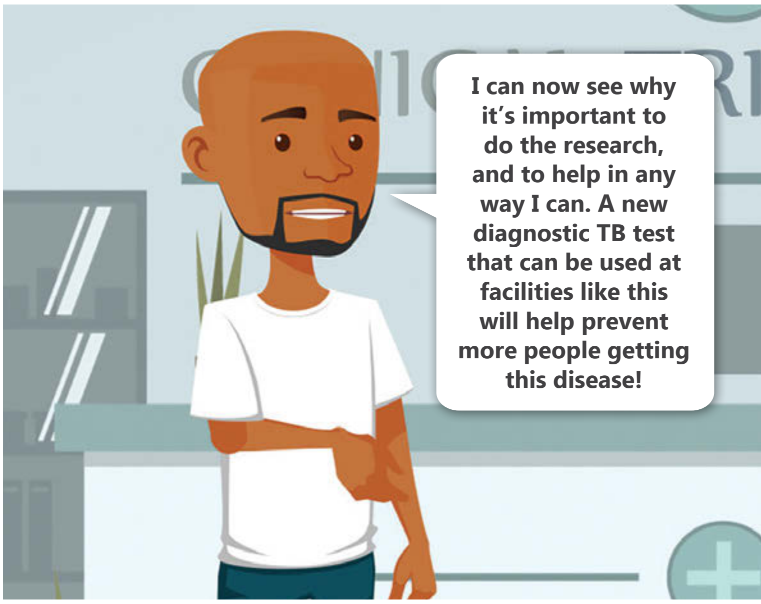
I can now see why it's important to do the research, and to help in any way I can. A new diagnostic TB test that can be used at facilities like this will help prevent more people getting this disease!

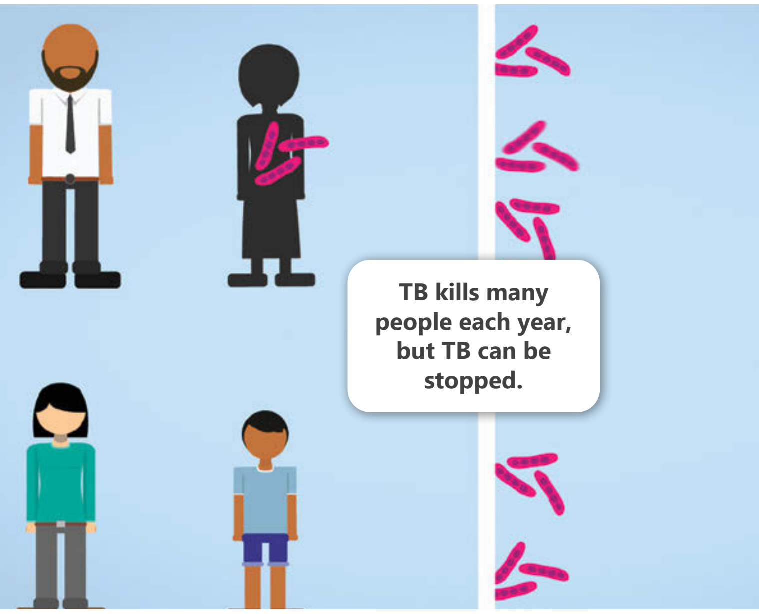

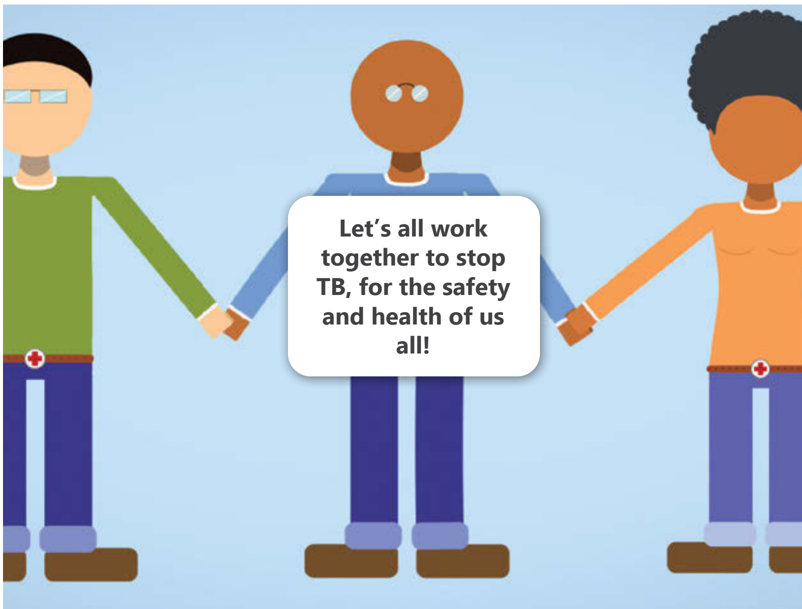

ADAPTED FROM COMMUNITY ENGAGEMENT MATERIALS KINDLY PROVIDED BY IAVI

notes

THE SEROSELECTTB CONSORTIUM MISSION IS TO DEVELOP A RAPID AND AFFORDABLE TB DIAGNOSTIC TEST

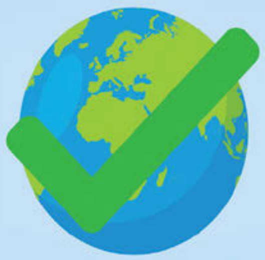

Supplement: CE-Booklet_SeroSelectTB_English (electronic) [file mmc4.pdf]
