# Supplementary material for: Feasibility, accuracy, and effect of a rapid point-of-care serological test (SeroSelectTB) to identify presumptive pulmonary TB patients for confirmatory testing in Ethiopia, South Africa, and Tanzania: a multicenter, open-label, parallel-group, randomized, controlled trial
Source: eClinicalMedicine. 2026 Apr 25;95:103914. doi: 10.1016/j.eclinm.2026.103914 (PMC13129460; doi:10.1016/j.eclinm.2026.103914)
Supplement: CE-MythsFacts_SeroSelectTB_English (electronic) [file mmc5.pdf]

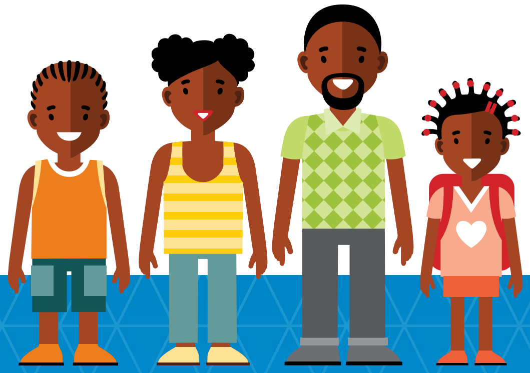

MYTHS AND FACTS

about clinical trials of TB Diagnostic Tests

MYTH

People in developing countries are used as “guinea pigs” in diagnostic test clinical trials.

FACT

The very first clinical trials are usually held in the country where the diagnostic test was first discovered.

After a diagnostic test is first tested for accuracy using samples in the laboratory, it needs to be tested in countries where the disease is common to see if it detects the disease among people with symptoms. **For TB diagnostic test trials, this needs to be in a community with high rates of TB.**

MYTH

Blood collected in clinical trials is later sold to make profit.

FACT

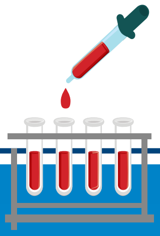

Blood is never sold or used in any illegal way!

Blood is needed in this clinical trial in order to perform the diagnostic test. This is a new test that detects TB antibodies in blood rather than TB bacteria in sputum. The new test is rapid, can be performed at health facilities like this one, and does not require any laboratory equipment.
The test uses the least amount of blood possible. **Some blood will be stored so that it can be tested again later for further development and/or improvement of the test, but only with the participant’s permission.**

MYTH

TB diagnostic tests used during clinical trials give people TB.

FACT

The TB diagnostic test cannot give you TB!

During the clinical trial, only a small amount of blood will be taken from the participants. The blood will be taken from a vein in the participant’s arm using a sterile single-use vacutainer and syringe. The participant may feel a very slight “prick” from the very thin sterile needle, but **will not get TB or any other infection.**

MYTH

If and when the diagnostic test is proven to work, it will not benefit the community and participants in the clinical trials.

FACT

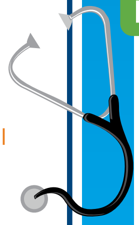

Once we prove that the new diagnostic test is accurate and suitable for use at health facilities like this one, the test will be made available to any community whose government approves and licenses it. Our mission is to develop a new diagnostic test that is available and accessible to all the communities who need it the most, so that **persons with TB can receive their medicines and get healthy as soon as possible.**

MYTH

Only people who are sick with TB can participate in TB diagnostic test clinical trials.

FACT

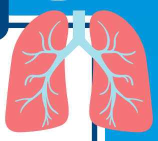

A TB diagnostic test clinical trial finds out if a new test can accurately detect TB. So, the people participating in a TB diagnostic test clinical trial will all have symptoms of lung diseases that may or may not be due to TB. **The new test will detect only TB**, and only these persons will get TB medicines. Persons without TB may get other medicines.

Adapted from Community Engagement materials kindly provided by IAVI

iavi
