# Supplementary material for: Feasibility, accuracy, and effect of a rapid point-of-care serological test (SeroSelectTB) to identify presumptive pulmonary TB patients for confirmatory testing in Ethiopia, South Africa, and Tanzania: a multicenter, open-label, parallel-group, randomized, controlled trial
Source: eClinicalMedicine. 2026 Apr 25;95:103914. doi: 10.1016/j.eclinm.2026.103914 (PMC13129460; doi:10.1016/j.eclinm.2026.103914)
Supplement: mSeroSelectTB SAP v05_280225 [file mmc6.pdf]

Title

Statistical Analysis Plan for the clinical trial, “Evaluation of the feasibility, accuracy, and effect of a rapid point-of-care serological triage test for active TB (SeroSelectTB) in high burden, HIV-endemic African settings: a multi-centre, parallel-group, randomised, controlled trial”

Version history

V05: 28 February 2025

V04.1: 25 February 2025

V04: 25 February 2025

V03.1: 19 February 2025

V03: 14 February 2025

V02: 12 February 2025

V01: December 2024 – January 2025

Lead author: Miloje Savic

Contributing authors: Jovan Davcev, Jordanco Arsov, Carol Holm-Hansen, Ida Laake, Tamirat Assefa, Kidist Bobosha, Balthazar Nyombi, Aleksandar Josifoski

Contents

Title 1

Version history 1

1 Introduction 3

2 Objectives and endpoints 4

2.1 Study design 4

3 Statistical hypotheses 5

4 Analysis set 5

4.1 Study sites and population 5

4.2 Analysis Population 6

5 Statistical Analyses 6

5.1 Methodology 6

5.2 Handling missing data 7

6 Primary objective and endpoints 7

- Main analytical approach 9

7 Secondary objective 10

8 Tertiary objective 10

9 Demographic and Socioeconomic variables 11

10 Sample size calculations 12

11 Supporting documentation 12

12 References 13

1 Introduction

Worldwide over 15 million persons live with active tuberculosis (TB). While the prevalence of latent TB is very high, only disease, and not latent infection, is treated in most high TB-burden countries.

A rapid triage test for the detection of active TB, which can identify symptomatic individuals who require confirmatory diagnostic investigation, is a global priority for TB control. A rapid triage test can drastically reduce the burden on health systems and patients, and reduce diagnostic delay by expediting referral, confirmatory testing and start of treatment. Triage tests, which may utilise point- of-care rapid lateral flow (LF) platforms, offer a promising alternative to conventional methods for TB diagnosis (i.e. smear microscopy and culture). However, the lack of accurate TB biomarkers has stalled the development of serological assays that meet the WHO-defined criteria for a triage test.

As an alternative, a rapid molecular diagnostic tool, Xpert and Xpert Ultra MTB/RIF (Xpert/Ultra), which accurately detects active TB, has been introduced in many countries as a replacement for smear microscopy and culture. However, Xpert/Ultra is not suited for use at health posts without electricity or laboratory facilities, where the majority of patients present. In addition, the affordability of scaling-up Xpert is of concern in high TB-burden countries, where it could consume 20-80% of national TB budgets (1).

Therefore, to control the spread of infection and the emergence of antibiotic-resistant TB in a cost-effective manner, there is an urgent need for a rapid, inexpensive triage test that can detect active TB at the health posts level and expedite referral to facilities supporting molecular diagnostics. An ideal cost-effective triage test should be as sensitive as Xpert/Ultra and cost less than US $5 to reduce total diagnostic costs by 30-40%, potentially saving national TB programmes US $36 million/year (2).

We developed the SeroSelectTB rapid LF serological triage test as an add-on to, and *not* a replacement for, downstream tests including AFB smear microscopy, Xpert/Ultra, and/or culture. The SeroSelectTB test has been subjected to clinical field evaluations in which diagnostic delay will be compared between intervention and standard-of care study arms

The expected outcome of this project, the implementation of a triage test that will detect active TB at a low diagnostic cost and expedite persons with symptoms indicative of TB to centralized healthcare facilities for confirmatory testing, will be beneficial irrespective of age and sex. In addition, SeroSelectTB detects TB among adults co-infected HIV.

2 Objectives and endpoints

Objectives	Endpoints
Primary objective:<br>To evaluate health systems' diagnostic delay	1) Time to TB treatment initiation
	2) Time to routine TB test performed
	3) Time to confirmatory Xpert/Ultra test performed
Secondary objective:<br>To evaluate TB patient diagnostic delay	Self-reported time period during which the patients experienced symptoms before seeking medical care at the health care facility
Tertiary objective:<br>To evaluate SeroSelectTB test diagnostic accuracy	Estimate of sensitivity, specificity, positive and negative predictive values

Primary objective:

To evaluate health systems' diagnostic delay, measured as the time from reporting to the primary healthcare facility to TB treatment initiation among presumptive TB patients in both arms of the trial.

In addition, two additional end points were defined as time to testing by 1) routine AFB microscopy or 2) confirmatory Xpert/Ultra testing in order to capture different diagnostic delay time periods depending on the health care protocol used in routine practice.

Secondary objective:

To evaluate TB patient diagnostic delay by measuring time that elapsed from the onset of symptoms until the patient self-reported to the closest health care facility.

Tertiary objective:

To evaluate SeroSelectTB test diagnostic accuracy measures: sensitivity, specificity, positive and negative predictive values as compared to AFB smear microscopy and/or Xpert/Ultra.

2.1 Study design

This is an interventional, multi-centre, two parallel-group, randomised, controlled field trial conducted at selected sites in the Republic of South African Republic, the United Republic of Tanzania, and the Federal Democratic Republic of Ethiopia.

In the intervention arm, participants will be screened using the SeroSelectTB rapid triage test. Persons with positive SeroSelectTB test results will be referred for confirmatory diagnosis by AFB smear microscopy at the next level of healthcare, and thereafter by Xpert/Ultra at centralized referral facilities (Figure 1).

In the standard-of-care arm, presumptive TB patients will be referred for confirmatory testing as described above based on clinical symptoms.

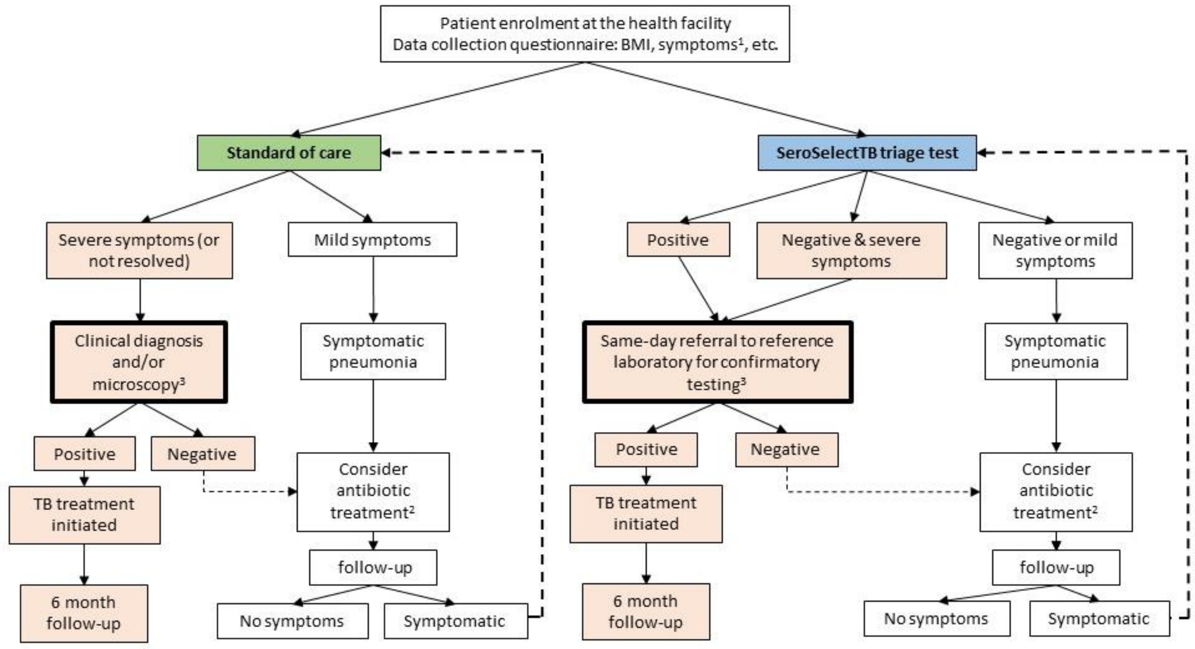

[1]Common symptoms: cough for two weeks, fever, shortness of breath, night sweats, weight loss.
[2]Antibiotic treatment for suspected pneumonia is standard of care.
[3]Biobank all samples (serum, saliva, sputum)

Figure 1. Flowchart of patient enrolment in SeroSelectTB field trial.

Presumptive TB patients will be randomized into the intervention or standard-of-care arm after providing written informed consent and completing the questionnaire.

3 Statistical hypotheses

Unless specified, all statistical tests will be conducted as two-sided with a significance level of alpha equal to 0.05.

4 Analysis set

4.1 Study sites and population

The study population will include adults 18 years of age and older who self-reported to the selected healthcare facilities seeking medical attention due to persistent respiratory symptoms, fulfil all inclusion criteria, and do not meet any of the exclusion criteria (as defined below).

Inclusion criteria:

1) 18 years of age or above
2) Signed written informed consent or witnessed oral consent in case of illiteracy, before undertaking any study-related activities
3) Unwell and suspected to have TB or pneumonia

Exclusion criteria:

1) Currently receiving TB treatment
2) In the past 3 months, have been on TB treatment for 30 or more days, with the last dose taken less than one month ago

4.2 Analysis Population

Analysis set will include anonymized, individual-level data from research sites in the Republic of South Africa, the United Republic of Tanzania, and the Federal Democratic Republic of Ethiopia covering entire intended-to-treat enrolled population.

5 Statistical Analyses

5.1 Methodology

Cox proportional hazard regression using the shared frailty model

The Cox proportional hazard (PH) regression was originally developed to predict time to death for actuary calculations [10].

The Cox PH regression describes the relationship between the event incidence, as expressed by the hazard function, and a set of covariates (different risk factors) [11, 12].

The shared frailty model is an extension of the Cox PH model that adds a frailty multiplier to the hazard function for every subject that reflects the subject's 'frailty' or risk level. Related subjects are assigned the same frailty coefficient. A frailty term of <1 means less risk, and a frailty term of >1 means higher risk [17]. Hence, the frailty term deals with an unobserved heterogeneity concern that is not addressed by the covariates, and is caused by relatedness among observations such as multiple patient visits, applications sold by the same vendors, patients treated by the same care provider, etc.

5.2 Handling missing data

No missing data will be imputed, unless key analytical variables are missing for 20% or more of study participants in a relevant data set. A linear regression imputation technique may be used in instances when 20% or more of data is missing in an analytical data set (8).

6 Primary objective and endpoints

To evaluate health systems' diagnostic delay, measured as the time from reporting to the primary healthcare facility to TB treatment initiation among presumptive TB patients in both arms of the trial.

To evaluate the primary objective, three endpoints were defined as follows:

1) Time to initiation of TB treatment among participants tested positive for TB (either by routine TB testing or Xpert/Ultra confirmatory testing depending on the setting and scenario), is an endpoint defined as time (in days) from the patient's presentation at the first level of healthcare until the start of TB treatment.
2) Time to routine TB test performed is an endpoint measurement, i.e., AFB smear microscopy (scenario A in Fig 2), defined as time (in days) from the patient's presentation at the first level of healthcare until routine diagnostic test results were available.
3) Time to confirmatory Xpert/Ultra test performed is an endpoint measurement defined as time (in days) from the patient's presentation at the first level of healthcare until confirmatory diagnostic Xpert/Ultra results were available. In scenario A, a participant may have both routine and confirmatory tests, and the treatment will start following a positive AFB smear microscopy result; in scenario B only confirmatory Xpert/Ultra testing is available since in certain settings routine AFB microscopy testing is not performed (Fig 2).

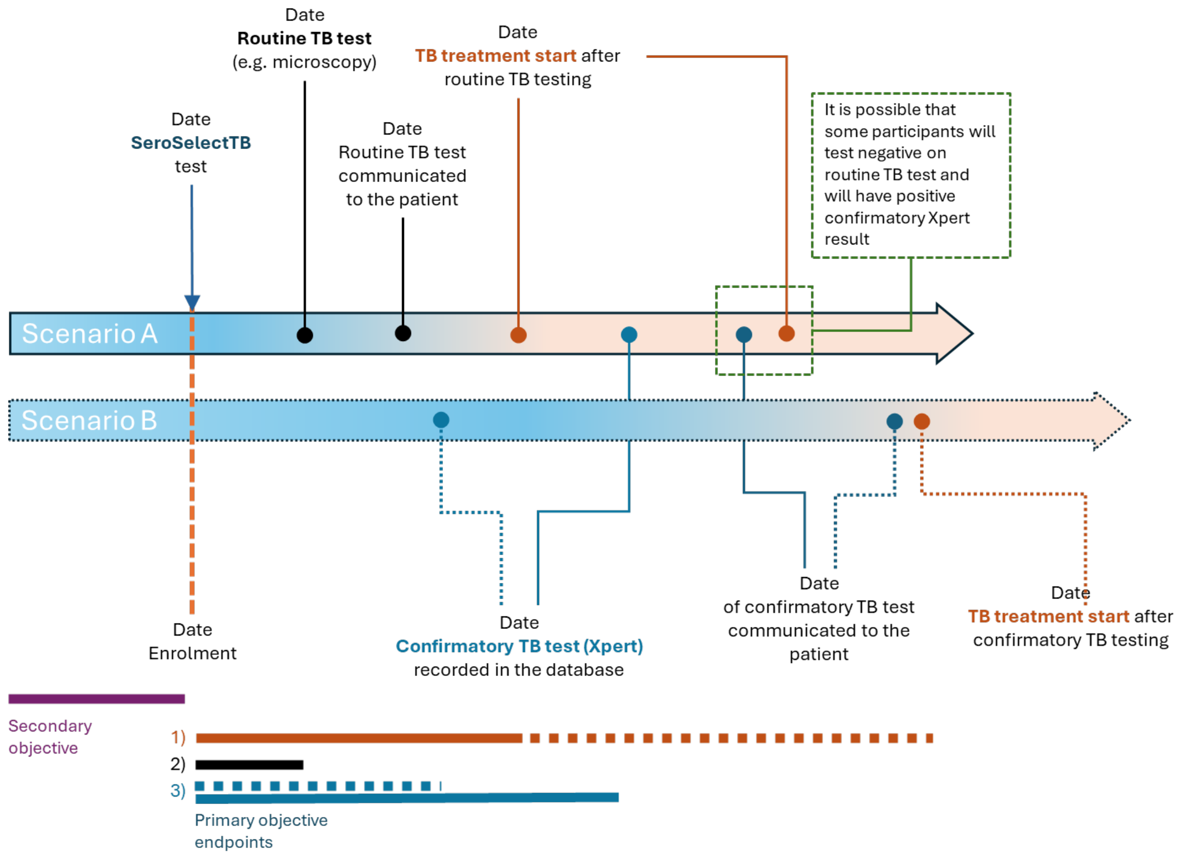

Figure 2. Graphical representation of objectives and endpoints with two possible scenarios. Scenario A (full lines) represents settings where routine AFB smear microscopy is used, scenario B (dashed lines) represents settings where AFB smear microscopy is not used and only confirmatory Xpert/Ultra testing is performed (timeline is not to scale). The date of routine and/or confirmatory Xpert/Ultra testing results are communicated to the patient should be the same date of TB treatment start. In the timelines presented above, the time gap appears longer solely for readability since the timelines are not to scale.

Variables to consider for primary objective analysis

The list is made according to fields in the electronic Case Report Form (eCRF). The numbering and wording has not been changed.

1.3 Date of Recruitment

15.1 Record date SeroSelectTB Test was done

15.9 What was the SeroSelectTB Test result

In case the SeroSelectTB Test was repeated: 15.19 Record the date the test was done

16.3 Date Xpert Ultra Test was done

16.4 What was the Xpert Ultra result

16.13 If test was positive, record the date the results were sent/communicated to participant (could be the same date as date the Xpert Ultra test was done)

17.1 Date Sputum Smear Test was done

17.2 What was the Sputum Smear Test #1 result

17.4 What was the Sputum Smear Test #2 result

17.6 If the test was positive, record the date the results were sent/communicated to participant

18.2 Record the date Culture Test was done

18.5 What was the Culture Test result

18.6 Record the time taken to obtain results (time to positivity - TTP)

18.11 Record the date the results were sent/communicated to participant

19.1 Record participant's final diagnosis or outcome

19.4 Date of TB diagnosis

22.1 Was the participant referred for anti-TB treatment

22.2 Reason for why TB treatment initiated

22.4 Record the date the participant was referred to anti-TB treatment

22.5 Date the participant started DOTS (can be the same date as above)

In practical terms, due to differences in healthcare systems in the participating countries and the national TB control program guidelines on diagnostic testing, diagnostic delay will be measured as the time between the date of the participant's enrolment (1.3 Date of Recruitment) until the date of:

1) Initiation of TB treatment – DOTS ((Directly Observed Therapy Short-Course)) (22.5 Date the participant started DOTS)
2) Sputum smear AFB microscopy test was performed, measured as the time from enrolment until the test was performed (17.1 Date Sputum Smear Test was done)
3) Xpert/Ultra test results, measured as the time from enrolment until the date of the test was performed (16.3 Date Xpert Ultra Test was done)
4) SeroSelectTB test results (15.1 Record date SeroSelectTB Test was done, which is the same as enrolment date)

• Main analytical approach

The participants will contribute person-time from inclusion in the study until routine or confirmatory diagnostic test results were available. The time to TB test results in the two groups will be represented graphically with Kaplan-Meier curves, and compared with a log-rank test. Furthermore, the two groups will be compared using Cox regression. Additional adjustment will be made for sex and age.

All analyses will be performed on a pooled data set comprised of three countries using Cox regression with shared frailty. A frailty is a latent random effect that enters multiplicatively on the hazard function. All observations within a group share the same frailty, thus the model

accounts for within-group correlation. In this case, each group is defined by participants from the same country.

In case that the above method does not converge or has other numerical issues, we will use the DerSimonian and Laird method to pool the effect estimates from each country (9).

In order to further explore the distribution of time to diagnosis, we will perform secondary analyses limited to the participants that have initiated TB treatment. We will use quantile regression to evaluate whether the median time to treatment differs between the two groups. Since those with the longest time to treatment initiation are of particular concern, we will also assess the 75th percentile. These analyses will be adjusted for gender, age, severity of symptoms, and education.

To measure the entire diagnostic delay, from the participant's presentation at the first level of healthcare until start of TB treatment, the estimates for primary and secondary endpoints will be summarized.

7 Secondary objective

To evaluate TB patient diagnostic delay by measuring time that has elapsed from the onset of symptoms until the patient self-reported to the primary health care facility.

The exploratory objective will be measured as self-reported time (in days) among participants diagnosed with TB who experienced the respiratory symptoms for a defined period of time before seeking health care (5.1 How long have you been unwell (days)).

Variables to consider for exploratory objective analysis

The list is made according to fields in eCRF. The numbering and wording has not been changed.

5.1 How long have you been unwell (days)

5.4 When did you decide to seek care

8 Tertiary objective

To evaluate SeroSelectTB test diagnostic accuracy measures, sensitivity, specificity, positive and negative predictive values, as compared to AFB smear microscopy, Xpert/Ultra and/or culture.

SeroSelectTB test accuracy will be documented in bench-top investigations conducted at Lateral Flow Laboratories (LFL) in Cape Town. Serum sample aliquots from participants in the study intervention arms in South Africa, Tanzania, and Ethiopia have been stored at LFL, and will be subjected to blinded re-testing at LFL.

The sensitivity, specificity, and positive and negative predictive value of SeroSelectTB will be evaluated as compared to AFB smear microscopy and/or Xpert/Ultra. Using an AFB smear microscopy average sensitivity of 60% we expect that SeroSelectTB (average sensitivity 90%) will detect 30% more TB cases than microscopy. Assuming a 30% increase in case detection, the minimum sample size needed for test evaluation is 42 TB cases in each group (significance level 5%, power 90%). Assay accuracy is not addressed in the statistical analyses described in this document. A larger sample size will be subjected to initial retesting for the WHO prequalification dossier including all SeroSelectTB positive samples, and equal number of SeroSelectTB negative samples, and 100 samples yielding only reactive C- and I-bands from each country. Additional samples sets may be subjected to retesting if warranted.

9 Demographic and Socioeconomic variables

Additional variables to be considered to describe the population and, where specified, adjust main statistical estimates will be extracted from eCRF in REDCap.

The list is made according to fields in eCRF. The numbering and wording has not been changed.

1.7 Category of Facility

2.3 Age (years)

2.4 Sex

2.5 Ethnic group

5.1 How long have you been unwell (days)

5.2 Were you coughing

5.3 Duration (days)

5.4 When did you decide to seek care

8.3 BMI (kg/m2)

From Questionnaire 1:

20.5 What is the highest level of education for:

20.5.1 The participant

20.7 How many people regularly sleep in your house

20.8 Does anyone in your household receive treatment for TB

Socioeconomic Indicators:

20.10 What is your electricity supply

20.11 What is your source of drinking water

20.12 What type of toilet facility is available

20.13 How many rooms are there in your house

20.14 What is the main material of the floor in your household

20.15 What is the main material of the roof in your household

20.16 What is the main material of the exterior walls of your household

20.17 Current place of residence

20.19 Do you own (include standard assets adapted to country Demographic and Health Survey)

10 Sample size calculations

Assuming an effect size (hazard ratio) of 1.1, the sample size needed to obtain a power of 90% for the log- rank test is 2314 presumptive pulmonary tuberculosis (PTB) patients in each arm of the study in each country.

Using mean diagnostic delay duration of 22 days and clinically significant 5-day difference in duration of health systems' delay (3) between the two groups, SeroSelectTB and standard-of-care, the calculated sample size is 123 TB cases in each arm of the study in each country (at the significance level of 5%, and power 90%).

Assuming that 1/26 (3), 1/23 (4) and 1/10 (5) presumptive pulmonary tuberculosis (PTB) patients have culture-positive TB as reported in 2017, we estimated that 3198, 2829 and 1230 persons with PTB needed to be screened in each study arm in Ethiopia, Tanzania, and South Africa, respectively, in order to identify 123 TB cases. However, in order to obtain the necessary power for the log-rank test, in South Africa 2314 PTB presumptive patients needed to be included in each study arm. In 2016, 7301, 28,920 and 9600 presumptive PTB patients were reported from selected study sites in Ethiopia, Tanzania and South Africa, respectively (3, 4, 5). Among presumptive TB cases 268, 1257 and 960 were bacteriologically confirmed TB cases in Ethiopia, Tanzania and South Africa, respectively.

11 Supporting documentation

Clinical data will be collected using a structured questionnaire (optimized for electronic data capture using Survey Solutions tools: https://mysurvey.solutions), and socioeconomic indicator-measures will be collected using 'Tool to Estimate Patients' Costs' (7).

(https://pdf.usaid.gov/pdf_docs/PNADP992.pdf ; 8_Tool to estimate patient costs_Guidelines interpretation of results_final ; TB Patient Cost Survey Dissemination 30-06-2018)

12 References

1. Pantoja A, Fitzpatrick C, Vassall A, Weyer K, Floyd K. Xpert MTB/RIF for diagnosis of tuberculosis and drug-resistant tuberculosis: a cost and affordability analysis. Eur Respir J. 2013;42(3):708-20.
2. Pantoja A, Kik SV, Denkinger CM. Costs of novel tuberculosis diagnostics--will countries be able to afford it? J Infect Dis. 2015;211 Suppl 2: S67-77.
3. Yimer S, Holm-Hansen C, Yimaldu T, Bjune G. Evaluating an active case-finding strategy to identify smear-positive tuberculosis in rural Ethiopia. Int J Tuberc Lung Dis. 2009;13(11):1399-404.
4. Nyombi B. Personal communication. 2017.
5. Theron G. Personal communication. 2017.
6. Mauch V, Woods N, Kirubi B, Kipruto H, Sitienei J, Klinkenberg E. Assessing access barriers to tuberculosis care with the tool to Estimate Patients' Costs: pilot results from two districts in Kenya. BMC Public Health. 2011; 11:43.
7. TBCTA USAID. The Tool to Estimate Patients' Costs, 2008 [Available from: Opmaak 1]
8. Cleophas EP, Cleophas TJ. Clinical research: A novel approach to regression substitution for handling missing data. Am J Ther. 2013;20(5):514–9. 10.1097/MJT.0b013e3181ff7a7b
9. Knapp G, Hartung J. Improved tests for a random effects meta-regression with a single covariate. *Stat Med*. 2003; **22**(17): 2693-2710. doi:10.1002/sim.1482
10. D.R. Cox Regression models and life-tables J. R. Stat. Soc.: Series B (Methodological), 34 (1972), pp. 187-202
11. M.J. Bradburn, T.G. Clark, S.B. Love, D.G. Altman Survival analysis part II: multivariate data analysis–an introduction to concepts and methods Br. J. Cancer, 89 (2003), pp. 431-436
12. A. Scherer, N.V. Wünderlich, F. von Wangenheim The value of self-service long-term effects of technology-based self-service usage on customer retention MIS Q., 39 (2015), pp. 177-200
17. T.A. Balan, H. Putter A tutorial on frailty models. Stat. Methods Med. Res., 29 (2020), pp. 3424-3454
