# Supplementary material for: Feasibility, accuracy, and effect of a rapid point-of-care serological test (SeroSelectTB) to identify presumptive pulmonary TB patients for confirmatory testing in Ethiopia, South Africa, and Tanzania: a multicenter, open-label, parallel-group, randomized, controlled trial
Source: eClinicalMedicine. 2026 Apr 25;95:103914. doi: 10.1016/j.eclinm.2026.103914 (PMC13129460; doi:10.1016/j.eclinm.2026.103914)
Supplement: LD3366 SEROSELECTTB PACK INSERT FINAL rev 008_6 June 2024 [file mmc7.pdf]

LATERAL FLOW
LABORATORIES

SeroSelectTB

INTENDED USE

The Lateral Flow Laboratories **SeroSelectTB** test is a qualitative *in vitro* testforthedetectionof antibodies associatedwith active pulmonary tuberculosis,usinghumanwholebloodassampleinadult population. The testisforperformanceevaluationonlyandis intendedfor professionaluse in a point-of-caretriage testing situation,toaidinthe early identification of tuberculosis infection.

INTRODUCTION

New diagnostic tools are essential to curb the global tuberculosis (TB) pandemic. The lack of a rapid and widely accessible triage test that is easy to perform without laboratory facilities is one of the main reasons for unacceptabledelayindiagnosisandtreatment.A rapidtriage testusedat health-postswill hastensame-dayreferral,confirmatory testing, andtimelyTBtreatment. Expedient diagnosis willreduce the pool of infectionbelow the critical reproductive rate for *Mycobacterium tuberculosis* (Mtb)and potentiallycurbtheTB pandemic. LFLhas developeda rapid serologicaltriagetest, **SeroSelectTB**,forthe detection of active TB that is suitable for use in high burden countries. It is crucial thatatriage testforTB detects onlyactivedisease because latentTB infection isprevalent, butnot atargetfortreatmentinhigh burden countries (HBC). Using laboratory-based immunochemical techniques, antigens recognized by antibodies from patients and community controlsrecruited from TB endemic regions have been compared, andcombinations that differentiate between active disease andlatentinfectioneveninthecontextofHIVco-infectionhavebeen identified.

TEST PRINCIPLE

The Lateral Flow Laboratories **SeroSelectTB** is a rapid lateral flow test for thequalitativedetectionof humanantibodies. Theassayuses a nitrocellulose membrane strip coated with immobilized combinations of high affinity TB antigens on the two test lines as a solid phase, a confidence indicator line, and a controlline.Specific colloidal goldconjugates areutilized as sandwich pairs for the capturelines, allowingfor easily identifiable colored linesfor diagnostic purposes. The controlline is a non-specific ChickenIgY and Goat anti-Chicken.Oncethe patient sample has beenapplied to the sample pad, a chase bufferis added to the test. The buffer migration releases the dried conjugates whichinteract with the patient sample and progress chromatographically up the nitrocellulose strip. If the patient sample carries antibodies targeted against TB antigens, a completed sandwich will form, leading to the generation of a visible colored line. None of the confidence indicator line, the TB lines or the control line are visible before applying patient specimen and initiating the test with the chase buffer. The control line is used for procedural control and shows onlythat thediluent has been appliedsuccessfullyandthat buffer/conjugate migrationhastakenplace.

ACTIVE INGREDIENTS OF MAIN COMPONENTS

1 test strip includes: Colloidal gold conjugates: TB recombinant antigens, C I antigens, IgY.
Immobilized capture antibodies coated on nitrocellulose. Specific TB antigens, confidence indicator antigen, IgY antibody.

- Assay chase buffer includes surfactants, buffers and Proclin 0.1% as preservative.

MATERIALS PROVIDED

The Lateral Flow Labs **SeroSelect TB** test kit contains the following items to perform theassay:

- 25Individual foil pouches, containing 1 test deviceand 1 desiccant
- 1 Assay diluent vial (5 ml)
- 25 Specimen transferdevices (20 ul)
- 25 Sterile lancets
- 25 Alcohol swabs
- Instruction for use (IFU)

MATERIALS REQUIRED BUT NOT PROVIDED

- Timer,Gloves,Sterile gauze, or cotton
- Collectionby venipuncture: Venipunctureblood collectiontube, Micropipette, Pipette tips

KIT STORAGE AND STABILITY

1. Thetestkit should bestoredat temperaturebetween4°C and 30°C. Do not exposethe kit or its components to freezing or high temperatures
   Note: Whenstoredin refrigerator, all kit components must be brought to roomtemperature(15 - 30 °C) a minimum 30 minutes prior to performingthe test. Do not open the pouch whilst componentscome to room temperature.
2. Assay diluent cap should be kept firmly sealed between each use.
3. Afterfirst opening of the assay diluent bottle, assay diluent is stable until the expiration date, if kept at 4 - 30 °C.
4. Thetestdeviceis sensitive tobothheat and humidity.
5. Perform the test immediately after opening the foil pouch, to avoid exposure to humidity and moisture.
6. Theshelf life of the kit is as indicated onthe outer package.
7. Do not use the testkit beyondits expiration date.
8. Do not usethe testkit if the pouch is damaged orthe seal is broken.

WARNINGS

1. For *in vitro* diagnostic use only. Do not re-use the test deviceand kit components.
2. The instructions must be followed exactly to achieve accurate results. Any individual using this product must be trained in its use and interpretation.
3. Do not eat or smoke while handling specimens and kit.
4. Wearprotectivegloveswhilehandlingspecimensandwashhands thoroughlyafterwards.
5. Clean up spills thoroughly using an appropriate disinfectant.
6. Decontaminateanddisposeofallspecimens,tested devicesand potentiallycontaminated materials (i.e.,specimentransfer device, test device) in a biohazard container as if they were infectious waste.

PROCEDURE

[Precautions]
Bring the kit components to room temperature (between 15 °C and 30 °C) before use.
Remove **SeroSelectTB** rapid device from foil pouch only when ready to test. Perform the test immediately after opening the foil pouch, to avoid exposure to humidity and moisture.
Use a new specimen transfer device for each specimen to avoid cross contamination of specimens, which could produce erroneous results. Discard the lancet or alcohol swab if the package is pierced or damaged. The item may no longer be sterile; there is a risk of infection if used.

TEST PROCEDURE

Before starting this process, have the components of the test available and open the device.

- Step 1: Clean the area to be lanced with the alcohol swab provided
- Step 2: Twist the lancet cap more than 180 degrees and remove the cap.
- Step 3: Place the end face of the lancet on the selected finger site and apply pressure until you hear a click sound, remove the lancet, and dispose of safely.
- Step 4: Collect the whole blood from the finger by holding the cup to the finger and allowing the cup to fill. Slight pressure may be applied to the finger to ensure enough blood is available for collection.
- Step 5: Transfer sample to the rear sample port, allowing the blood to fully drain onto the sample pad. In the case of serum sample, transfer to front sample port.
  Note: *Before applying buffer, two light blue lines will be visible, one at the base of the read window, and the second in the control line position. These will wash through(disappear) as the test is running.*
- Step 6: Add 5 drops of diluent from provided bottle of test fluid into sample port. An air bubble does not count as a drop. If test does not start within a minute add another drop of buffer.
  Caution: Do not let bottle nozzle touch device to avoid cross-contamination. Hold bottle vertically while dispensing. If you do not hold the bottle vertically, it can lead to erroneous results.
- Step 7. Allow 15 minutes before interpretation of results.
  Caution: Do not read test results after 30 minutes; reading after 30 minutes can yield erroneous results.

NOTE:
For specimen collection by Venipuncture

- Using venipuncture, draw whole blood into the collection tube
- Whole blood specimens should be tested as soon as possible after collection.
- Apply the blood specimens to the round sample well of the test device using a micropipette set at 20 µl.
- Continue with Steps 5,6 and 7 above

Required (provided):
Test device, Swabs
Lancet
Specimen transfer device
Required (Not provided):
Timer, Protective gloves
Sterile gauze or cotton
Biohazard disposal unit

Step 1:
Clean finger

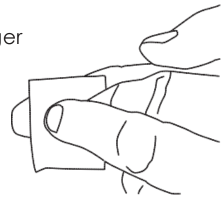

Step 2:
Twist open lancet.

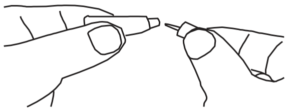

Step 3:
Prick and dispose.

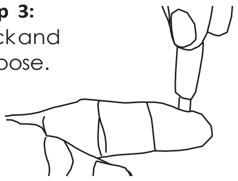

Step 4:
Collect blood from finger. Allow cup to fill.

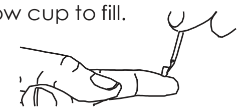

Step 5: for WHOLE BLOOD
Transfer blood to **rear** sample port.

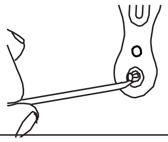

Step 6:
SERUM

Add 10ul of serum into **front** sample port.

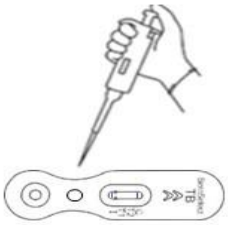

Step 7: Add 5 drops of diluent into sample port. If test does not start within a minute, add another drop of buffer.

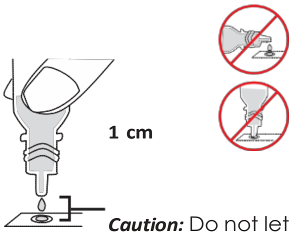

Caution: Do not let bottle nozzle touch device. Hold bottle vertically while dispensing. An air bubble does not count as a drop.

Step 8:
Allow 15 minute before interpretation.

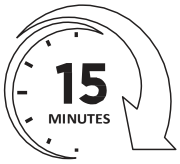

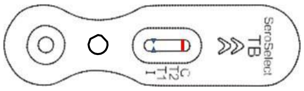

NEGATIVE –

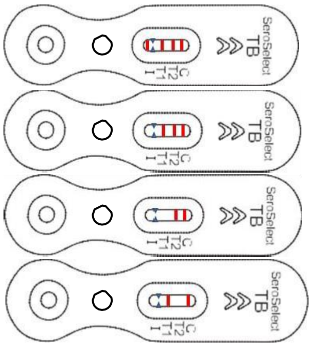

POSITIVE +

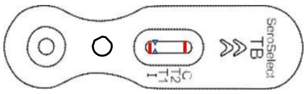

REFER FOR FURTHER TESTING:
A POSITIVE CI RESULT MAY REDUCE RELIABILITY OF THE TB TEST LINES, TREAT AS POTENTIALLY TB POSITIVE

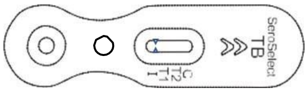

INVALID

IN-HOUSE SERUM TESTING PERFORMANCE

			LFL RESULT POS	LFL RESULT NEG
**ETHIOPIA**	+	21	17	4
	-	28	8	20
**TANZANIA**	+	58	54	4
	-	57	10	47
**SOUTH AFRICA**	+	16	15	1
	-	29	6	23

**Combined sensitivity**	91%
**Combined specificity**	79%

vs: Culture and GeneXpert

HEALTH AND SAFETY

- Ensure appropriate disposal of test material as recommended in this procedure.

1. **Negative result:**
   The presence within the result window of only one colored line at "C" Control line indicates a negative result.
2. **Positive result:**
   The presence within the result window of either 1 or 2 colored lines at "T1" and/or "T2" indicates a positive result. The presence of a line at the lowest position "I" together with colored lines at "T1" and/or "T2" indicates a positive result.
3. **A Positive C I Result:**
   The presence of a line in the lowest position "**I**" without any colored lines in position "T1" and "T2" should be referred for confirmatory testing. This reflects a potential decrease in TB signal in the presence of immunosuppression. This should be treated as potentially positive.
4. **Invalid result:**
   When interpreting the test, if no line is visible in the result window at "C" Control line, the result is invalid. The directions may not have been followed correctly or the test may have deteriorated. It is recommended that the patients should be retested using a new test device and with a new sample if tested on a fingerprick whole blood.

TEST LIMITATIONS

1. The test procedure, precautions and interpretation of results for this test must be followed precisely.
2. The test is limited to the detection of antibodies to pulmonary TB. Although the test is very accurate in detecting these antibodies, a low incidence of false results can occur. Other clinically available tests are required if questionable results are obtained. As with all diagnostic tests, a definitive clinical diagnosis should not be based on the results of a single test but should only be made determined after all clinical and laboratory findings have been evaluated. Samples that test as potentially positive should be referred for confirmatory testing via PCR or bacterial culture.
3. This test is applicable to a general population in which *tuberculosis* is prevalent. It is not validated for other populations, pregnant women, children, or neonates.

PROCEDURAL CONTROL

The test device has a "C" printed on the surface of the device adjacent to where the control line may appear. None of the test lines at "I" C I, Test line 1, "T2" Test line 2 nor "C" Control line should be visible in the result window before applying either specimen or diluent. The "C" Control is used for procedural control and shows only that the diluent has been applied successfully and that the migration of the buffer and conjugate has occurred.

MANUFACTURER

Lateral Flow Laboratories (Pty) Ltd
1 / 2 Greenwich Place, Capricorn Park
Muizenberg, Cape Town, 7948 South Africa
www.lateralflowlabs.com

IVD		30°C / 4°C			RUO	i
INVITRO DIAGNOSTIC MEDICAL DEVICE	PROTECT FROM MOISTURE	STORAGE TEMPERATURE 4 - 30°C	DO NOT USE IF PACKAGE IS DAMAGED	DO NOT REUSE	RESEARCH USE ONLY	CONSULT INSTRUCTION FOR USE

P.No: TUB-AB-014 Rev 008 | NOT FOR SALE | 06 JUNE 2024 | FOR EVALUATION ONLY
