# Supplementary material for: Feasibility, accuracy, and effect of a rapid point-of-care serological test (SeroSelectTB) to identify presumptive pulmonary TB patients for confirmatory testing in Ethiopia, South Africa, and Tanzania: a multicenter, open-label, parallel-group, randomized, controlled trial
Source: eClinicalMedicine. 2026 Apr 25;95:103914. doi: 10.1016/j.eclinm.2026.103914 (PMC13129460; doi:10.1016/j.eclinm.2026.103914)
Supplement: ICF SeroSelectTB_REK_template for translation_140920 [file mmc8.pdf]

Informed Consent Form

This Informed Consent Form is for adults who have self-reported to this healthcare facility and are invited to participate in the clinical evaluation of a new rapid test for tuberculosis: SeroSelectTB.

The title of our research project is "Evaluation of the feasibility, accuracy, and effect of a rapid point-of-care serological triage test for active TB (SeroSelectTB) in high burden, HIV-endemic African settings: a multi-centre, parallel-group, randomised, controlled trial”. We call the test and the project “SeroSelectTB”.

Name of Principal Investigator [to be added for South Africa, Tanzania or Ethiopia]: Professor Grant Theron, Dr Balthazar Nyombi or Dr Kidist Bobosha

Name of Organization [to be added for South Africa, Tanzania or Ethiopia]: Stellenbosch University (South Africa), Kilimanjaro Christian Medical University College (Tanzania), or Armauer Hansen Research Institute (Ethiopia)

Name of Sponsor: Norwegian Institute of Public Health, Norway

Name of Proposal and version: SeroSelectTB EDCTP RIA2018D-2493

This Informed Consent Form has two parts:

- Information Sheet (to share information about the research with you)
- Certificate of Consent (for signatures if you agree to take part)

You will be given a copy of the entire Informed Consent Form

PART I: Information Sheet

Introduction
I am a healthcare provider working for (add partner affiliation). We are investigating a new rapid diagnostic test for tuberculosis (TB) that is suitable for use in this healthcare facility. I am going to give you information and go through the Community Engagement brochure, and thereafter invite you to be part of this research.

If you have questions please ask at any time.

Purpose of the research
A new rapid test for TB has recently been developed. This test will help us quickly identify and properly treat persons with TB disease. We are doing this research to determine if this new test is suitable for use at our healthcare facility.

Type of Research Intervention
This research will involve taking one blood sample from your arm and two saliva samples from your mouth, and an interview/answering a questionnaire.

Participant selection
We are inviting all adults who self-report to this healthcare facility with symptoms indicative of TB to participate in the research on this new TB test.

Voluntary Participation
Your participation in this research is voluntary. It is your choice whether to participate or not. All the services you receive at this healthcare facility will continue and nothing will change whether you choose to participate or not. If you choose not to participate in this research project, you will receive the service that is routinely offered at this healthcare facility for TB.

Randomization
We do not know if the rapid test will make a difference to how you receive your healthcare. To find out, we must compare what happens to people who get the test compared to people who do not get the test. You will be randomly assigned to either the group receiving the rapid test or the group receiving the standard of care. This means there is a 50/50 chance that you will be in the group that receives the test. However, you will receive exactly the same healthcare services in both groups.

Procedures and Protocol
We will take one blood sample from your arm using a sterile syringe and needle. We will take about this much blood *(Note to healthcare worker: show a vial or other small container with a small amount of water in it).* We will also take two saliva samples using this collection device that is simply placed in each side of your mouth between the gums and cheek *(Note to healthcare worker: show a MucoSafe saliva collection device)*.

Description of the Process
Today we will complete a questionnaire together, and ask you to give us one blood sample and two saliva samples.

- If you are randomized in the test group:
    - A small volume from your blood sample will be tested on the rapid TB test at once at this healthcare facility while you wait.
    - The test result will be ready in 10 minutes.

    - If your rapid test is positive you will be referred to the next level of healthcare (add name of clinic/hospital) for confirmatory testing and clinical evaluation.
    - If and when your rapid test result is confirmed you will start treatment according to the National TB Control Program guidelines.
    - You will be followed-up for 6 months in accordance with the National TB Control Program guidelines.
- If you are randomized into the standard-of-care group:
    - You will be evaluated based on your clinical symptoms here at this facility.
    - Based on the results of your clinical evaluation, you will either be referred to the next level of healthcare (add name of clinic/hospital) or receive the standard-of-care for your condition at this facility.
    - If you are referred to the next level of healthcare, you will receive routine TB testing. If your results are positive you will start treatment according to the National TB Control Program guidelines.
    - If you receive treatment you will be followed-up for 6 months in accordance with the National TB Control Program guidelines.

Data and samples

The information collected during the interview will help us find out if the rapid test gives faster diagnosis and treatment than the standard-of-care. We also want to know if using the rapid test will save money as compared to the standard-of-care. All samples will be coded and your name will not be on the sample. After testing, we will store your sample in a freezer called a biobank. The freezer is always locked. Your sample may be used again in the future if we need repeat the test for any reason.

Risks

There are no risks associated with participating in this research.

Benefits

If you participate in this research, you will help us determine if this new test should be implemented at all healthcare facilities like this one in the future. We believe that implementing a new rapid test for TB at healthcare facilities will help all persons get their diagnosis and treatment quicker than if their blood is not tested here. Using a rapid test at this healthcare facility in the future may help to reduce the spread of TB to your family and in the community.

Confidentiality

It is possible that others in the community will become aware of this project and may ask you questions. Please feel free to discuss this project and share the Community Engagement materials you have received with others. We will not be sharing the identity of those participating in the research.

The information that we collect from this research project will be kept confidential. Information about you collected during the research will be put away and no one except the researchers will be able to see it. Any information about you will have a number on it instead of your name. Only the researchers will know what your number is (your code) and we will lock that information up with a lock and key. Only the information about your health and test results will be shared with your clinician or doctor to ensure you get the best treatment.

Sharing the Research Results

The knowledge that we get from doing this research will be shared with you through community meetings before it is made widely available to the public. Confidential information will not be shared. There will be small meetings in the community and these will be announced. After

these meetings, we will publish the results in order that other interested people may learn from our research.

Right to Refuse or Withdraw

You do not have to take part in this research if you do not wish to do so. You may also stop participating in the research at any time you choose. It is your choice and all of your rights will still be respected.

This proposal has been reviewed and approved by [name of the local IRB], which is a committee whose task it is to make sure that research participants are protected from harm. If you wish to find more about the IRB, contact [name, address, telephone number.]). It has also been reviewed by the Regional Committee Medical and Health Research Ethics in South East Norway for the Norwegian Institute of Public Health, the study sponsor, and by the Ethical Review Committee of the European Developing Clinical Trials Partnership (EDCTP), the funder.

You may ask me any questions about any part of the research study if you wish. Do you have any questions?

PART II: Certificate of Consent (filled out in two copies)

Participant:
I have read the information or it has been read to me. I have had the opportunity to ask questions about the research and my participation, and any questions that I have asked have been answered to my satisfaction. I consent voluntarily to participate as a participant in this research.

Print Name of Participant__________________

Signature of Participant ___________________

Date ___________________________
Day/month/year

Witness if participant is illiterate:
I have witnessed the accurate reading of the consent form to the potential participant, and the individual has had the opportunity to ask questions. I confirm that the individual has given consent freely.

Print name of witness_____________________ AND Thumb print of participant

Signature of witness ______________________

Date ________________________
Day/month/year

Statement by the healthcare worker taking the consent:
I have accurately read the information sheet and Community Engagement materials to the potential participant, and to the best of my ability made sure that the participant understands that the following will be done:

1. One blood sample and two saliva samples will be taken
2. A small volume of blood will be tested on the new rapid TB test
3. If the test result is positive, the participant will be referred at once for same-day confirmatory testing and, if warranted, treatment

I confirm that the participant was given an opportunity to ask questions about the study, and all the questions asked by the participant have been answered correctly and to the best of my ability. I confirm that the individual has not been coerced into giving consent, and the consent has been given freely and voluntarily.

A copy of this ICF has been provided to the participant.

Print name of healthcare worker taking the consent_______________________

Signature of health care worker taking the consent__________________________

Date ___________________________
Day/month/year
